# Introduced Fish in Mountains: State of Published Research on their Removal from Lentic Ecosystems

**DOI:** 10.1007/s00267-026-02616-9

**Published:** 2026-09-09

**Authors:** Dan Cogălniceanu, Florina Stănescu, Teodora L. Tănase, Sabina E. Vlad, Mark Snethlage, Ajay Ranipeta, Davnah Urbach

**Affiliations:** 1 Ovidius University of Constanţa, Research Center of the Natural Sciences Department, Constanţa, Romania; 2 Ovidius University of Constanţa, Center for Morphological and Genetic Studies of Malignant Pathology (CEDMOG), Constanţa, Romania; 3 Asociația Chelonia România, Bucharest, Romania; 4 Global Mountain Biodiversity Assessment, University of Bern, Institute for Plant Sciences, Bern, Switzerland; 5 Centre Interdisciplinaire de Recherche sur la Montagne, University of Lausanne, Lausanne, Switzerland; 6 Binomal LLC, New Haven, CT USA

**Keywords:** Aquatic ecosystems, Fish stocking, Invasion biology, Mountain ecosystems, Removal methods

## Abstract

The introduction of alien fish in freshwater ecosystems has become an increasing concern over the last decades and a considerable amount of literature exists on their ecological consequences. However, less is known about their management and eradication, particularly in mountain ecosystems, where fish introductions have been carried out over decades. Here, we systematically mapped and reviewed peer-reviewed literature on fish removal in mountain ecosystems, with a focus on standing waters. The results of our work indicate important spatial and taxonomic biases, with most published studies performed in North America and on salmonids. The removal methods were primarily physical (57%) and chemical (31%), with variable success rates depending on which approach or combination of approaches were used, the species targeted, and ecosystem characteristics. Based on the 35 publications we reviewed, there is no “silver bullet” for fish removal from mountain standing waters. Prevention, early detection, and rapid intervention remain the best approach in halting the spread of or eradicating invasive species before the establishment phase. Making use of the available data and learning from past interventions remains the most affordable approach to guide and adapt management options. To share the knowledge we collected, inform management, and raise awareness, we developed the “FishME Toolbox” as an online open access infrastructure offering tailored resources for both expert and non-expert users.

## Introduction

Alien species have established themselves in nearly every biome, ecosystem, and habitat on Earth (Peller and Altermatt 2024) and represent one of the five major threats to biodiversity (IPBES 2019; Roy et al. 2024a). Of the approximately 40,000 known alien species (Bacher et al. 2025), almost 10% are considered invasive based on their documented negative impacts (Roy et al. 2024a). Alien species are widespread in terrestrial and aquatic ecosystems alike. In freshwater ecosystems, they represent a growing threat (e.g., Polce et al. 2023; Karatayev and Burlakova 2025) and economic burden (e.g., Kouba et al. 2022; Macêdo et al. 2022), and place increasing pressure on policy-makers, resource managers, as well as scientists (see Simberloff et al. 2013).

Of all alien freshwater species, fish represent one of the most widely introduced taxonomic groups (Gozlan 2008; Gozlan et al. 2010; Bernery et al. 2022). The history of their introductions dates back thousands of years in China (Zhao et al. 2015) and Europe (Balon 1995) but introduction rates have substantially increased since the 18th century industrial revolution (Slobodníková et al. 2023). In recent times, introduction rates have increased primarily as a consequence of aquaculture, trade, infrastructure development, unintentional releases, deliberate releases of bait fish for angling, stocking for sports fishing, and biological control (see Haubrock et al. 2021; Bernery et al. 2022). Ecological impacts of fish introductions are numerous and include the displacement and extinction of native species, the alteration of trophic interactions and food webs, the disruption of ecosystem functioning and processes such as nutrient cycling, habitat modification and degradation, predation, competition, hybridization, as well as disease and pathogen transmission (see Gozlan et al. 2010; Cucherousset and Olden 2011; Haubrock et al. 2021, 2025; Bernery et al. 2022; Britton 2023). Economic consequences, in turn, are equally important and affect public and social welfare as well as fisheries, authorities, and stakeholders through damage and resource losses as well as management-related expenditures (Haubrock et al. 2021).

Mountain ecosystems are no exception to the widespread occurrence of freshwater fish introductions (Roy et al. 2024b) and to the resulting long-lasting environmental degradation (e.g., Knapp and Matthews 1998; Beaulieu et al. 2021). Despite the remoteness of mountains and the general belief that they are minimally influenced by human activity, the introduction of game fish such as trout (e.g., *Oncorhynchus* spp., *Salmo* spp., and *Salvelinus* spp.) to mountain lakes dates as far back as the Middle Ages in the Pyrenees (see Ventura et al. 2017) and the 15th century in the Alps (Schabetsberger et al. 2023). Such introductions are still being reported across the world, and often promoted by various administrations, state fisheries agencies as well as fishing organizations (Pister 2001; Schindler and Parker 2002). In western North America, for instance, more than 95% of larger (>2 ha surface area), deeper (>3 m maximum depth), high-elevation lakes contain non-native trout (Bahls 1992). In the Pyrenees, in turn, invasive fish occur in 35–85% of the lakes (Miró and Ventura 2013). Due to their ecological impact and their extent worldwide, introduced invasive fish are possibly the most critical threat to mountain lake biodiversity (Knapp et al. 2001; Ventura et al. 2017), with numerous reports of declines in native communities of fish (e.g., Shepard et al. 2005; Budy et al. 2021), amphibians (e.g., Miró et al. 2018; Budy et al. 2021; Bello et al. 2024), invertebrates (e.g., Schnee et al. 2021; Osorio et al. 2022), and zooplankton (e.g., Knapp and Sarnelle 2008), as well as modifications in the food-web structure (e.g., Detmer and Lewis Jr. 2019), and the ecological status and visual appearance of the aquatic ecosystems (see Pou-Rovira et al. 2019). Through the accumulation of relatively high levels of atmospherically-deposited toxins, non-native fish introduced in previously fishless lakes also represent a threat to the health of both the local wildlife and the anglers consuming them (see Chiapella et al. 2018).

Various strategies exist for managing freshwater fish introductions, and the choice of technique depends on the introduced species, the stage of the invasion, as well as on ecosystem characteristics like the bathymetric profile, ecosystem structure, the presence of submerged vegetation, connected streams or torrents, and the native species composition (see Donaldson and Cooke 2016; Pou-Rovira et al. 2019; Bernery et al. 2022). However, the connectivity of water bodies and the challenges associated with their permeability make the management of introduced species particularly difficult. Preventive measures, along with early detection and swift responses, are effective in minimizing the risk of establishment (Bernery et al. 2022). Yet, once the invasion progresses, more intensive methods are needed (Robertson et al. 2020; Britton et al. 2023). These methods consist of containment, control, or species eradication (Britton et al. 2011; Donaldson and Cooke 2016; Rytwinski et al. 2019). Containment, for instance with physical barriers, is typically difficult in highly connected aquatic ecosystems. Eradication (see Meronek et al. 1996; Rytwinski et al. 2019; Bernery et al. 2022), i.e., the elimination of whole fish populations from the body of water in which they are encountered, can be achieved through piscicides such as rotenone, antimycin, saponins and niclosamide, harvest regimes (e.g., intentional over-fishing), physical removal (e.g., traps, electrofishing, nets), deoxygenation (e.g., dry ice, sugar, molasses, whole milk, carcass material and carcass-analog pellets), whole lake drainage or biological control (e.g., introduction of predators, intraspecific (genetic) manipulation). However, these efforts often fail to fully eliminate the targeted species and result in population control or temporary population reduction (see Meronek et al. 1996; Rytwinski et al. 2019).

The legal frameworks for the management of introduced fish species are presented in detail in the IUCN online guide on designing legal and institutional frameworks on alien species (Shine et al. 2000). At the pan-continental scale, the European Regulation 1143/2014 on Invasive Alien Species includes 30 freshwater species that are listed as priorities for introduction prevention and invasion management (Britton et al. 2023). In the United States, mountain lake fisheries management is part of the Wilderness Act of 1964, but vague policy language has resulted in conflicts and confusion across federal land management and regulatory agencies (Chiapella et al. 2018). In view of the complexity of aquatic ecosystems and the different rates of recovery of different species and ecosystem compartments, quantifying the success of introduced fish management programs is often difficult and typically requires long-term evaluations and assessments tailored to the specific management objectives (Rytwinski et al. 2019).

Several reviews exist on fish management measures as well as on non-native fish eradication and population control methods (see Rytwinski et al. 2019 for a recent review). However, none focuses specifically on introduced fish species in lentic mountain ecosystems. We fill this gap and systematically map and review the available peer-reviewed literature on their management. Specifically, our objectives were to (1) determine the geographic and thematic coverage of peer-reviewed studies and identify gaps, (2) determine what methods are applied for the removal of fish in the mountain context specifically, and (3) assess their effectiveness. Throughout the text, we use the concepts of lentic ecosystems and lakes interchangeably, even though the literature we reviewed also included other types of standing waters than lakes (e.g., ponds). To share the knowledge we collected, raise awareness, inform agencies responsible for the choice and implementation of management campaigns, and address issues of knowledge accessibility in conservation practice (see Sabo et al. 2024), we further developed the “FishME Toolbox” as an online open access infrastructure offering tailored resources for both expert and non-expert users (https://mountainbiodiversity.org/projects/FishME/).

## Methods

### Literature Search Strategy and Identification of Publications

**Fig. 1:**
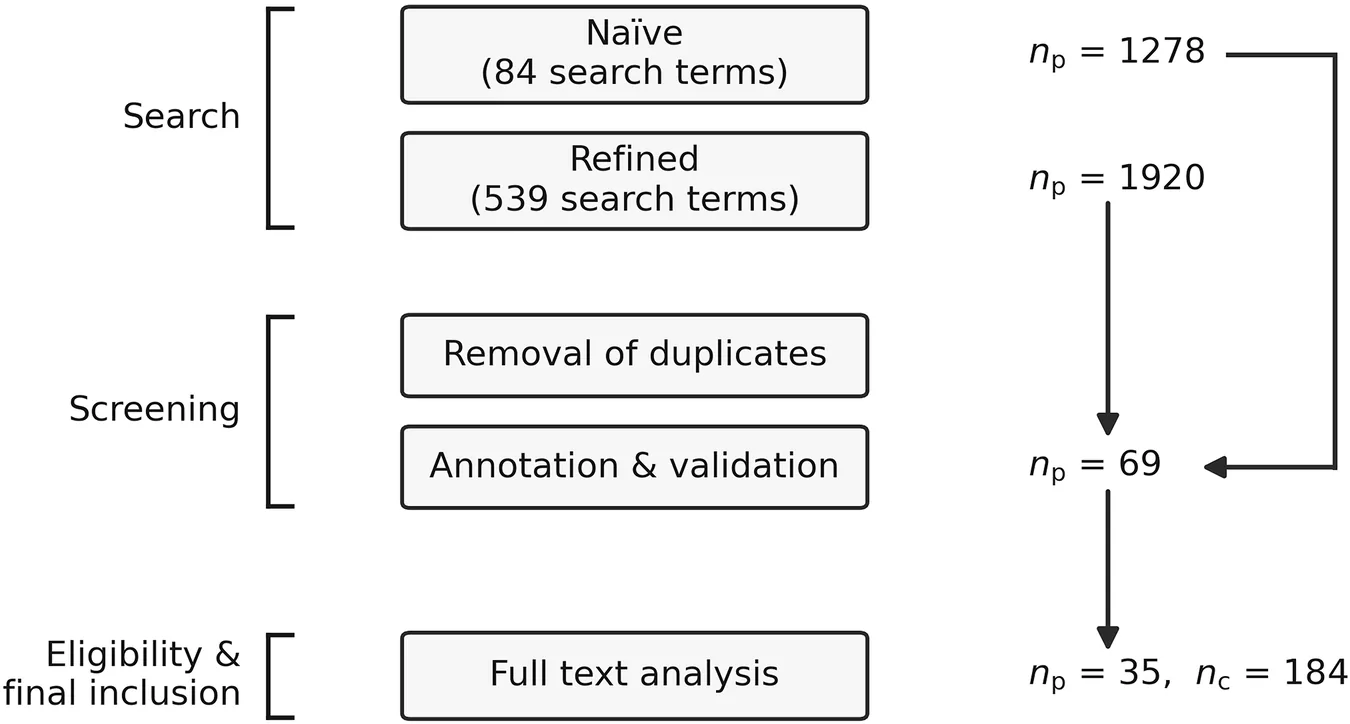
Overview of the steps taken during article selection (n_p _= number of papers, n_c _= number of cases, i.e., unique combinations of single species in a single location). These steps map onto those of the PRISMA guidelines (Moher et al. 2009)

The systematic mapping of published research on introduced fish and their removal in mountain lakes followed a standardized systematic review protocol (e.g., Donaldson and Cooke 2016). The search in Web of Science, which we performed on July 2nd 2024, was limited to scientific publications in English with a focus on the title, author keywords, and abstract (Fig. 1). The development of the search string and the searches followed a two-step approach (Grames et al. 2019). For the first, naïve search, we used a limited set of 84 search terms provided by the authors of this study, based on their expert knowledge, and grouped them into concept categories that we subsequently combined, following Grames et al. (2019). The categories we used consisted in ecosystem types (e.g., lake, loch, reservoir), management categories (e.g., removal, extraction, eradication), management methods (e.g., mechanical, biological, chemical), and fish species (both vernacular and scientific names, e.g., salmonid, cypriniform, trout, *Salvelinus fontinalis*). The first search string further included a set of general terms (e.g., introduced, fishing, invasive). The naïve search terms are listed in Supporting Information—Table S1.

For the second, refined search, we used a search string that consisted primarily of selected keywords extracted from the title, abstracts, and author keywords of the publications obtained through the naïve search. We extracted the keywords with the package *litsearchr* (Grames et al. 2019) in R v. 4.4.1 (R Core Team 2024) and used them to create 539 unique search terms associated with ecosystem characteristics, species and species interactions, drivers of fish introduction, fishing activity, management categories and methods, survey methods, and ecological impacts. Of these search terms, 343 were selected for inclusion and combined in the refined search string (Table 1), according to the specific focus of the project, while the complete search term list was retained for subsequent annotation and relevance scoring. To help limit the search to mountain lentic ecosystems and improve species coverage, we further added geographic search terms as well as species names to the search string. Geographic search terms were taken from the hierarchically organized “GMBA Mountain Inventory” (Snethlage et al. 2022). Scientific names, in turn, were extracted from the results of the naïve search using the “Global Names Finder” app (Mozzherin et al. 2024) and checked against the “GBIF backbone taxonomy” using the RGBIF package (Chamberlain et al. 2025). The refined search terms are provided in Supporting Information—Database 1, and the search string in Supporting Information—Table S2.

**Table 1:** Structure of the refined search string with examples of search terms (see Supporting information—Database 1 for the full list of search terms). Note that the mountain terms were only included in the refined search

Search in	Search term category	Example search terms in search string	Boolean
Title, abstract, author keywords	Mountain terms	*mountain$ OR alpine OR carpathian$ OR …*	*AND*
	Ecosystem terms	*lake$ OR pond$ OR tarn$ OR …*	*AND*
	Fish species terms	trout$ OR salmoniform* OR “Oncorhynchus mykiss” OR …	*AND*
	Management terms	eradication$ OR electrofish* OR rotenone OR …	

### Article Screening

Given the search terms we used, the search results also included publications not relevant for the review, such as articles about fish introductions. Therefore, the next step of our workflow (Fig. 1) after duplicate removal, consisted in filtering the collected literature corpus for publications focusing specifically on the removal of introduced fish from mountain lakes. To do so, we first annotated the papers with the full list of search terms and counted the frequency of each of these search terms in the title, abstract, and author keywords of each paper. To calculate an overall potential relevance score for each publication, which could be used for the ranking and prioritization of publications, these search terms frequencies were then multiplied by the terms’ specificity scores and a section weight, and values were summed up for each publication. The specificity scores attributed to each search term ranged from 1 (broad or general term such as lake or pond) to 3 (highly specific term related to fish removal such as rotenone or electrofishing) and are given in Supporting Information - Database 1. Search terms found in the abstract were assigned a weight of 1, whereas those found in the title or author keywords list were assigned a section weight of 5 because they were assumed to be more indicative of the primary focus of the publication. The annotated list was then sorted according to the publications’ potential relevance scores. However, because no score threshold could reliably distinguish relevant from irrelevant publications, all retrieved articles were subsequently screened by three of the study authors (DU, FS, SV), each of whom was assigned a subset of publications. This screening was performed based on the paper’s title and abstract and based on a series of exclusion criteria, but was conservative in that papers were retained if exclusion criteria could not be applied with sufficient certainty. Publications were excluded if they: (1) addressed introductions or stockings rather than removals, or (2) pertained to lotic instead of lentic ecosystems, or (3) described model-based studies, or (4) focused on unrelated topics (e.g., carcass decomposition, Allee effect, ecosystems or species outside the scope of the study), or (5) if the full text was not freely available (i.e., open access, through institutional or journal subscriptions, or direct requests to corresponding authors were unsuccessful). The four search results for which this was the case were either attributed to journals in Web of Science even though they were project descriptions or they had no digital object identifiers and were not traceable.

### Final Inclusion and Literature Analysis

For the analyses, the studies retained during the previous screening were randomly distributed among five of the study authors (DC, DU, FS, SV, TT) who read the full texts. Based on the full text, we excluded additional papers that provided no useful information on the removal method. The excluded papers typically mentioned the management of non-native fish species in mountain lakes in their titles and/or abstracts but did not specify in sufficient detail the removal method adopted in the full text.

The data extracted from each paper and used for further analyses included: study location (country, lake name, longitude, latitude), location characteristics (lake maximum depth, surface area, altitude), species information, introduction information (year and purpose of introduction), removal information (purpose of the intervention, removal method and duration, outcome, responsible stakeholder, and effects on the ecosystems). These data, organized by species and locations, are provided in Supporting Information—Database 2. When a paper provided information on one/more species from multiple locations, the data were introduced as separate entries (hereafter cases; i.e., unique combinations of single species and single locations) into the database. Because of the limited number of papers that were retained (see results), we performed only qualitative analyses.

We further compiled a trait database (Supporting Information—Database 3) relevant for the invasiveness potential of targeted species (excluding hybrids and subspecies), intended to support biologically-informed removal management approaches. This database includes taxonomic, life-history, ecological, and environmental traits, native and introduced ranges, habitat preferences, salinity and temperature tolerance, and fishing vulnerability. The trait information was compiled primarily from FishBase (Froese and Pauly 2026) and the IUCN Red List of Threatened Species (IUCN 2026), and complemented with data from authoritative invasive-species databases, governmental and institutional repositories, and peer-reviewed scientific literature (all references are provided within the database, for each species).

## Results

The first (naïve) and second (refined) searches returned 1278 and 1920 publications, respectively. After the removal of duplicates, the validation, and the final eligibility screening, 35 papers (n_p_) were retained. This corresponded to 184 cases (n_c_), i.e., unique combinations of single species and single locations (Supporting Information—Database 2).

### Temporal and Geographic Distribution

The 35 papers retained were published between 1968 and 2024 and dealt mostly with the management of fish introduced intentionally during the 20th century. Details regarding the introductions were mentioned in 31 papers. Of these, most reported that introductions were intentional (n_p _= 28), both intentional and accidental (n_p _= 2) or accidental (n_p _= 1). The purpose of intentional introductions was mentioned in 22 papers: for fishing (n_p _= 18), aquaculture (n_p _= 2), fishing and aquaculture (n_p _=  1), experimental (n_p _= 1), and biological control (i.e., predation; n_p_ = 1).

**Fig. 2:**
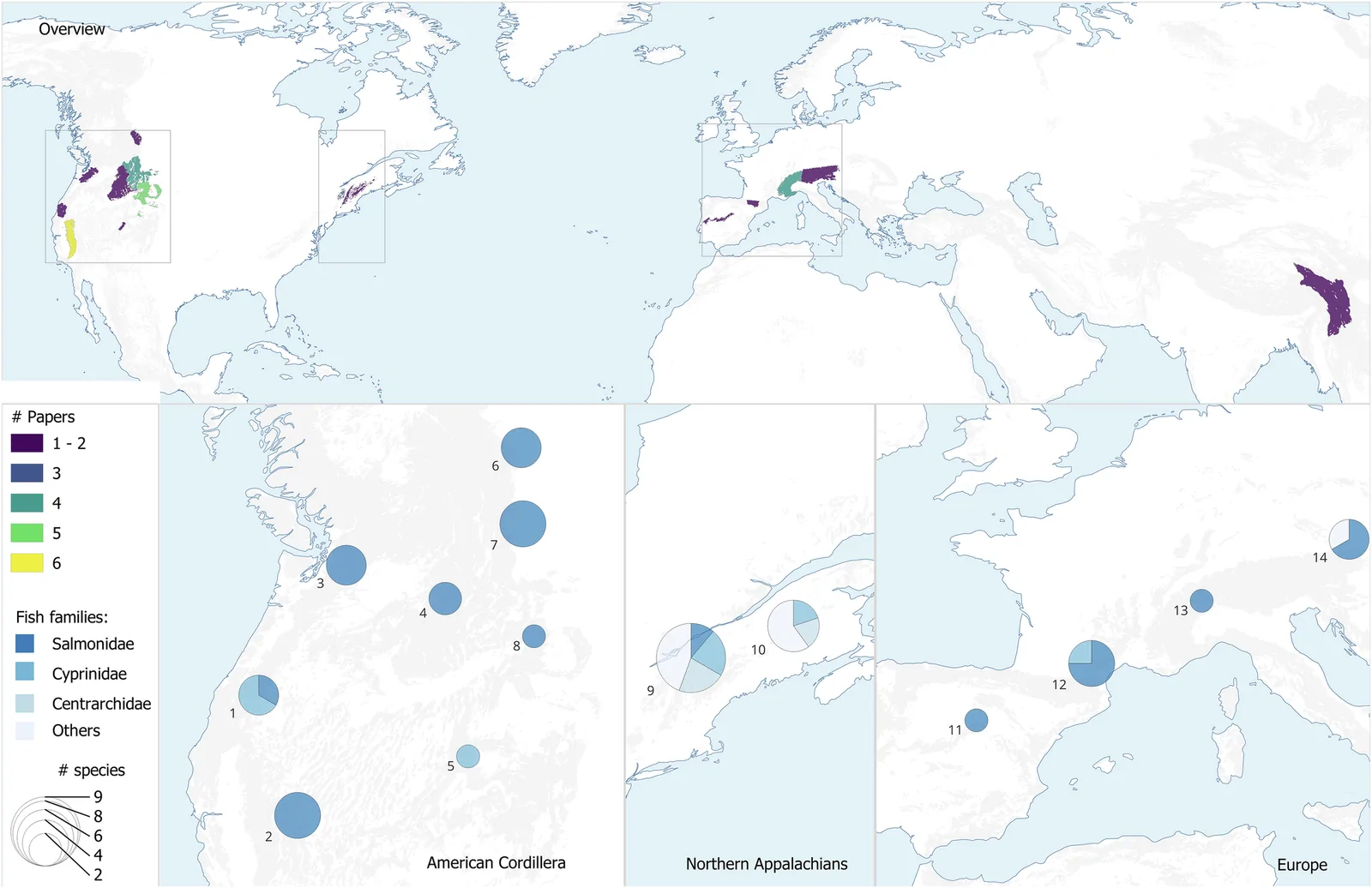
Spatial distribution and density of study sites (upper panel) and distribution of targeted species in the three most studied world regions (lower panels, also marked with bounding boxes in the upper panel). For the mapping, the 96 lakes, in which fish removal attempts were performed, are grouped in the following 15 mountain lake clusters: 1. Klamath Mountains / Southern Cascade Range; 2. Sierra Nevada; 3. Northern Cascade Range; 4. Idaho-Bitterroot Rocky Mountains; 5. Colorado Plateau; 6. Canadian Rockies; 7. Central Montana Rocky Mountains; 8. Greater Yellowstone Rockies; 9. Adirondacks; 10. Appalachians; 11. Spanish Central System; 12. Pyrenees; 13. Western Alps; 14. Eastern Alps; 15. Hengduan Shan (right most cluster in upper panel). The size of the circles represents the number of fish species targeted in each cluster of lakes, with the blue shades indicating their taxonomic family

The geographic distribution of the studies was strongly biased (Fig. 2), with 77% of the studies conducted in North America (the United States of America and Canada), 17% in Europe, and only one study in Asia (Yin et al. 2022). Within Canada, studies were concentrated in the province of Alberta only, Waterton Lakes National Park, and Banff National Park (Parker et al. 2001; Gray et al. 2014; Pacas and Taylor 2015; Beaulieu et al. 2021, 2024; Stuparyk and Vinebrooke 2024). In the United States of America, the majority of managed ecosystems were located in the West Coast states of Idaho, California, Montana, Wyoming, Washington, Utah, and Oregon, and only four on the East Coast states of New York and Maine (Foye 1968; Harig and Bain 1998; Knapp and Matthews 1998; Drake and Naiman 2000; Demong 2001; Hoffman et al. 2004; Sarnelle and Knapp 2004, 2005; Vredenburg 2004; Knapp et al. 2007; Pope et al. 2009; Syslo et al. 2011; Koenig et al. 2015; Winters et al. 2017; Fried et al. 2018; Taylor et al. 2020; Schnee et al. 2021; Siemiantkowski et al. 2022; Unsworth et al. 2022; Eilers et al. 2023).

In Europe, study locations were restricted to three sites in northern Italy (Tiberti et al. 2016, 2017, 2019, 2021), five sites in the Pyrenees (Miró et al. 2020; Toro et al. 2020; Tiberti et al. 2021), and two sites in central Austria (Schaufler et al. 2015; Schabetsberger et al. 2023). The 96 aquatic ecosystems mentioned across the 35 publications included lakes, ponds, reservoirs, and pools, ranging in elevation from 300 to 3600 m a.s.l., in size from 0.35 to 34,020 ha, and in maximum depth from 1.5 to 133 m (Supporting information—Database 2).

Removal campaigns were initiated as early as 1966 and conducted over varying time periods, ranging from one month to 11 years, in the case of a long-term fish-removal program in the USA. In the 20 papers reporting information on the stakeholders involved or in charge of the removal campaigns, responsibilities were primarily with government institutions alone (n_p _= 17) or in collaboration with researchers and volunteers (n_p _= 3).

### Species Targeted

**Fig. 3:**
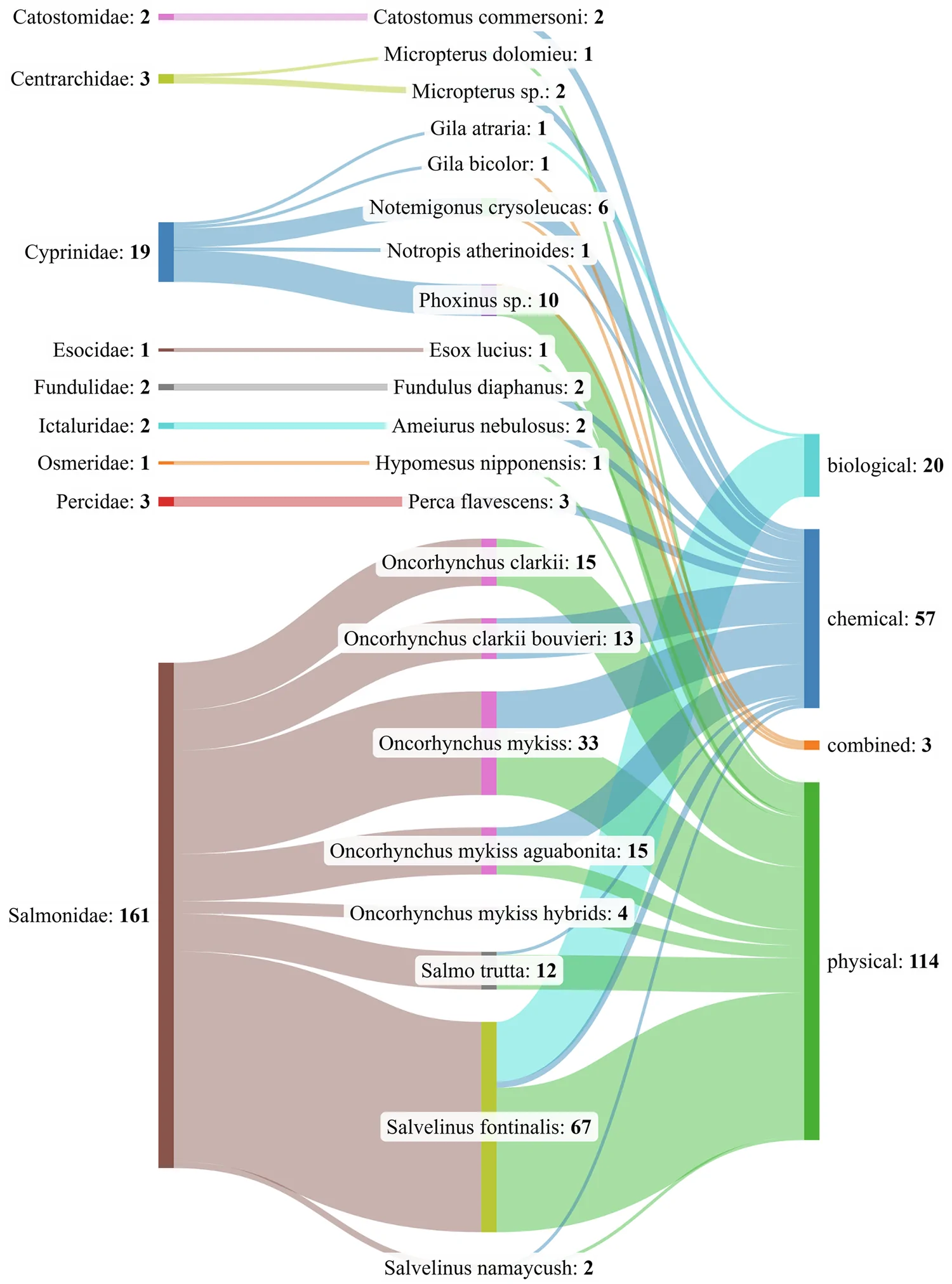
Distribution of cases (i.e., unique combinations of single species at single locations) across fish families (left) and removal approaches (right). Note that in one publication five cases pertained to three species in each location because outcomes were not listed individually for each species

The 35 publications discussed the removal of 21 introduced fish species (including subspecies and hybrids), grouped in nine families (Fig. 3). Salmonidae was the most prevalent family, the most targeted species being the brook trout (*Salvelinus fontinalis*, n_p_ = 21) and the rainbow trout (*Oncorhynchus mykiss*, including its hybrids with *O. clarkii*, and subspecies, n_p_ = 12). Cyprinidae represented the second most targeted group (n_p _= 8), with a majority of removal attempts performed on the golden shiner (*Notemigonus crysoleucas*, n_p_ = 4) and the minnow (*Phoxinus* sp., n_p_ = 3). The altitudinal distribution of removal sites varied between fish families: in the case of Salmonidae, target ecosystems ranged from approximately 350–3600 m a.s.l., whereas in the case of Cyprinidae and Osmeridae, no study site was above 2300 and 1000 m a.s.l., respectively. Removal efforts targeted only one species 60% of the time, and between two and five species otherwise (Supporting Information—Database 2).

All species targeted for removal were generalists, could tolerate wide temperature ranges, and presented diverse life-history trade-offs that enhance survival and population growth (e.g., short lifespan is compensated by high reproductive potential; low reproductive potential is compensated by early age of sexual maturity) (Supporting Information - Database 3).

### Removal Methods and Outcomes

Most studies (n_p _= 19) aimed at the recovery of native ecosystems and their native aquatic communities (fish, amphibians, and zooplankton). Within the remaining studies, the majority were methodological and assessed the efficiency of specific removal methods (n_p _= 12). Of the remaining four papers, three described the impact of fish removal on native species and ecosystems, and one described a study aimed at controlling parasite infestations caused by an introduced fish species.

Most studies implemented one removal approach, which was either physical (n_p _= 22, n_c_ = 104), chemical (n_p _= 8, n_c_ = 57), or biological (n_p_  = 3, n_c_ = 20) (Supporting Information–Fig. S1). Within the physical approach, gill netting and/or electrofishing were the most frequently-used methods. When chemicals were used, rotenone was favored (n_p _= 6) over other methods, such as antimycin, and organic matter for deoxygenation. Biological methods consisted in the introduction of genetically modified individuals or a predator. Combined approaches were adopted in only two papers (n_c _= 3) and used either physical and biological methods (i.e., shoreline trap nets together with predator introduction) or physical and chemical ones (i.e., shoreline trap nets; drainage, gill nets, baited traps, electrofishing, purse seines, liming together with rotenone).

Removal approaches varied between taxonomic groups but overall, only one approach was used for any taxonomic group, except for Salmonidae, Cyprinidae, and Centrarchidae, where two or more approaches were used (Fig. 3). The approach(es) adopted further varied depending on the objective of the study, although in most cases, at least two different categories of approaches were used for the same purpose (Supporting Information—Fig. S2). Within the segment of studies aiming at the recovery of native ecosystems and communities (n_p_ = 19), the approaches adopted further varied depending on what taxonomic group was to be recovered. Accordingly, physical approaches appeared to be favored when the goal was to recover amphibians and invertebrates, or the entire ecosystem. When the goal was to recover native fish populations, chemical and biological approaches were also adopted, with chemical methods being favored (Supporting Information—Fig. S3).

**Fig. 4:**
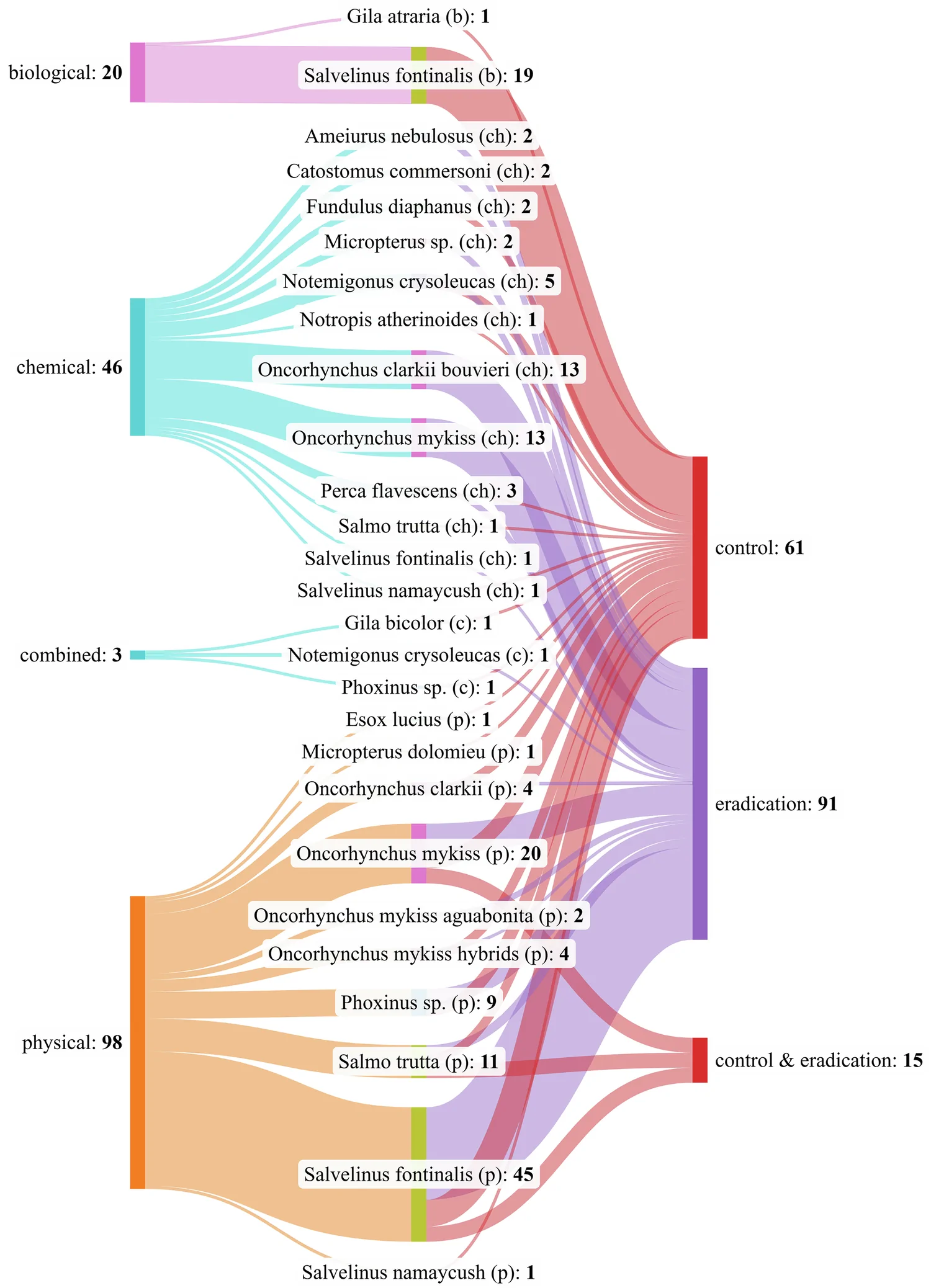
Distribution of cases (i.e., unique combinations of single species at single locations) across removal approaches (left) and their reported outcomes (right). Note that in one publication five cases pertained to three species in each location because outcomes were not listed individually for each species. b – biological, ch – chemical, c – combined, p – physical

The success of the removal actions (i.e., eradication, population reduction, both) was reported in 30 papers, corresponding to 157 cases. Removal efforts were fully successful (i.e., eradication) in more than half (58%) of the cases (Fig. 4). Within each management strategy, we found that the chemical approach was the most successful, with 80% of the corresponding cases leading to eradication.

The second most effective approach was the physical one, 60% of the cases reporting successful eradication. This trend (i.e., higher success of chemical methods) appears to be consistent across species, but generalizations cannot be made because of the taxonomic bias and limited sample size. In Salmonidae, the taxonomic group best represented in our review, chemical methods recorded the highest success (27 out of 29 cases resulted in eradication). Although the physical methods were more frequently used, they achieved a milder success rate by comparison (48 out of 77 cases resulted in eradication).

Among the successful chemical methods, rotenone was the most frequently-used agent, with few exceptions where antimycin was also involved. In the case of successful physical methods, gill netting alone was the most used, followed by combinations with electrofishing and fyke netting.

### FishME Toolbox

The FishME toolbox (https://mountainbiodiversity.org/projects/FishME/) is hosted within the GMBA Mountain Portal, which is a digital, open-access infrastructure for the collection, access, and sharing of data, information, and knowledge about our world’s mountain species and ecosystems as well as about the experts working on understanding, protecting, and managing them. By building on expert contributions and on powerful workflows to search and mine online resources, it brings together trusted biodiversity data, annotated knowledge, other reference data for analysis and visualization such as environmental layers, and validated information for any of the >8,000 mountain ranges of the latest global mountain inventory. The Portal is built on Wagtail backed by the Django Project designed for creating and managing dynamic websites. It leverages Server-Side Rendering (SSR) for faster responses and better Search Engine Optimization (SEO) and integrates PostgreSQL as the database backend. The Toolbox distinguishes between technical users, who interact with it to find domain-specific, management-relevant information, and non-technical users, who look for general-public information. Building on the relational database and concepts of linked data, the publications included in this work are linked both to the spatial information (i.e., study site location) they contain through an interactive map as well as search terms, and to the fish species they discuss. Additional information extracted from the publications are provided in the form of a downloadable excel database (Supporting Information – Database 2).

## Discussion

We contribute to a growing body of literature on the management of freshwater ecosystem invasions with a systematic synthesis of peer-reviewed literature on the removal of introduced fish species. Our work distinguishes itself from previous studies (e.g., Meronek et al. 1996; Rytwinski et al. 2019) in that it focuses solely on standing waters in mountain ecosystems.

The number of papers retained in our synthesis was low in comparison to the volume of literature on fish introductions and their consequences. Such a small corpus of literature logically reflects the narrow scope of our literature search, the strict delineation of mountains we applied (Snethlage et al. 2022), and the relative paucity of research in remote and hard to access mountain environments (e.g., Klein et al. 2019). However, it also reflects our decision to limit our search to peer-reviewed publications and thereby leave out the information sources to which the institutions, which we identified as most frequently in charge of removal campaigns (e.g., government institutions), contribute. The evidence base used for introduced fish management in mountain ecosystems can likely be considerably extended through the inclusion of gray literature (e.g., Carrillo et al. 2019), although accessibility and information extraction are typically more challenging. Finally, the hypothesis that projects have remained undocumented (Rytwinski et al. 2019), unpublished, or even confidential, not the least because interventions were unsuccessful, but also to avoid vandalism on eradication gear and restocking (Chiapella et al. 2018), cannot be excluded. This may further cause bias and confound information on the effectiveness of individual and species-specific methods in given contexts.

Our review points to both spatial and taxonomic biases and gaps in the available knowledge. The majority of studies (Foye 1968; Harig and Bain 1998; Knapp and Matthews 1998; Drake and Naiman 2000; Demong 2001; Parker et al. 2001; Hoffman et al. 2004; Sarnelle and Knapp 2004; Vredenburg 2004; Sarnelle and Knapp 2005; Knapp et al. 2007; Pope et al. 2009; Syslo et al. 2011; Gray et al. 2014; Koenig et al. 2015; Pacas and Taylor 2015; Winters et al. 2017; Fried et al. 2018; Taylor et al. 2020; Beaulieu et al. 2021; Schnee et al. 2021; Siemiantkowski et al. 2022; Unsworth et al. 2022; Eilers et al. 2023; Beaulieu et al. 2024; Stuparyk and Vinebrooke 2024) were conducted in North America (Canada and USA), which is in line with previous findings (Rytwinski et al. 2019; Bernery et al. 2022; Haubrock et al. 2025). This bias might reflect the higher number of introduced freshwater fish reported for North America compared to other world regions (Sultana and Hashim 2015), possibly limiting knowledge transfer to other regions. Evidence from Europe (Schaufler et al. 2015; Tiberti et al. 2016, 2017, 2019; Miró et al. 2020; Toro et al. 2020; Tiberti et al. 2021; Schabetsberger et al. 2023) comes primarily from removal efforts carried out in the context of initiatives such as the Life Resque AlPyr and LIFE+ LimnoPirineus projects, which aimed to restore aquatic ecosystems and species in high mountain areas of the Italian Alps and the Pyrenees (Carrillo et al. 2019; Ventura et al. 2022). Unlike in other fields of mountain biodiversity research (e.g., Praeg et al. 2025), evidence from Asia was very limited (Yin et al. 2022).

The increasing number of introduced freshwater fish species and their spread across continents and ecosystems (Sultana and Hashim 2015) call for addressing the geographic bias we detected by scaling up efforts of documenting their presence and removal worldwide. However, the difficulties associated with biodiversity research and management in mountain ecosystems, including the steepness and remoteness of the terrain (see Klein et al. 2019), are often prohibitive and call for the development and adoption of novel technologies such as airborne or satellite sensors, including autonomous aerial vehicles, for monitoring non-native species and assessing the response of both biotic and abiotic variables to management decisions over time (see Randin et al. 2020 and references therein). These technologies also represent powerful means to collect the ecological data needed to inform methodological choices, spatio-temporal planning, and adaptive management. Such increased documentation efforts further ought to be accompanied by a fundamental change in publication requirements and formats such as to facilitate the dissemination of and inclusive access to data and information on successes as well as failures across scales and locations. Online platforms such as the FishME Toolbox represent dynamic and user-friendly options for knowledge dissemination that effectively complement written formats.

Our review shows that removal efforts were focused on a limited number of species. The most targeted ones, which are also the most frequently introduced in mountain lakes worldwide, were the rainbow trout (*Oncorhynchus* spp.) and the brook trout (*Salvelinus fontinalis*). Both are “legend invaders” (Sultana and Hashim 2015; Bernery et al. 2022) that propagate in every continent due to their effective life-history strategies as well as phenotypic, physiological, and behavioral characteristics that allow their survival and establishment across diverse, often harsh environmental conditions.

Knowledge of species biological traits (e.g., water depth preference, specific physiological capacities, life history plasticity), the ecological parameters of invaded systems (e.g., water turbidity, pH), and introduction pathways (e.g., Bernery et al. 2024) is critical for understanding the invasion process and planning successful management approaches (see Pou-Rovira et al. 2019; Rytwinski et al. 2019; Bernery et al. 2022, 2024; Lambert et al. 2023). This calls for the systematic assessment and integration of such knowledge into the planning process, to guide the selection of approaches and methods most likely to lead to the desired outcomes, allow adjusting the efforts to effectively target specific individuals and/or life stages (e.g., Dunlop et al. 2018; Tiberti et al. 2021), and hopefully improve management success rates beyond the approximately 50% we and others (e.g., Meronek et al. 1996; Rytwinski et al. 2019) estimated.

We are aware that time and accessibility constraints, as well as ecosystem complexity (Eilers et al. 2023), are only a few of the numerous factors that limit the collection of the necessary field data in mountain regions. Accordingly, key information and long-term datasets on the target species (e.g., size and life-stage distributions, age of sexual maturation, habitat preferences) and their habitats (e.g., size, altitude, aquatic vegetation distribution, and native species diversity) are often missing, despite their critical importance to conservation practitioners (e.g., Armstrong and Knapp 2004; Pou-Rovira et al. 2019; Koel et al. 2020). Such an information gap undermines the robust planning and execution of conservation efforts (Britton et al. 2011), and the evaluation of their effectiveness. This is of high relevance especially in the case of unselective removal methods, such as chemical treatments, applied in sensitive ecosystems that shelter species of conservation concern (e.g., Beaulieu et al. 2021, 2024 and references therein).

In the absence of field data for many species in the majority of places, information from trait databases such as the one we compiled and model-based approaches using past removal data can provide valuable guidance and insights to conservation practitioners. These can include modeling population size and ecosystem changes (e.g., Koel et al. 2020), assessing invasion potential and the effectiveness of specific management interventions, or simulating various adaptive management strategies over time, including uncertainties and associated costs (see e.g., Dominguez Almela et al. 2021; Diallo et al. 2025).

The majority of the papers analyzed in this review reported the application of physical removal methods, mainly gill netting and electrofishing. Possible reasons for the selection of such methods despite the overall higher efficacy of chemical ones (see e.g., Brown 2010), are multiple. First, chemical methods are not species-specific and would remove both native and introduced fish species. They are also toxic to other non-target organisms, like amphibians. Although both physical and chemical methods are largely non-selective, the latter has considerably more negative side effects (see e.g., Ling 2003; Brown 2010), even when included in baits (Saari 2023). As such, chemical methods are often not an advisable option, in particular in the presence of species at risk or in the case of sensitive habitats. Second, in the European Union, the use of rotenone has been banned since 2008 (European Commission Decision no. 317/2008) and Antimycin-A is not approved (i.e., under Regulation (EC) No 1907/2006). In the United States, although the use of rotenone is permitted, it is subject to strict guidelines and criminal penalties may apply in case of violations (Finlayson 2023). In such a context, physical removal approaches appear attractive as they do not require similar types of special permits and come at no comparable risk of law encroachment. Finally, gears and techniques used for the physical removal of fish are similar if not identical to those that fisheries managers use for routine monitoring and management (Brown 2010).

Past removal actions documented in our study showed objective-based method selection. Thus, physical methods were exclusively used when the objective was the recovery of amphibians or invertebrates, and in most cases for whole ecosystem recovery. Biological methods were almost exclusively used when aiming at ecosystem recovery. However, when the recovery of native fish was the main objective, chemical methods were almost exclusively used. In this latter case, the indiscriminate eradication of fish (both native and non-native) was most likely considered acceptable since the ecosystem could be later restocked with native fish species. Ecosystem surface, depth, complexity, and connectivity, as well as the recovery capacity of the native communities, might contribute to the selection and efficiency of removal approaches.

Fish removal initiatives need to make use of the growing body of data available and model the possible outputs based on the time-frame and resources available. Learning from past removal data is especially useful for optimizing methods and allocation of removal efforts and resources (Hansen et al. 2016; Koel et al. 2020; Yoğurtçuoğlu et al. 2021; Diallo et al. 2025). Assessing the performance of previous studies prior to removal attempts, the critical evaluation of objectives and assumptions (e.g., Klein et al. 2023) as well as long-term pre- and post-intervention monitoring (e.g., Meronek et al. 1996; Donaldson and Cooke 2016) are likely to become even more relevant as invasions are increasing and climate change affects both biotic interactions (Koel et al. 2020) and abiotic conditions (e.g., Vilizzi et al. 2021).

Irrespective of the existence of efficiency data, cost-benefit estimations (Donaldson and Cooke 2016) are difficult to perform and evaluate because of the recurrent lack of cost information. In Norway, rotenone treatments were estimated to cost between 10,000 and more than 200,000 euros per year depending on the species and site characteristics, with most costs associated with the work hours (Bardal 2019). Equipment costs likely remain generally comparatively low with estimated costs for motorized electrofishing equipment, for instance, ranging between approximately 6000 and 20,000 euros depending on the power, and fishing nets prices varying between >100 to around 1000 euros per unit depending on the type, size requirements, country, and supplier (expert personal communications). In the mountain context, where study sites are often remote and difficult to access, helicopter flights might represent additional high expenses (e.g., Pacas and Taylor 2015).

The relevance of economic considerations on the choice of optimal management strategies (e.g., Buhle et al. 2005) calls for more precise cost estimations and reporting. However, it also calls for exploring options to optimize investments and bolster benefits, opportunities, and efficiencies. Means to do so include collaborative approaches among multiple landowners, stakeholders, and interest groups (e.g., Buktenica et al. 2018), watershed or landscape approaches that can help the prioritization of management targets, and transdisciplinary approaches that recognize mountain lakes as social-ecological systems (see Chiapella et al. 2018). In the case of mountain lakes, which have particularly high aesthetic and recreational value (e.g., Schirpke et al. 2021; Ebner 2024), societal support in favor of fish eradication might be both particularly important but also challenging to gain as wilderness fishing in mountains has garnered high cultural value (Chiapella et al. 2018). Gaining a better understanding of public perceptions towards mountain lakes and public knowledge about the ecological consequences of fish introductions might help improve crucial communication and awareness raising campaigns and gain support towards management interventions (Gozlan 2008; Bennett et al. 2017; Chiapella et al. 2018).

Our literature search focused on fish removal under the assumption that eradication of introduced species is the most desirable outcome, even though examples exist in which eradication had detrimental effects on the natural ecosystems (Eisendle et al. 2022). However, even when desired, eradication might occasionally be too expensive or unlikely because of population densities and/or lake size. In such cases, suppression strategies appear as useful alternatives to achieve positive management outcomes, such as increases in native fish abundance (e.g., Weidel et al. 2007). Under close monitoring, population reduction below the threshold causing negative effects (functional eradication) might also appear sufficient (see Diallo et al. 2025). Scaling up the management of introduced fish species in mountain ecosystems, especially when resources are limited and remoteness is prohibitive, might require moving away from strict eradication goals and aiming for mitigation rather than remediation (Gozlan et al. 2010). This in turn implies risk management assessments and decisions (Gozlan et al. 2010), the adoption of different management targets, and adaptive management strategies (e.g., Eisendle et al. 2022; Eilers et al. 2023) on a case-by-case basis. Early detection through routine surveillance (e.g., using eDNA as an early detection tool) associated with rapid and targeted interventions have a great potential to help circumvent the challenges associated with delayed measures and enable successful eradication (Gozlan et al. 2010; Sabas et al. 2026).

## Conclusion

Fish introductions are widespread in mountains but peer-reviewed literature on their management in lentic ecosystems is limited and biased. In the absence of robust evidence to inform optimal allocation of removal efforts and method selection, in the face of strong push back from individual stakeholder groups, and given the high costs and uncertainties associated with restoration measures in mountains, preventing unwanted fish introductions as well as early detection through surveillance remain essential. Prevention and very early, rapid, and efficient interventions appear particularly important in the case of mountain freshwater ecosystems that host species with unique adaptations to harsh environment conditions as well as endemic or rare species, many of which already face increasing risks of local or global extinction due to global change. However, the fact that fish introductions are on the rise calls for concerted efforts to fill the knowledge gaps that our synthesis reveals. Despite these gaps, our work provides a useful knowledge base that we make accessible in support of rising awareness and informed management in our online FishME toolbox.

## Supplementary information

Supporting Information – Figures

Supporting Information – Tables

Supporting Information – Database 1

Supporting Information – Database 2

Supporting Information – Database 3

## Data Availability

All data supporting the findings of this study are available within the paper and its Supplementary Information files.
