# Supplementary material for: Introduced Fish in Mountains: State of Published Research on their Removal from Lentic Ecosystems
Source: Environ Manage. 2026 Sep 9;76(9):304. doi: 10.1007/s00267-026-02616-9 (PMC13558331; doi:10.1007/s00267-026-02616-9)
Supplement: Supplementary file 1 [file 267_2026_2616_MOESM1_ESM.docx]

**Supporting Information - Figures**

**Introduced fish in mountains: state of published research on their removal from lentic ecosystems**

Dan Cogălniceanu^1^, Florina Stănescu^1,2,*^, Teodora L. Tănase^1,3^, Sabina E. Vlad^1,2,3^, Mark Snethlage^4,5^, Ajay Ranipeta^6^, Davnah Urbach^4,5^

^1^Ovidius University of Constanţa, Research Center of the Natural Sciences Department, Constanţa, Romania

^2^Ovidius University of Constanţa, Center for Morphological and Genetic Studies of Malignant Pathology (CEDMOG), Constanţa, Romania

^3^Asociația Chelonia România, Bucharest, Romania

^4^Global Mountain Biodiversity Assessment, University of Bern, Institute for Plant Sciences, Bern, Switzerland

^5^Centre Interdisciplinaire de Recherche sur la Montagne, University of Lausanne, Lausanne, Switzerland

^6^Binomal LLC, New Haven, Connecticut, United States of America

*Corresponding author: Florina Stănescu; e-mail: [florina.stanescu@365.univ-ovidius.ro](mailto:florina.stanescu@365.univ-ovidius.ro)

**Fig. S1** Distribution of cases (i.e. unique combinations of single species at single locations) across the fish removal approaches (i.e., biological, chemical, physical or combined; left) indicating if single or multiple methods were used, and the methods applied (right) in the revised literature on fish removal from mountain standing waters.

**Fig. S2** Fish removal approaches, according to the aim of the study (number of cases is mentioned between parentheses).

**Fig. S3** Fish removal approaches according to the type of native communities targeted for recovery. The scale represents the number of cases.
