# Supplementary material for: Introduced Fish in Mountains: State of Published Research on their Removal from Lentic Ecosystems
Source: Environ Manage. 2026 Sep 9;76(9):304. doi: 10.1007/s00267-026-02616-9 (PMC13558331; doi:10.1007/s00267-026-02616-9)
Supplement: Supplementary file 2 [file 267_2026_2616_MOESM2_ESM.docx]

**Supporting Information - Tables**

**Introduced fish in mountains: state of published research on their removal from lentic ecosystems**

Dan Cogălniceanu^1^, Florina Stănescu^1,2,*^, Teodora L. Tănase^1,3^, Sabina E. Vlad^1,2,3^, Mark Snethlage^4,5^, Ajay Ranipeta^6^, Davnah Urbach^4,5^

^1^Ovidius University of Constanţa, Research Center of the Natural Sciences Department, Constanţa, Romania

^2^Ovidius University of Constanţa, Center for Morphological and Genetic Studies of Malignant Pathology (CEDMOG), Constanţa, Romania

^3^Asociația Chelonia România, Bucharest, Romania

^4^Global Mountain Biodiversity Assessment, University of Bern, Institute for Plant Sciences, Bern, Switzerland

^5^Centre Interdisciplinaire de Recherche sur la Montagne, University of Lausanne, Lausanne, Switzerland

^6^Binomal LLC, New Haven, Connecticut, United States of America

*Corresponding author: Florina Stănescu; e-mail: [florina.stanescu@365.univ-ovidius.ro](mailto:florina.stanescu@365.univ-ovidius.ro)

**Table S1**. List of search terms used for the naïve search.

| **Category** | **Search string** |
| --- | --- |
| Ecosystem | River, permanent pond, temporary pond, lake, mire, bog, aquatic ecosystems, lentic, lotic, marsh, marshland, swamp, sump, wetland, stream, reservoir, loch, freshwater, high mountain lake, brook, mountain reservoir, aquatic habitats, water bodies |
| Management category | Removal, extraction, eradication, elimination, cull, control, conservation, restoration, recovery, extinction, introduction, extirpation, prevention, containment |
| Method | Mechanical, biological, chemical, genetic, nets, traps, electrofishing, rotenone, ichthyocides, piscicides, drain, draining, biological control, fishing, gillnet |
| Fish species | Salmonid, cypriniform, cychlids, poecilid, centrarchid, ictalurid, clariid, trout, tilapias, catfish, minnow, char, perch, *Salmo trutta*, *Oncorhynchus mykiss*, *Salvelinus fontinalis*, *Salvelinus alpinus*, *Phoxinus phoxinus*, *Phoxinus* sp.*, Perca fluviatili* |
| Other | Non-native, introduced, recreation, fishing, invasive, alien, pest, alpine, mountain, fishless, top predator, stocking, angling |

Table S2. Search string used for the refined search

(TI=(lake$ OR lagoon$ OR pond$ OR pool$ OR reservoir$ OR loch OR kettle OR cauldron)

OR

AB=(lake$ OR lagoon$ OR pond$ OR pool$ OR reservoir$ OR loch OR kettle OR cauldron)

OR

AK=(lake$ OR lagoon$ OR pond$ OR pool$ OR reservoir$ OR loch OR kettle OR cauldron))

AND

(TI=(actinopterygii OR astyanax OR brycon OR cottus OR cyprinodon* OR fundulus OR garra OR carassius OR catostomus OR couesius OR ctenopharyngodon OR gambusia OR lepomis OR micropterus OR notemigonus OR oncorhynchus OR perca OR percichthys OR pimephales OR pseudorasbora OR pungitius OR rhinichthys OR richardsonius OR rutilus OR salmo OR salvelinus OR siphateles OR tinca OR characiform* OR cyprinodontiformes OR esociformes OR "osmeriform*" OR perciform* OR salmoniform* OR siluriform* OR "esox americanus" OR "esox lucius" OR "hypomesus transpacificus" OR "ictalurus punctatus" OR "micropterus salmoides" OR "osmerus eperlanus" OR "rutilus rutilus" OR "silurus glanis" OR catfish OR centrarchid* OR char OR clariid* OR cychlid$ OR cypriniform* OR fish* OR fishes OR ictalurid* OR ictalurus OR minnow$ OR "oncorhynchus mykiss" OR "perca fluviatilis" OR perch OR "phoxinus phoxinus" OR "phoxinus sp." OR poeciliid* OR "salmo trutta " OR salmonid* OR "salvelinus alpinus" OR "salvelinus fontinalis" OR "rainbow trout" OR "brook trout" OR "goldfish" OR "carassius auratus" OR "catostomus tahoensis" OR "couesius plumbeus" OR "lake chub" OR "ctenopharyngodon idella" OR "gambusia affinis" OR "lepomis cyanellus" OR "lepomis gibbosus" OR "lepomis macrochirus" OR "micropterus dolomieu" OR "micropterus salmoides" OR "notemigonus crysoleucas" OR "oncorhynchus aguabonita" OR "golden trout" OR "oncorhynchus clarkii" OR "yellowstone cutthroat" OR "perca flavescens" OR "percichthys colhuapiensis" OR "percichthys trucha" OR "pimephales promelas" OR "pseudorasbora parva" OR "pungitius pungitius" OR "rhinichthys osculus" OR "richardsonius egregious" OR "rutilus rutilus" OR "salmo salar" OR "salvelinus namaycush" OR "lake trout" OR "siphateles bicolor" OR "tinca tinca" OR tilapia$ OR trout$ OR Ichthyolog* OR Ichthyofauna)

OR

AB=(actinopterygii OR astyanax OR brycon OR cottus OR cyprinodon* OR fundulus OR garra OR carassius OR catostomus OR couesius OR ctenopharyngodon OR gambusia OR lepomis OR micropterus OR notemigonus OR oncorhynchus OR perca OR percichthys OR pimephales OR pseudorasbora OR pungitius OR rhinichthys OR richardsonius OR rutilus OR salmo OR salvelinus OR siphateles OR tinca OR characiform* OR cyprinodontiformes OR esociformes OR "osmeriform*" OR perciform* OR salmoniform* OR siluriform* OR "esox americanus" OR "esox lucius" OR "hypomesus transpacificus" OR "ictalurus punctatus" OR "micropterus salmoides" OR "osmerus eperlanus" OR "rutilus rutilus" OR "silurus glanis" OR catfish OR centrarchid* OR char OR clariid* OR cychlid$ OR cypriniform* OR fish* OR fishes OR ictalurid* OR ictalurus OR minnow$ OR "oncorhynchus mykiss" OR "perca fluviatilis" OR perch OR "phoxinus phoxinus" OR "phoxinus sp." OR poeciliid* OR "salmo trutta " OR salmonid* OR "salvelinus alpinus" OR "salvelinus fontinalis" OR "rainbow trout" OR "brook trout" OR "goldfish" OR "carassius auratus" OR "catostomus tahoensis" OR "couesius plumbeus" OR "lake chub" OR "ctenopharyngodon idella" OR "gambusia affinis" OR "lepomis cyanellus" OR "lepomis gibbosus" OR "lepomis macrochirus" OR "micropterus dolomieu" OR "micropterus salmoides" OR "notemigonus crysoleucas" OR "oncorhynchus aguabonita" OR "golden trout" OR "oncorhynchus clarkii" OR "yellowstone cutthroat" OR "perca flavescens" OR "percichthys colhuapiensis" OR "percichthys trucha" OR "pimephales promelas" OR "pseudorasbora parva" OR "pungitius pungitius" OR "rhinichthys osculus" OR "richardsonius egregious" OR "rutilus rutilus" OR "salmo salar" OR "salvelinus namaycush" OR "lake trout" OR "siphateles bicolor" OR "tinca tinca" OR tilapia$ OR trout$ OR Ichthyolog* OR Ichthyofauna)

OR

AK=(actinopterygii OR astyanax OR brycon OR cottus OR cyprinodon* OR fundulus OR garra OR carassius OR catostomus OR couesius OR ctenopharyngodon OR gambusia OR lepomis OR micropterus OR notemigonus OR oncorhynchus OR perca OR percichthys OR pimephales OR pseudorasbora OR pungitius OR rhinichthys OR richardsonius OR rutilus OR salmo OR salvelinus OR siphateles OR tinca OR characiform* OR cyprinodontiformes OR esociformes OR "osmeriform*" OR perciform* OR salmoniform* OR siluriform* OR "esox americanus" OR "esox lucius" OR "hypomesus transpacificus" OR "ictalurus punctatus" OR "micropterus salmoides" OR "osmerus eperlanus" OR "rutilus rutilus" OR "silurus glanis" OR catfish OR centrarchid* OR char OR clariid* OR cychlid$ OR cypriniform* OR fish* OR fishes OR ictalurid* OR ictalurus OR minnow$ OR "oncorhynchus mykiss" OR "perca fluviatilis" OR perch OR "phoxinus phoxinus" OR "phoxinus sp." OR poeciliid* OR "salmo trutta " OR salmonid* OR "salvelinus alpinus" OR "salvelinus fontinalis" OR "rainbow trout" OR "brook trout" OR "goldfish" OR "carassius auratus" OR "catostomus tahoensis" OR "couesius plumbeus" OR "lake chub" OR "ctenopharyngodon idella" OR "gambusia affinis" OR "lepomis cyanellus" OR "lepomis gibbosus" OR "lepomis macrochirus" OR "micropterus dolomieu" OR "micropterus salmoides" OR "notemigonus crysoleucas" OR "oncorhynchus aguabonita" OR "golden trout" OR "oncorhynchus clarkii" OR "yellowstone cutthroat" OR "perca flavescens" OR "percichthys colhuapiensis" OR "percichthys trucha" OR "pimephales promelas" OR "pseudorasbora parva" OR "pungitius pungitius" OR "rhinichthys osculus" OR "richardsonius egregious" OR "rutilus rutilus" OR "salmo salar" OR "salvelinus namaycush" OR "lake trout" OR "siphateles bicolor" OR "tinca tinca" OR tilapia$ OR trout$ OR Ichthyolog* OR Ichthyofauna) )

AND

(TI=(invasive OR invasion OR alien OR "fish introduction" OR "introduced fish" OR nonnative OR "non native" OR "non-indigenous" OR "undesired fish" OR exotic OR "fish stocking" OR "injurious fish" OR "noxious fish" OR "nuisance fish" OR "unwanted fish" OR "feral fish" OR "fish release*" OR "releas* fish" OR "containment" OR "population control" OR "integrated pest management" OR "pest management" OR "contain fish population" OR "control fish population$" OR "control fish population$" OR "control fish" OR "control alien fish" OR "control of alien fish" OR "control invasive fish" OR "control of invasive fish" OR "control exotic fish" OR "control of exotic fish" OR "control trout population" OR "reduc* fish population" OR "reduc* invasive fish population" OR "reduc* alien fish population" OR "reduc* trout population$" OR "fish containment" OR "control populations of invasive fish" OR "fish population containment" OR "fish population reduction" OR "fish population control" OR "fish control" OR "control populations of invasive fish" OR "population containment" OR "population reduction" OR "population control" OR cull* OR eliminat* OR eradicat* OR extirpat* OR extract* OR kill OR killing OR remov* OR "cull trout" OR "cull* fish" OR "cull* invasive fish" OR "cull* non native fish" OR "cull* nonnative fish" OR "cull* of non native fish" OR "cull* of nonnative fish" OR "cull* alien fish" OR "cull* of alien fish" OR "cull* exotic fish" OR "cull* of exotic fish" OR "kill* invasive fish" OR "kill* of invasive fish" OR "kill* alien fish" OR "kill* of alien fish" OR "kill* nonnative fish" OR "kill* of nonnative fish" OR "kill* non native fish" OR "kill* of non native fish" OR "kill* exotic fish" OR "kill* of exotic fish" OR "cull* minnow$" OR "eliminat* fish" OR "eliminat* of fish" OR "eliminat* alien fish" OR "eliminat* of alien fish" OR "eliminat* invasive fish" OR "eliminat* of invasive fish" OR "eliminat* exotic fish" OR "eliminat* of exotic fish" OR "eliminat* nonnative fish" OR "eliminat* non native fish" OR "eliminat* of nonnative fish" OR "eliminat* of non native fish" OR "eliminat* trout" OR "eradicat* fish" OR "eradicat* of fish" OR "eradicat* alien fish" OR "eradicat* of alien fish" OR "eradicat* nonnative fish" OR "eradicat* non native fish" OR "eradicat* of nonnative fish" OR "eradicat* of non native fish" OR "eradicat* invasive fish" OR "eradicat* of invasive fish" OR "eradicat* exotic fish" OR "eradicat* of exotic fish" OR "eradicat* minnow$" OR "fish population containment" OR "kill fish" OR "killing fish" OR "minnow cull" OR "minnow eradication" OR "minnow removal" OR "remov* fish" OR "remov* of fish" OR "remov* alien fish" OR "remov* of alien fish" OR "remov* invasive fish" OR "remov* of invasive fish" OR "remov* nonnative fish" OR "remov* non native fish" OR "remov* of nonnative fish" OR "remov* of non native fish" OR "remov* exotic fish" OR "remov* of exotic fish" OR "remov* minnow$" OR "remov* trout" OR "suppress* fish" OR "suppress* invasive fish" OR "suppress* alien fish" OR "suppress* nonnative fish" OR "suppress* non native fish" OR "suppress* of nonnative fish" OR "suppress* of non native fish" OR "fish eliminat*" OR "fish extirpat*" OR "fish remov*" OR "fish cull*" OR "fish eradicat*" OR "fish kill*" OR "fish suppress*" OR "trout population control" OR "trout population reduction" OR "trout elimination" OR "induce high mortality" OR suppress* OR "trout cull" OR "invader suppression" OR "invader eradication" OR "invader elimination" OR "adaptive management" OR "integrated pest management" OR "biological control" OR "biological eradication" OR "cyprinid herpesvirus" OR "judas fish" OR "judas technique" OR "parasite based control" OR "pathogen based control" OR "pathogen release$" OR "pheromone technique$" OR "predator introduction" OR "predator release" OR "predator based control" OR "sterile male release" OR "sterile male technique" OR "tiger muskellunge$" OR "biological control agent*" OR "breeding cycle disruption" OR "chemical sterilization" OR "chromosomal technolog*" OR "decomposing carcass*" OR "fish based organic pellet$" OR "genetic modification" OR "genetic technolog*" OR "habitat modification$" OR "introduction of natural predator$" OR "organic pellet$" OR "organic pellet decomposition" OR "organic soy pellet$" OR "organic wheat pellet$" OR "pelletized carcass analog$" OR "pest$ release" OR "plant based organic pellet$" OR "predatory species introduction" OR "radiative sterilization" OR sterilization OR sterilisation OR biocide$ OR "chemical method$" OR "fish poison$" OR ichthyocide$ OR piscicide$ OR rotenone OR antimycin OR "chemical eradication" OR "chemical reclamation" OR "copper sulfate" OR "nonsynergized CFT Legumine rotenone" OR noxfish OR nusyn OR "osmotic shock" OR poison$ OR "reduce DO below lethal levels" OR "sodium cyanide" OR "electrofish*" OR "electro fish*" OR "three pass depletion" OR "three pass electrofishing removal$" OR "three pass remova$l" OR "electrofishing removal" OR "electrofishing eliminat*" OR "electrofishing suppress*" OR "electrofishing cull*" OR "electrofishing remov*" OR "electrofishing eradicat*" OR "electrofishing kill*" OR electrofishing OR "environmental modification" OR "autocidal technolog*" OR "female biased sex ratio distortion" OR "gene silencing" OR "genetic engineering" OR "genetic manipulation" OR "genetic technology*" OR "genetic control" OR "immuno contraception" OR "m yy eradication" OR "male sterilization" OR "parasitically vectored immune contraception" OR "pheromone$ based" OR "recombinant genetics" OR "repressible male sterili*" OR "rna guided gene drive" OR semiochemical OR "sterile feral technolog*" OR "sterile triploid" OR sterilization OR sterilisation OR "transgene technique$" OR triploidy OR "trojan y chromosome strategy" OR "virally vectored immune contraception" OR "immune contraception" OR "yy male stocking" OR "yy male" OR "adult removal" OR barrier$ OR draining OR drain$ OR "fish ladder$" OR "fish weir$" OR fishing OR "gill net*" OR "hand net*" OR "hook and line" OR "manual suppression" OR "mechanical method" OR "net$" OR netting OR "physical removal*" OR "push net*" OR "removal fisher*" OR "seine net*" OR seining OR "throw net*" OR "trammel net*" OR trap$ OR "trawl net*" OR "mechanical eradication" OR "mechanical elimination" OR dewatering OR "direct capture" OR "fish net*" OR fishnet* OR "fish trap*" OR "fyke net*" OR "manual method$" OR screen$ OR "water level management")

OR

AB=(invasive OR invasion OR alien OR "fish introduction" OR "introduced fish" OR nonnative OR "non native" OR "non-indigenous" OR "undesired fish" OR exotic OR "fish stocking" OR "injurious fish" OR "noxious fish" OR "nuisance fish" OR "unwanted fish" OR "feral fish" OR "fish release*" OR "releas* fish" OR "containment" OR "population control" OR "integrated pest management" OR "pest management" OR "contain fish population" OR "control fish population$" OR "control fish population$" OR "control fish" OR "control alien fish" OR "control of alien fish" OR "control invasive fish" OR "control of invasive fish" OR "control exotic fish" OR "control of exotic fish" OR "control trout population" OR "reduc* fish population" OR "reduc* invasive fish population" OR "reduc* alien fish population" OR "reduc* trout population$" OR "fish containment" OR "control populations of invasive fish" OR "fish population containment" OR "fish population reduction" OR "fish population control" OR "fish control" OR "control populations of invasive fish" OR "population containment" OR "population reduction" OR "population control" OR cull* OR eliminat* OR eradicat* OR extirpat* OR extract* OR kill OR killing OR remov* OR "cull trout" OR "cull* fish" OR "cull* invasive fish" OR "cull* non native fish" OR "cull* nonnative fish" OR "cull* of non native fish" OR "cull* of nonnative fish" OR "cull* alien fish" OR "cull* of alien fish" OR "cull* exotic fish" OR "cull* of exotic fish" OR "kill* invasive fish" OR "kill* of invasive fish" OR "kill* alien fish" OR "kill* of alien fish" OR "kill* nonnative fish" OR "kill* of nonnative fish" OR "kill* non native fish" OR "kill* of non native fish" OR "kill* exotic fish" OR "kill* of exotic fish" OR "cull* minnow$" OR "eliminat* fish" OR "eliminat* of fish" OR "eliminat* alien fish" OR "eliminat* of alien fish" OR "eliminat* invasive fish" OR "eliminat* of invasive fish" OR "eliminat* exotic fish" OR "eliminat* of exotic fish" OR "eliminat* nonnative fish" OR "eliminat* non native fish" OR "eliminat* of nonnative fish" OR "eliminat* of non native fish" OR "eliminat* trout" OR "eradicat* fish" OR "eradicat* of fish" OR "eradicat* alien fish" OR "eradicat* of alien fish" OR "eradicat* nonnative fish" OR "eradicat* non native fish" OR "eradicat* of nonnative fish" OR "eradicat* of non native fish" OR "eradicat* invasive fish" OR "eradicat* of invasive fish" OR "eradicat* exotic fish" OR "eradicat* of exotic fish" OR "eradicat* minnow$" OR "fish population containment" OR "kill fish" OR "killing fish" OR "minnow cull" OR "minnow eradication" OR "minnow removal" OR "remov* fish" OR "remov* of fish" OR "remov* alien fish" OR "remov* of alien fish" OR "remov* invasive fish" OR "remov* of invasive fish" OR "remov* nonnative fish" OR "remov* non native fish" OR "remov* of nonnative fish" OR "remov* of non native fish" OR "remov* exotic fish" OR "remov* of exotic fish" OR "remov* minnow$" OR "remov* trout" OR "suppress* fish" OR "suppress* invasive fish" OR "suppress* alien fish" OR "suppress* nonnative fish" OR "suppress* non native fish" OR "suppress* of nonnative fish" OR "suppress* of non native fish" OR "fish eliminat*" OR "fish extirpat*" OR "fish remov*" OR "fish cull*" OR "fish eradicat*" OR "fish kill*" OR "fish suppress*" OR "trout population control" OR "trout population reduction" OR "trout elimination" OR "induce high mortality" OR suppress* OR "trout cull" OR "invader suppression" OR "invader eradication" OR "invader elimination" OR "adaptive management" OR "integrated pest management" OR "biological control" OR "biological eradication" OR "cyprinid herpesvirus" OR "judas fish" OR "judas technique" OR "parasite based control" OR "pathogen based control" OR "pathogen release$" OR "pheromone technique$" OR "predator introduction" OR "predator release" OR "predator based control" OR "sterile male release" OR "sterile male technique" OR "tiger muskellunge$" OR "biological control agent*" OR "breeding cycle disruption" OR "chemical sterilization" OR "chromosomal technolog*" OR "decomposing carcass*" OR "fish based organic pellet$" OR "genetic modification" OR "genetic technolog*" OR "habitat modification$" OR "introduction of natural predator$" OR "organic pellet$" OR "organic pellet decomposition" OR "organic soy pellet$" OR "organic wheat pellet$" OR "pelletized carcass analog$" OR "pest$ release" OR "plant based organic pellet$" OR "predatory species introduction" OR "radiative sterilization" OR sterilization OR sterilisation OR biocide$ OR "chemical method$" OR "fish poison$" OR ichthyocide$ OR piscicide$ OR rotenone OR antimycin OR "chemical eradication" OR "chemical reclamation" OR "copper sulfate" OR "nonsynergized CFT Legumine rotenone" OR noxfish OR nusyn OR "osmotic shock" OR poison$ OR "reduce DO below lethal levels" OR "sodium cyanide" OR "electrofish*" OR "electro fish*" OR "three pass depletion" OR "three pass electrofishing removal$" OR "three pass remova$l" OR "electrofishing removal" OR "electrofishing eliminat*" OR "electrofishing suppress*" OR "electrofishing cull*" OR "electrofishing remov*" OR "electrofishing eradicat*" OR "electrofishing kill*" OR electrofishing OR "environmental modification" OR "autocidal technolog*" OR "female biased sex ratio distortion" OR "gene silencing" OR "genetic engineering" OR "genetic manipulation" OR "genetic technology*" OR "genetic control" OR "immuno contraception" OR "m yy eradication" OR "male sterilization" OR "parasitically vectored immune contraception" OR "pheromone$ based" OR "recombinant genetics" OR "repressible male sterili*" OR "rna guided gene drive" OR semiochemical OR "sterile feral technolog*" OR "sterile triploid" OR sterilization OR sterilisation OR "transgene technique$" OR triploidy OR "trojan y chromosome strategy" OR "virally vectored immune contraception" OR "immune contraception" OR "yy male stocking" OR "yy male" OR "adult removal" OR barrier$ OR draining OR drain$ OR "fish ladder$" OR "fish weir$" OR fishing OR "gill net*" OR "hand net*" OR "hook and line" OR "manual suppression" OR "mechanical method" OR "net$" OR netting OR "physical removal*" OR "push net*" OR "removal fisher*" OR "seine net*" OR seining OR "throw net*" OR "trammel net*" OR trap$ OR "trawl net*" OR "mechanical eradication" OR "mechanical elimination" OR dewatering OR "direct capture" OR "fish net*" OR fishnet* OR "fish trap*" OR "fyke net*" OR "manual method$" OR screen$ OR "water level management")

OR

AK=(invasive OR invasion OR alien OR "fish introduction" OR "introduced fish" OR nonnative OR "non native" OR "non-indigenous" OR "undesired fish" OR exotic OR "fish stocking" OR "injurious fish" OR "noxious fish" OR "nuisance fish" OR "unwanted fish" OR "feral fish" OR "fish release*" OR "releas* fish" OR "containment" OR "population control" OR "integrated pest management" OR "pest management" OR "contain fish population" OR "control fish population$" OR "control fish population$" OR "control fish" OR "control alien fish" OR "control of alien fish" OR "control invasive fish" OR "control of invasive fish" OR "control exotic fish" OR "control of exotic fish" OR "control trout population" OR "reduc* fish population" OR "reduc* invasive fish population" OR "reduc* alien fish population" OR "reduc* trout population$" OR "fish containment" OR "control populations of invasive fish" OR "fish population containment" OR "fish population reduction" OR "fish population control" OR "fish control" OR "control populations of invasive fish" OR "population containment" OR "population reduction" OR "population control" OR cull* OR eliminat* OR eradicat* OR extirpat* OR extract* OR kill OR killing OR remov* OR "cull trout" OR "cull* fish" OR "cull* invasive fish" OR "cull* non native fish" OR "cull* nonnative fish" OR "cull* of non native fish" OR "cull* of nonnative fish" OR "cull* alien fish" OR "cull* of alien fish" OR "cull* exotic fish" OR "cull* of exotic fish" OR "kill* invasive fish" OR "kill* of invasive fish" OR "kill* alien fish" OR "kill* of alien fish" OR "kill* nonnative fish" OR "kill* of nonnative fish" OR "kill* non native fish" OR "kill* of non native fish" OR "kill* exotic fish" OR "kill* of exotic fish" OR "cull* minnow$" OR "eliminat* fish" OR "eliminat* of fish" OR "eliminat* alien fish" OR "eliminat* of alien fish" OR "eliminat* invasive fish" OR "eliminat* of invasive fish" OR "eliminat* exotic fish" OR "eliminat* of exotic fish" OR "eliminat* nonnative fish" OR "eliminat* non native fish" OR "eliminat* of nonnative fish" OR "eliminat* of non native fish" OR "eliminat* trout" OR "eradicat* fish" OR "eradicat* of fish" OR "eradicat* alien fish" OR "eradicat* of alien fish" OR "eradicat* nonnative fish" OR "eradicat* non native fish" OR "eradicat* of nonnative fish" OR "eradicat* of non native fish" OR "eradicat* invasive fish" OR "eradicat* of invasive fish" OR "eradicat* exotic fish" OR "eradicat* of exotic fish" OR "eradicat* minnow$" OR "fish population containment" OR "kill fish" OR "killing fish" OR "minnow cull" OR "minnow eradication" OR "minnow removal" OR "remov* fish" OR "remov* of fish" OR "remov* alien fish" OR "remov* of alien fish" OR "remov* invasive fish" OR "remov* of invasive fish" OR "remov* nonnative fish" OR "remov* non native fish" OR "remov* of nonnative fish" OR "remov* of non native fish" OR "remov* exotic fish" OR "remov* of exotic fish" OR "remov* minnow$" OR "remov* trout" OR "suppress* fish" OR "suppress* invasive fish" OR "suppress* alien fish" OR "suppress* nonnative fish" OR "suppress* non native fish" OR "suppress* of nonnative fish" OR "suppress* of non native fish" OR "fish eliminat*" OR "fish extirpat*" OR "fish remov*" OR "fish cull*" OR "fish eradicat*" OR "fish kill*" OR "fish suppress*" OR "trout population control" OR "trout population reduction" OR "trout elimination" OR "induce high mortality" OR suppress* OR "trout cull" OR "invader suppression" OR "invader eradication" OR "invader elimination" OR "adaptive management" OR "integrated pest management" OR "biological control" OR "biological eradication" OR "cyprinid herpesvirus" OR "judas fish" OR "judas technique" OR "parasite based control" OR "pathogen based control" OR "pathogen release$" OR "pheromone technique$" OR "predator introduction" OR "predator release" OR "predator based control" OR "sterile male release" OR "sterile male technique" OR "tiger muskellunge$" OR "biological control agent*" OR "breeding cycle disruption" OR "chemical sterilization" OR "chromosomal technolog*" OR "decomposing carcass*" OR "fish based organic pellet$" OR "genetic modification" OR "genetic technolog*" OR "habitat modification$" OR "introduction of natural predator$" OR "organic pellet$" OR "organic pellet decomposition" OR "organic soy pellet$" OR "organic wheat pellet$" OR "pelletized carcass analog$" OR "pest$ release" OR "plant based organic pellet$" OR "predatory species introduction" OR "radiative sterilization" OR sterilization OR sterilisation OR biocide$ OR "chemical method$" OR "fish poison$" OR ichthyocide$ OR piscicide$ OR rotenone OR antimycin OR "chemical eradication" OR "chemical reclamation" OR "copper sulfate" OR "nonsynergized CFT Legumine rotenone" OR noxfish OR nusyn OR "osmotic shock" OR poison$ OR "reduce DO below lethal levels" OR "sodium cyanide" OR "electrofish*" OR "electro fish*" OR "three pass depletion" OR "three pass electrofishing removal$" OR "three pass remova$l" OR "electrofishing removal" OR "electrofishing eliminat*" OR "electrofishing suppress*" OR "electrofishing cull*" OR "electrofishing remov*" OR "electrofishing eradicat*" OR "electrofishing kill*" OR electrofishing OR "environmental modification" OR "autocidal technolog*" OR "female biased sex ratio distortion" OR "gene silencing" OR "genetic engineering" OR "genetic manipulation" OR "genetic technology*" OR "genetic control" OR "immuno contraception" OR "m yy eradication" OR "male sterilization" OR "parasitically vectored immune contraception" OR "pheromone$ based" OR "recombinant genetics" OR "repressible male sterili*" OR "rna guided gene drive" OR semiochemical OR "sterile feral technolog*" OR "sterile triploid" OR sterilization OR sterilisation OR "transgene technique$" OR triploidy OR "trojan y chromosome strategy" OR "virally vectored immune contraception" OR "immune contraception" OR "yy male stocking" OR "yy male" OR "adult removal" OR barrier$ OR draining OR drain$ OR "fish ladder$" OR "fish weir$" OR fishing OR "gill net*" OR "hand net*" OR "hook and line" OR "manual suppression" OR "mechanical method" OR "net$" OR netting OR "physical removal*" OR "push net*" OR "removal fisher*" OR "seine net*" OR seining OR "throw net*" OR "trammel net*" OR trap$ OR "trawl net*" OR "mechanical eradication" OR "mechanical elimination" OR dewatering OR "direct capture" OR "fish net*" OR fishnet* OR "fish trap*" OR "fyke net*" OR "manual method$" OR screen$ OR "water level management"))

AND

(TI=(alpine OR subalpine OR montane OR submontane OR mountain OR mountainous OR upland OR highland OR "high* elevation" OR "high* altitude" OR summit OR yunga OR puna OR paramo OR glacial OR himalayan OR carpathian OR caucasian OR appalachian OR cordilleran OR pyrenean OR tibetan OR cantabrian OR altiplano OR afromontane OR afroalpine OR madrean OR paramo OR yunga OR puna OR upland OR "eastern arc" OR "mid* elevation" OR "mid* altitude" OR cantabrian OR pontic OR glacial OR summit OR volcanic OR crater OR paramo$ OR páramo$ OR subkryal OR "sub kryal" OR tarn OR "glacial pond$" OR "glacier fed aquatic" OR "glacier fed catchment$" OR "glacier fed channel$" OR "glacier fed pond$" OR "glacier fed spring$" OR "glacier fed water$" OR "thaw pond$" OR "mountain torrent$" OR "altitud* decline" OR "altitud* decrease" OR "altitud* distribution$" OR "altitud* diversit*" OR "altitud* gradient$" OR "altitud* increase" OR "altitud* limit$" OR "altitud* pattern$" OR "altitud* range$" OR "altitud* shift$" OR "altitud* transect$" OR "altitud* variabilit*" OR "altitud* variation$" OR "altitud* zonation$" OR "campo* rupestre$" OR "cryo oromediterranean" OR "elevation* decline" OR "elevation* decrease" OR "elevation* distribution$" OR "elevation* diversit*" OR "elevation* gradient$" OR "elevation* increase" OR "elevation* limit$" OR "elevation* pattern$" OR "elevation* range$" OR "elevation* shift$" OR "elevation* transect$" OR "elevation* variabilit*" OR "elevation* variation$" OR "elevation* zonation$" OR "glacial retreat$" OR "gradient$ of altitude" OR "gradient$ of elevation" OR "hautes terres" OR "high* altitude$" OR "high* elevation$" OR "rock glacier$" OR "sky island$" OR "tropical glacier$" OR afroalpine OR "afro alpine" OR afromontane OR "afro montane" OR alpine OR alpino$ OR altiplano$ OR banjaran OR cordilheira$ OR cordill?re$ OR cordillera$ OR daglar OR daglari OR fjell OR fjellkjede OR gebirge OR gunung OR highland$ OR khrebet OR montane OR mountain$ OR mountainous OR mountaintop$ OR nunatak$ OR pegunungan OR planina OR pohorie OR serra$ OR serralada$ OR sierra$ OR stratovolcano* OR "strato volcano*" OR subalpine OR submontane OR "sub montane" OR upland$ OR volcano* OR "glacier outflow$" OR glaciolacustrine OR "glacio lacustrine" OR submountainous OR "sub mountainous" OR "żywiec beskids" OR "žumberak mts" OR "zomba plateau" OR "zillertal alps" OR "zarafshan range" OR "zanskar range" OR "zane hills" OR "zagros mts" OR "yunnan-guizhou plateau" OR "yungui plateau" OR "yukon-tanana uplands" OR "yu shan" OR "yorkshire dales" OR "yi shan" OR "yenisei ridge" OR "yenisei range" OR "yellowstone plateau" OR "yangming shan" OR "xue shan" OR "xiong'er shan" OR "xiao shan" OR "xiao hinggan ling" OR "x hills" OR "wyoming range" OR "wudang shan" OR "wooley range" OR "wind river range" OR "willapa hills" OR "wild horse range" OR "wieliczka foothills" OR "wichita mts" OR "whitefish range" OR "white river plateau" OR "white range" OR "white pine range" OR "white mts" OR "white divide" OR "white carpathians" OR "whistler range" OR "western venezuelan andes" OR "western tian shan" OR "western taurus" OR "western tatras" OR "western tatra*" OR "western tannu ola" OR "western sudetes" OR "western sayan" OR "western sahara atlas" OR "western romanian carpathians" OR "western rhodopes" OR "western range" OR "western pyrenees" OR "western pamirs" OR "western pacific islands" OR "western middle atlas" OR "western main range" OR "western lesser himalaya*" OR "western kunlun" OR "western hindu kush" OR "western himalaya*" OR "western high atlas" OR "western hellenides" OR "western hajar" OR "western ghat" OR "western ghats" OR "western foothills" OR "western caucasus" OR "western carpathians" OR "western bieszczady" OR "western betics" OR "western beskids" OR "western balkan mts" OR "western anti-atlas" OR "western alps" OR "west* carpathian*" OR "west* african uplands" OR "west usambara" OR "west range" OR "west kunlun" OR "west coast ranges" OR "west coast range" OR "wenatchee ridge" OR "wellington range" OR "weepah hills" OR "webster range" OR "wassuk range" OR "washakie range" OR "wasatch range" OR "walker range" OR "walden ridge" OR "waitakere ranges" OR "waianae range" OR "vosges mts" OR "voras mts" OR "volga uplands" OR "volcanic hills" OR "volcanes de colima" OR "vitim plateau" OR "virginia range" OR "villány mts" OR "victorian alps" OR "victoria range" OR "vértes hills" OR "vermilion range" OR "verkhoyansk ridge" OR "verkhoyansk range" OR "vercors massif" OR "venezuelan coastal range" OR "venezuelan andes" OR "venetian prealps" OR "velka fatra" OR "velika kapela" OR "velence hills" OR "vanoise massif" OR "valais alps" OR "val di non" OR "usun apau plateau" OR "usambara mts" OR "ural mts" OR "upper atlas" OR "united states range" OR "umbrian apennines" OR "ulugh muztagh" OR "ulagan plateau" OR "ulaan taiga" OR "ukrainian carpathians" OR "uinkaret plateau" OR "udokan ridge" OR "udokan range" OR "tyan shan" OR "two thumb range" OR "twin range" OR "tuscan–emilian apennines" OR "turkestan range" OR "tunka ridge" OR "tunisian dorsale" OR "tunisian atlas" OR "tunguska plateau" OR "tukuringra ridge" OR "tukuringra range" OR "tug hill" OR "tsingy de bemaraha" OR "tsambagarav ridge" OR "truong son range" OR "truckee range" OR "tropical andes" OR "trinity range" OR "trinity alps" OR "trialeti range" OR "trara* mount*" OR "transylvanian plateau" OR "transylvanian plain" OR "transverse ranges" OR "trans-mexican volcanic belt" OR "trans-ili alatau" OR "tosco romagnolo apennines" OR "torlesse range" OR "toquima range" OR "tongbai shan" OR "tomkinson ranges" OR "tokaj mts" OR "toiyabe range" OR "tobin range" OR "tobacco range" OR "toano range" OR "timpahute range" OR "tigray plateau" OR "tien shan" OR "tibetan plateau" OR "tianmu shan" OR "tian shan" OR "thuringian forest" OR "thompson peaks" OR "thompson peak" OR "thomas range" OR "thirtymile range" OR "the sperrins" OR "the snowies" OR "the maniototo" OR "the hunter's hills" OR "the hokonuis" OR "the catlins" OR "the ancient wall" OR "teton range" OR "tethyan orogenic belt" OR "terskey ala-too" OR "termit massif" OR "terminal range" OR "tenmile range" OR "temblor range" OR "tell atlas" OR "telangana plateau" OR "te urewera" OR "tchabal mbabo" OR "tawau hills" OR "taurus range" OR "taurus mts" OR "tatras mts" OR "tassili n'ajjer" OR "taringatura hills" OR "tararua range" OR "tannu ola" OR "tanggula mts" OR "talkeetna mts" OR "talas alatau" OR "taita hills" OR "taillefer massif" OR "taihang shan" OR "tai shan" OR "tadrart acacus" OR "système ibérique" OR "synnyr massif" OR "swiss prealps" OR "swiss jura" OR "swiss alps" OR "świętokrzyskie mts" OR "sweetgrass hills" OR "swannell ranges" OR "swan range" OR "swabian jura" OR "suva planina" OR "surrey hills" OR "superior upland" OR "suntar-khayata ridge" OR "suntar-khayata range" OR "summit ridge" OR "sumava national park" OR "sulphur spring range" OR "suisse normande" OR "submeseta sur" OR "submeseta norte" OR "sublette range" OR "sublett range" OR "stubai alps" OR "stuart range" OR "strzelecki ranges" OR "strathbogie ranges" OR "storm ridge" OR "stolowe mts" OR "stirling range" OR "stillwater range" OR "stikine plateau" OR "stauning alps" OR "stara planina" OR "stanovoy range" OR "stanovik range" OR "stanford range" OR "sredna gora" OR "sredinny range" OR "spotted range" OR "spectrum range" OR "specter range" OR "spatsizi plateau" OR "spanish pyrenees" OR "spanish peaks" OR "southwestern pyrenees" OR "southwestern massif central" OR "southwestern alps" OR "southland hills" OR "southern zagros" OR "southern wind river range" OR "southern western ghats" OR "southern wasatch range" OR "southern urals" OR "southern ural*" OR "southern uplands" OR "southern schwarzwald" OR "southern scandes" OR "southern sawatch range" OR "southern rockies" OR "southern rhodopes" OR "southern range" OR "southern peaks" OR "southern patagonian andes" OR "southern oregon coast range" OR "southern oregon cascades" OR "southern new hampshire" OR "southern limestone alps" OR "southern japanese alps" OR "southern great escarpment" OR "southern cumberland plateau" OR "southern catskills" OR "southern carpathians" OR "southern california coast ranges" OR "southern calcareous alps" OR "southern black hills" OR "southern black forest" OR "southern bakony" OR "southern aravalli range" OR "southern appalachians" OR "southern appalachian ridges" OR "southern apennines" OR "southern andes" OR "southern alps" OR "southern african ranges" OR "southern adirondacks" OR "southeastern pyrenees" OR "southeastern massif central" OR "southeastern alps" OR "south western caucasus" OR "south western alps" OR "south pennines" OR "south pare" OR "south pahroc range" OR "south eastern caucasus" OR "south eastern alps" OR "south downs" OR "south altai" OR "soufrière hills" OR "sonoma range" OR "song shan" OR "somali plateau" OR "solution range" OR "solitude range" OR "snowy range" OR "snowcrest range" OR "snake river range" OR "snake range" OR "slovenian alps" OR "slovak paradise" OR "slovak karst" OR "slovak carpathians" OR "slate range" OR "slate mts" OR "skopska crna gora" OR "skippers range" OR "siwalik range" OR "sitheri hills" OR "sistema periférico" OR "sistema mediterráneo catalán" OR "sistema ibérico" OR "sistema de tandilia" OR "sistema central" OR "sistema bético" OR "sirumalai hills" OR "sirente velino" OR "singatse range" OR "simpson park range" OR "simi hills" OR "silvretta massif" OR "silver peaks range" OR "silver peak range" OR "silesian foothills" OR "silesian beskids" OR "sila piccola" OR "sila greca" OR "sila grande" OR "sikkim himalayas" OR "sifton ranges" OR "shoshone range" OR "shiwan dashan" OR "shivwits plateau" OR "shimba hills" OR "sheep range" OR "sheep creek range" OR "shan hills" OR "shaler range" OR "seymour range" OR "sevier plateau" OR "sette daban ridge" OR "sette daban range" OR "setesdal vesthei" OR "setesdal ryfylke" OR "servarayan hills" OR "serres de llevant" OR "serranías chiquitanas" OR "serranía del litoral" OR "serranía del interior" OR "serranía del aguaragüe" OR "serrania de ronda" OR "serranía de pirre" OR "serranía de macuira" OR "serranía de los yariguíes" OR "serranía de los paraguas" OR "serranía de los churumbelos" OR "serranía de las quinchas" OR "serranía de cuenca" OR "serranía de chiribiquete" OR "serra parima" OR "serra negra" OR "serra mariola" OR "serra jeci" OR "serra grande" OR "serra geral" OR "serra geral de goiás" OR "serra gaúcha" OR "serra dourada" OR "serra dos tapes" OR "serra dos pirineus" OR "serra dos órgãos" OR "serra dos cristais" OR "serra dos cocais" OR "serra dos cavalos" OR "serra dos carajás" OR "serra dos aimorés" OR "serra do xistral" OR "serra do tombador" OR "serra do tabuleiro" OR "serra do suído" OR "serra do sincorá" OR "serra do rio do rastro" OR "serra do ramalho" OR "serra do papagaio" OR "serra do moco" OR "serra do marão" OR "serra do mar" OR "serra do lajeado" OR "serra do gandarela" OR "serra do estrondo" OR "serra do espinhaço" OR "serra do espigão" OR "serra do divisor" OR "serra do courel" OR "serra do cipó" OR "serra do caparaó" OR "serra do caldeirão" OR "serra do cachimbo" OR "serra do cabral" OR "serra d'irta" OR "serra d'espadà" OR "serra del montsant" OR "serra del catllaràs" OR "serra del cadí" OR "serra de uruburetama" OR "serra de são miguel" OR "serra de são mamede" OR "serra de santana" OR "serra de sant gervàs" OR "serra de paranapiacaba" OR "serra de montemuro" OR "serra de montejunto" OR "serra de monchique" OR "serra de maracaju" OR "serra de les gavarres" OR "serra de joão do vale" OR "serra de gerês" OR "serra de botucatu" OR "serra de baturité" OR "serra das matas" OR "serra das confusões" OR "serra das araras" OR "serra da saudade" OR "serra da peneda" OR "serra da nogueira" OR "serra da neve" OR "serra da mocidade" OR "serra da meruoca" OR "serra da mantiqueira" OR "serra da malcata" OR "serra da leba" OR "serra da jacobina" OR "serra da graciosa" OR "serra da estrela" OR "serra da capivara" OR "serra da capelada" OR "serra da cantareira" OR "serra da canastra" OR "serra da borda" OR "serra da bodoquena" OR "serra da bocaina" OR "serra da aratanha" OR "serra calderona" OR "serra azul" OR "serbian carpathians" OR "sentinel range" OR "sentinel hills" OR "selwyn range" OR "selennyakh ridge" OR "selawik hills" OR "selamiut range" OR "seaward kaikoura range" OR "seaman range" OR "scotchman peak*" OR "schrader range" OR "schell creek range" OR "scenic rim" OR "sayan mts" OR "sawtooth ridge" OR "sawtooth range" OR "sawatch range" OR "saur ridge" OR "satpura range" OR "sarntal alps" OR "sarek mts" OR "santa lucia range" OR "sandwich range" OR "sandhills range" OR "sand springs range" OR "san rafael swell" OR "san juan mts" OR "san francisco peaks" OR "salt river range" OR "salt range" OR "salair ridge" OR "sacramento mts" OR "sabana de bogotá" OR "russian caucasus" OR "russian altai" OR "rumpi hills" OR "rudnyi altai" OR "ruby range" OR "ruahine range" OR "royston hills" OR "royal group" OR "rough ridge" OR "romanian carpathians" OR "rolwaling himal" OR "rocky range" OR "rocky mts" OR "rock and pillar range" OR "robinson ranges" OR "robertson range" OR "roan plateau" OR "roan cliffs" OR "river mts" OR "ritter range" OR "rimutaka range" OR "rila mts" OR "rila geopark" OR "richmond range" OR "richmond hills" OR "rhodope mts" OR "rhenish massif" OR "rewa plateau" OR "reveille range" OR "retezat mts" OR "resting spring range" OR "redcloud peak" OR "red sea hills" OR "red hills" OR "raukumara range" OR "rattlesnake hills" OR "raton mesas" OR "range of the shelter" OR "rampart range" OR "ram range" OR "rakwana hills" OR "rakhine yoma" OR "rakaposhi range" OR "rajmahal hills" OR "raja ampat islands" OR "rainbow range" OR "ragged range" OR "quinn canyon range" OR "queen elizabeth range" OR "quantock hills" OR "qinling dabashan" OR "qingzang gaoyuan" OR "qinghai nanshan" OR "qin ling" OR "qilian mts" OR "qian shan" OR "qaidam basin" OR "pyrenees range" OR "putu range" OR "putorana plateau" OR "purcell range" OR "puna de atacama" OR "puertos de beceite" OR "provence alps" OR "price range" OR "presidential range" OR "president range" OR "prealpi giulie" OR "prealpi bergamasche" OR "préalpes de nice" OR "požeška gora" OR "považský inovec" OR "pouakai range" OR "portneuf range" OR "porcupine hills" OR "polonyna borzhava" OR "polish carpathians" OR "polar urals" OR "polar ural*" OR "polar range" OR "pohronský inovec" OR "pocono plateau" OR "po hills" OR "pliny range" OR "plateau of tibet" OR "plateau of mozambique" OR "plateau de millevaches" OR "plateau de danyi" OR "planalto da borborema" OR "planalto brasileiro" OR "piute range" OR "piton des neiges" OR "piton de la fournaise" OR "pisa range" OR "pir panjal range" OR "pioneer range" OR "pintler range" OR "pine grove hills" OR "pine forest range" OR "pilot range" OR "pilot butte" OR "pikikiruna range" OR "picos de europa" OR "pico island" OR "pico da neblina" OR "petermann ranges" OR "peruvian andes" OR "persian plateau" OR "pennine alps" OR "peninsular ranges" OR "peñas de cervera" OR "peloritani range" OR "pekulney range" OR "pegu yoma" OR "pavant range" OR "patagonian andes" OR "park range" OR "paria plateau" OR "pare mts" OR "páramos de la lora" OR "páramo de sumapaz" OR "páramo de pisba" OR "páramo de cruz verde" OR "paradise range" OR "paparoa range" OR "pangong range" OR "pancake range" OR "panamint range" OR "pan ling" OR "pampa del tamarugal" OR "pamir mts" OR "palatinate forest" OR "palani hills" OR "palamalai hills" OR "pakistan himalaya" OR "pahute mesa" OR "pahranagat range" OR "pah rah range" OR "paeroa range" OR "pacific ranges" OR "pacific coast ranges" OR "pachaimalai hills" OR "pab range" OR "ozark plateau" OR "ozark foothills" OR "owen stanley range" OR "outer western carpathians" OR "outer eastern carpathians" OR "ougarta range" OR "ouarsenis range" OR "ouarsenis massif" OR "ötztal alps" OR "otway ranges" OR "otago hills" OR "orulgan ridge" OR "orulgan range" OR "ortler alps" OR "oregon coast range" OR "oregon cascades" OR "ore mts" OR "ordos plateau" OR "orange river valley" OR "old man range" OR "oku massif" OR "okanogan range" OR "obudu plateau" OR "oban hills" OR "nyika plateau" OR "nyenchen tanglha" OR "nyambene hills" OR "nyainqentanglha shan" OR "nuratau range" OR "nun kun" OR "nulato hills" OR "noyon uul" OR "northwestern pyrenees" OR "northwestern caucasus" OR "northwestern altai" OR "northwestern alps" OR "northwest adirondacks" OR "northern zagros" OR "northern wind river range" OR "northern western ghats" OR "northern wasatch range" OR "northern virginia blue ridge" OR "northern urals" OR "northern tyrolean limestone alps" OR "northern tablelands" OR "northern schwarzwald" OR "northern scandes" OR "northern sawatch range" OR "northern sangre de cristo range" OR "northern rockies" OR "northern ranges" OR "northern range" OR "northern pindus" OR "northern patagonian andes" OR "northern oregon coast range" OR "northern madison range" OR "northern karakorum" OR "northern greater khingan" OR "northern front ranges" OR "northern front range" OR "northern cumberland plateau" OR "northern california coast ranges" OR "northern blue ridge" OR "northern black hills" OR "northern black forest" OR "northern bitterroot range" OR "northern bakony" OR "northern appalachians" OR "northern apennines" OR "northern andes" OR "northern altai" OR "northern alps" OR "northern allegheny plateau" OR "northern adirondacks" OR "northern absaroka range" OR "northeastern pyrenees" OR "northeastern massif central" OR "northeastern dolomites" OR "northeastern altai" OR "northeastern alps" OR "north york moors" OR "north western alps" OR "north range" OR "north pennines" OR "north patagonian massif" OR "north pare" OR "north island volcanic plateau" OR "north eastern caucasus" OR "north eastern alps" OR "north central zagros" OR "north cascades" OR "north canterbury hills" OR "norra storfjället" OR "nopah range" OR "nizke tatry" OR "niut range" OR "nisling range" OR "nimba range" OR "nightcap range" OR "nicholas range" OR "ngong hills" OR "newton ridge" OR "newton range" OR "new zealand uplands" OR "new pass range" OR "new england uplands" OR "new england upland" OR "nepal himalaya" OR "néouvielle massif" OR "needle range" OR "nanling mts" OR "nanga parbat" OR "nandi hills" OR "nan shan" OR "nan ling" OR "namuli massif" OR "namanga hills" OR "nallamala hills" OR "nakhon si thammarat range" OR "nakai plateau" OR "naga hills" OR "myjava hills" OR "muzkol range" OR "musgrave ranges" OR "murchison range" OR "muránska planina" OR "muntanyes de prades" OR "mummy range" OR "mt trumbull" OR "mt thielsen" OR "mt shasta" OR "mt rogers" OR "mt robson" OR "mt rainier" OR "mt princeton" OR "mt olympus" OR "mt marcy" OR "mt kenya" OR "mt jefferson" OR "mt hood" OR "mt erciyes" OR "mt elgon" OR "mt daniel" OR "mt cameroon" OR "mt baldy" OR "mt ararat" OR "mt adams" OR "mount yale" OR "mount whitney" OR "mount washington" OR "mount trumbull" OR "mount tongariro" OR "mount tianmu" OR "mount thielsen" OR "mount tambora" OR "mount tai" OR "mount st* helens" OR "mount spokane" OR "mount shasta" OR "mount saint helens" OR "mount sabalan" OR "mount rundle" OR "mount ruapehu" OR "mount rogers" OR "mount robson" OR "mount rinjani" OR "mount rainier" OR "mount princeton" OR "mount pilchuck" OR "mount paiko" OR "mount ossa" OR "mount olympus" OR "mount nemrut" OR "mount namuli" OR "mount moosilauke" OR "mount meru" OR "mount meager" OR "mount massive" OR "mount marsabit" OR "mount marcy" OR "mount mansfield" OR "mount malindang" OR "mount lyell" OR "mount lofty ranges" OR "mount lebanon" OR "mount lebanon range" OR "mount lao" OR "mount kupe" OR "mount kulal" OR "mount kenya" OR "mount jefferson" OR "mount hood" OR "mount heng" OR "mount hasan" OR "mount harvard" OR "mount evans" OR "mount etna" OR "mount erciyes" OR "mount emei" OR "mount elgon" OR "mount elbert" OR "mount edith cavell" OR "mount drummond" OR "mount deception" OR "mount cook range" OR "mount chocorua" OR "mount cameroun" OR "mount cameroon" OR "mount cameron" OR "mount buffalo" OR "mount baldy" OR "mount athos" OR "mount assiniboine" OR "mount ararat" OR "mount aragats" OR "mount aix" OR "mount adams" OR "mosquito range" OR "moroccan atlas" OR "morgan range" OR "moravian-silesian beskids" OR "moorfoot hills" OR "monument peaks" OR "monts groulx" OR "monts faucilles" OR "monts du lyonnais" OR "monts du forez" OR "monts du cantal" OR "monts du beaujolais" OR "monts doré" OR "monts de saïda" OR "monts de lacaune" OR "monts de la madeleine" OR "monts de daïa" OR "monts d'arrée" OR "monti simbruini-ernici" OR "monti sicani" OR "monti sibillini" OR "monti sabini" OR "monti reatini" OR "monti prenestini" OR "monti picentini" OR "monti lepini" OR "monti lattari" OR "monti della laga" OR "monti della daunia" OR "monti carseolani" OR "montes vascos" OR "montes universales" OR "montes obarenes" OR "montes de vitoria" OR "montes de triano" OR "montes de toledo" OR "montes de maría" OR "montes de león" OR "montes de castejón" OR "monte ferru" OR "monte cristo range" OR "monte argentario" OR "monte arci" OR "monte albo" OR "montagnes noires" OR "montagnes françaises" OR "montagne pelée" OR "montagne noire" OR "montagne bourbonnaise" OR "mont péko" OR "mont blanc range" OR "mont blanc massif" OR "mono craters" OR "monitor range" OR "mongun taiga" OR "mongun taiga massif" OR "mongolian plateau" OR "mongolian altai" OR "monche tundra" OR "moldavian plateau" OR "mokra gora" OR "mogollon rim" OR "mitchell ranges" OR "mitchell range" OR "mission ridge" OR "mission range" OR "mishmi hills" OR "miri hills" OR "mineral mts" OR "mikir hills" OR "middle urals" OR "middle ural*" OR "middle atlas" OR "mid north coast" OR "michigan upper peninsula" OR "miao ling" OR "metaliferi mts" OR "meseta patagónica" OR "meseta de somuncurá" OR "meseta central" OR "mescal range" OR "mendip hills" OR "melville range" OR "meliau range" OR "mekongga mts" OR "medicine range" OR "mcpherson range" OR "mcilwraith range" OR "mccullough range" OR "mbooni hills" OR "mbeya range" OR "matobo hills" OR "mataketake range" OR "massive range" OR "massif du nord" OR "massif du carlit" OR "massif du canigou" OR "massif des bornes" OR "massif des arbailles" OR "massif des albères" OR "massif de l'arize" OR "massif de l'agly" OR "massif de la selle" OR "massif de la hotte" OR "massif central" OR "marojejy massif" OR "maritime alps" OR "marble range" OR "mantiqueira range" OR "maneau range" OR "mambilla plateau" OR "malvern hills" OR "malte brun range" OR "maligne range" OR "mala kapela" OR "makonde plateau" OR "major caucasus" OR "main ridge" OR "main range" OR "main caucasian range" OR "mahogany hills" OR "mahale mts" OR "mahadeo hills" OR "madison range" OR "madison plateau" OR "macizo nipe sagua baracoa" OR "macizo de teno" OR "macizo de somiedo" OR "macizo de posets" OR "macizo de montseny" OR "macizo de la maladeta" OR "macizo de cotiella" OR "macizo de anaga" OR "macizo colombiano" OR "maciço do itatiaia" OR "macgillycuddy's reeks" OR "macedon ranges" OR "macdonnell ranges" OR "lyngen alps" OR "lushai hills" OR "lu shan" OR "low tatras" OR "low tatra*" OR "low beskids" OR "low beskid*" OR "low beskid" OR "lovozero massif" OR "lost river range" OR "los montes de málaga" OR "los altos de chiapas" OR "lord range" OR "longwood range" OR "longshou shan" OR "longs peak" OR "longmen mts" OR "long shan" OR "loita hills" OR "lizard range" OR "livingstone range" OR "livingston range" OR "liverpool range" OR "little carpathians" OR "little beskids" OR "little atlas" OR "lisburne hills" OR "limestone ridge" OR "limestone massif" OR "ligurian prealps" OR "ligurian apennines" OR "ligurian alps" OR "lianhua shan" OR "leucite hills" OR "lesser khingan" OR "lesser himalaya" OR "lesser caucasus" OR "lepontine alps" OR "lenglong ling" OR "lena-angara plateau" OR "lena plateau" OR "lemhi range" OR "lely mts" OR "lechtal alps" OR "leaota mts" OR "lateral range" OR "last chance range" OR "lassen peak" OR "las vegas range" OR "large caucasus" OR "lao shan" OR "lammermuir hills" OR "lammermoor range" OR "lamb range" OR "lake range" OR "lake plateau" OR "laji shan" OR "ladakh range" OR "labwor hills" OR "la sila" OR "la serrota" OR "la serra d'en galceran" OR "kysucké beskydy" OR "kyrgyz range" OR "kyrgyz ala-too range" OR "kyrgyz ala too" OR "kwazulu natal midlands" OR "kuznetsk alatau" OR "kurai ridge" OR "kunlun range" OR "kunlun mts" OR "kungey alatau" OR "kun lun" OR "kumaun lesser himalayas" OR "kumaun himalaya" OR "kumaon himalaya*" OR "kular ridge" OR "kubor range" OR "krušné hory" OR "krupinská planina" OR "kroombit tops" OR "krivánska malá fatra" OR "kozie chrbty" OR "koryak range" OR "koppeh dagh" OR "kopet dagh" OR "kopet dag" OR "kongur shan" OR "kondoa irangi hills" OR "kondapalli hills" OR "kolyma upland" OR "kolvitsa massif" OR "kolli hills" OR "kokanee range" OR "kodar ridge" OR "kodar range" OR "kluane ranges" OR "kitchen range" OR "kirthar range" OR "kingston range" OR "khumbu himal" OR "khibiny massif" OR "khentei ridge" OR "khatipara ridge" OR "khasi hills" OR "kharaulakh range" OR "khangai mts" OR "keszthely mts" OR "kazakh uplands" OR "kawich range" OR "kaweka range" OR "kawdy plateau" OR "karpaty zachodnie" OR "karnataka plateau" OR "karlik range" OR "karen hills" OR "karatau ridge" OR "karatau range" OR "kangchenjunga himal" OR "kamnik–savinja alps" OR "kamnik alps" OR "kambui hills" OR "kamasia hills" OR "kalrayan hills" OR "kalba range" OR "kaimanawa range" OR "kaimai range" OR "kaikoura ranges" OR "kaibab plateau" OR "kachin hills" OR "jura mts" OR "juneau icefield" OR "julian prealps" OR "julian alps" OR "jos plateau" OR "jervois range" OR "jebel tuwaiq" OR "jebel sinjar" OR "jebel saghro" OR "jebel bishri" OR "jebel amour" OR "jebel akhdar" OR "jebal-e barez" OR "jebal barez" OR "jbel saghro" OR "jawadhu hills" OR "jawadhi hills" OR "javadi hills" OR "japanese alps" OR "james peak" OR "jaintia hills" OR "jabal sinjar" OR "jabal akhdar" OR "iveti hills" OR "italy range" OR "italian peaks" OR "italian group" OR "italian alps" OR "isalo massif" OR "iranian plateau" OR "inner western carpathians" OR "inland kaikoura range" OR "indian peaks" OR "indian peak range" OR "indian himalayan region" OR "idanre hills" OR "hunua ranges" OR "hume range" OR "humboldt range" OR "huila plateau" OR "house range" OR "hot springs range" OR "hot creek range" OR "horseranch range" OR "horse range" OR "horse heaven hills" OR "homathko icefield" OR "hokonui hills" OR "hohonu range" OR "hoggar massif" OR "hoback range" OR "hissar range" OR "hindu raj" OR "hindu kush" OR "hindu kush himalayan region" OR "hindu kush himalaya*" OR "hindu kush himalaya* region" OR "himachal himalayas" OR "hill country" OR "high tauern" OR "high tatras" OR "high tatra*" OR "high atlas" OR "high atlas range" OR "hidaka range" OR "herveys range" OR "hervey range" OR "herberton range" OR "henry mts" OR "hengduan shan" OR "heng shan" OR "heli shan" OR "helan shan" OR "helan range" OR "hayes range" OR "haut tell tunisien" OR "harz national park" OR "harts range" OR "hart ranges" OR "harrat al-sham" OR "harghita mts" OR "handies peak" OR "hamiguitan range" OR "hamersley range" OR "hall range" OR "haldon hills" OR "hakarimata range" OR "gurktal alps" OR "guayana shield" OR "gros ventre range" OR "grey range" OR "gregory rift" OR "greenwater range" OR "greenhorn range" OR "greater khingan" OR "greater caucasus" OR "great western tiers" OR "great rift valley" OR "great range" OR "great escarpment" OR "great dividing range" OR "great basin ranges" OR "grays range" OR "grays peak" OR "grays hills" OR "gray hills" OR "gravelly range" OR "grant range" OR "granite range" OR "grands causses" OR "grandes rousses" OR "grand mesa" OR "grand canyon" OR "gran sasso" OR "gran paradiso" OR "grampian range" OR "graian alps" OR "gore range" OR "gorce mts" OR "gold range" OR "goat rocks" OR "goat range" OR "glens of antrim" OR "glenlyon range" OR "glarus alps" OR "glacier peak" OR "gissar range" OR "gillis range" OR "gibraltar range" OR "german alps" OR "german alpine foreland" OR "gerecse mts" OR "georgian caucasus" OR "georgia blue ridge" OR "gelibolu yarimadasi" OR "gawler ranges" OR "gausdal vestfjell" OR "garo hills" OR "garnet range" OR "garhwal himalaya" OR "garfield peaks" OR "garfield hills" OR "gaoligong shan" OR "gaoligong mts" OR "gangdise range" OR "ganesh himal" OR "gammon ranges" OR "gallatin range" OR "gailtal alps" OR "gabilan range" OR "gabbs valley range" OR "fuuta jaloo" OR "futa jallon" OR "futa djalon" OR "fuentes carrionas" OR "fruška gora" OR "front range" OR "frenchman hills" OR "french pyrenees" OR "french jura" OR "french alps" OR "fraser plateau" OR "franconian jura" OR "fra cristobal range" OR "fox range" OR "fouta djallon" OR "flint hills" OR "flint creek range" OR "flinders range" OR "flathead range" OR "flat tops" OR "fish springs range" OR "fish lake plateau" OR "fish creek range" OR "first range" OR "finisterre range" OR "fila brunqueña" OR "fergana range" OR "fennoscandian shield" OR "fell lapland" OR "fawnie range" OR "farallones de cali" OR "falaise de banfora" OR "fairweather range" OR "făgăraș mts" OR "everard ranges" OR "european upland*" OR "european alps" OR "etna volcan*" OR "etna mount*" OR "epi island" OR "ely range" OR "elk ridge" OR "elk range" OR "elgeyo escarpment" OR "elbow range" OR "el garraf" OR "eje neovolcanico mexicano" OR "egan range" OR "edwards plateau" OR "edough massif" OR "ecuadorian andes" OR "eastern tian shan" OR "eastern tatras" OR "eastern tannu ola" OR "eastern sudetes" OR "eastern sayan" OR "eastern satpura range" OR "eastern rift" OR "eastern ridge" OR "eastern rhodopes" OR "eastern range" OR "eastern pyrenees" OR "eastern pre-pyrenees" OR "eastern pamirs" OR "eastern middle atlas" OR "eastern main range" OR "eastern kunlun" OR "eastern hindu kush" OR "eastern hills" OR "eastern high atlas" OR "eastern hellenides" OR "eastern hajar" OR "eastern ghat" OR "eastern ghats" OR "eastern escarpment" OR "eastern coastal range" OR "eastern caucasus" OR "eastern carpathians" OR "eastern brooks range" OR "eastern betics" OR "eastern beskids" OR "eastern arc mts" OR "eastern anti-atlas" OR "eastern alps" OR "eastern adirondacks" OR "east* carpathian*" OR "east usambara" OR "east range" OR "east kunlun" OR "east humboldt range" OR "east african plateau" OR "dzungarian alatau" OR "dzhugdzhur range" OR "dugway range" OR "duck creek range" OR "dry andes" OR "drummond range" OR "douglas ranges" OR "douglas range" OR "dorsale tunisienne" OR "dorsal gallega" OR "domeyko range" OR "dolomitic alps" OR "dolomiti lucane" OR "dogtooth range" OR "dobrogea plateau" OR "djebel sinjar" OR "djebel amour" OR "dinaric alps" OR "dinara massif" OR "dieng volcanic complex" OR "dieng plateau" OR "diamond range" OR "dial range" OR "diablo range" OR "dhaulagiri himalaya" OR "dhauladhar range" OR "dévoluy massif" OR "deserters range" OR "desert range" OR "deseado massif" OR "deosai plateau" OR "deli jovan" OR "defiance plateau" OR "deep creek range" OR "deccan plateau" OR "dean hill" OR "daxue shan" OR "dawson range" OR "darvaz range" OR "darling range" OR "daqing shan" OR "danghe nanshan" OR "danghe nan shan" OR "dandenong ranges" OR "dafla hills" OR "dachstein mts" OR "dabie mts" OR "daba shan" OR "da hinggan" OR "da hinggan range" OR "da hinggan ling" OR "da ba shan" OR "czech carpathians" OR "cypress hills" OR "cumbres calchaquíes" OR "cumberland plateau" OR "culebra range" OR "crocker range" OR "crimean mts" OR "crawford range" OR "craigieburn range" OR "cottian alps" OR "cordón del portillo" OR "cordón del plata" OR "conondale range" OR "confusion range" OR "columbia plateau" OR "columbia icefield" OR "columbia hills" OR "colorado rockies" OR "colorado plateau" OR "coconino plateau" OR "coast range" OR "cleopatra's needle" OR "clent hills" OR "clemenceau icefield" OR "clear range" OR "clear hills" OR "clayton ridge" OR "clark range" OR "cimarron range" OR "chyulu hills" OR "churia hills" OR "chulyshman plateau" OR "chukotka upland" OR "chugach range" OR "chota nagpur plateau" OR "chola shan" OR "choč mts" OR "chittagong hill tracts" OR "chiricahua peak" OR "chinese wall" OR "chinese loess plateau" OR "chin hills" OR "chimantá massif" OR "chilkat range" OR "chilcotin ranges" OR "chilcotin plateau" OR "chiemgau alps" OR "chichester ranges" OR "cheviot hills" OR "chersky ridge" OR "chersky range" OR "cherry creek range" OR "cherang'any hills" OR "chatkal range" OR "chartreuse massif" OR "chapada dos veadeiros" OR "chapada dos guimarães" OR "chapada do araripe" OR "chapada diamantina" OR "chapada das mesas" OR "chapada das mangabeiras" OR "changbai mts" OR "chaîne des puys" OR "chaîne des matheux" OR "chagai hills" OR "cerros orientales" OR "cerros del sira" OR "cerros de escazú" OR "cerros colorados" OR "cerro guaiquinima" OR "cerro duida" OR "centre hills" OR "central-eastern alps" OR "central wind river range" OR "central western ghats" OR "central wasatch range" OR "central volcanic zone" OR "central venezuelan andes" OR "central uplands" OR "central tian shan" OR "central tell atlas" OR "central taurus" OR "central tablelands" OR "central sudetes" OR "central southern pyrenees" OR "central siberian plateau" OR "central ranges" OR "central range" OR "central pyrenees" OR "central pre-pyrenees" OR "central plateau" OR "central pindus" OR "central oregon coast range" OR "central north caucasus" OR "central moravian carpathians" OR "central middle atlas" OR "central mexican plateau" OR "central massif" OR "central massif central" OR "central madison range" OR "central lesser himalaya*" OR "central kunlun" OR "central japanese alps" OR "central himalaya*" OR "central high plateau" OR "central high atlas" OR "central front ranges" OR "central front range" OR "central cumberland plateau" OR "central caucasus" OR "central betics" OR "central balkan mts" OR "central appalachians" OR "central apennines" OR "central anti-atlas" OR "central andes" OR "central america volcanic arc" OR "central alps" OR "central alborz mts" OR "centennial range" OR "ceahlău massif" OR "causses du quercy" OR "caucasus mts" OR "caucasus major" OR "cathedral range" OR "cathedral group" OR "catena costiera" OR "catalan coastal range" OR "cascade* range" OR "cascade range" OR "carter range" OR "carson range" OR "carso huasteco" OR "carpathian mts" OR "carnic prealps" OR "carnic alps" OR "cariboo plateau" OR "cardamom mts" OR "cardamom hills" OR "capitol reef" OR "capitol hills" OR "cape midlands" OR "canyon ranges" OR "cantabrian range" OR "cantabrian mts" OR "candelaria hills" OR "canadian shield" OR "canadian rockies" OR "canadian appalachians" OR "campbell range" OR "campbell hills" OR "campanian apennines" OR "cameroon line" OR "camelsfoot range" OR "călimani mts" OR "california coast ranges" OR "california cascades" OR "calar del río mundo" OR "calabrian apennines" OR "burster range" OR "bureya massif" OR "bungo range" OR "bullfrog hills" OR "bukit tigapuluh" OR "bukit barisan" OR "buffalo hills" OR "buda hills" OR "buckskin range" OR "bucegi mts" OR "browning range" OR "brooks range" OR "broken hills" OR "brindabella range" OR "bridger range" OR "brenta group" OR "brecon beacons" OR "brazilian plateau" OR "brazeau range" OR "boz dag*" OR "bowland fells" OR "bow range" OR "boundary ranges" OR "bosche range" OR "bornes massif" OR "border ranges" OR "borborema plateau" OR "book cliffs" OR "bong range" OR "bonar range" OR "bomi hills" OR "bolu massif" OR "bolsón de mapimí" OR "bolivian plateau" OR "bolivian andes" OR "bolaven volcanic field" OR "bolaven plateau" OR "bohemian massif" OR "bohemian forest" OR "bohea hills" OR "bogotá savanna" OR "bogong high plains" OR "bogda shan" OR "bogd uul" OR "blue range" OR "blue mts" OR "bloods range" OR "blidean atlas" OR "blackwater range" OR "blackall range" OR "black rock range" OR "black range" OR "black hills" OR "black forest" OR "black divide" OR "biu plateau" OR "bitterroot range" OR "bismarck range" OR "binalud range" OR "bihor mts" OR "bighorn range" OR "big salmon range" OR "big range" OR "big caucasus" OR "bhutan himalaya" OR "betic system" OR "betic chain" OR "baetic system" OR "baetic chain" OR "beskid sadecki" OR "bernina massif" OR "bernese alps" OR "berkshire hills" OR "bergamo alps" OR "berchtesgaden alps" OR "benton range" OR "bendor range" OR "ben ohau range" OR "belted range" OR "bellenden ker range" OR "belledonne massif" OR "belianske tatras" OR "bei shan" OR "bayuda volcanic field" OR "bayan har shan" OR "bavarian forest" OR "bavarian alps" OR "bauges massif" OR "bateke plateau" OR "barrington tops" OR "barrier range" OR "barnett hills" OR "barisan range" OR "barguzin ridge" OR "barguzin range" OR "barail range" OR "bannock range" OR "banjaran crocker" OR "bandiagara escarpment" OR "banded range" OR "bamenda grassfields" OR "baltic shield" OR "ball range" OR "balkan range*" OR "balkan mts" OR "baldy peak" OR "balaton uplands" OR "bakossi mts" OR "bailadila range" OR "baikal ridge" OR "baikal range*" OR "baetic range*" OR "betic range*" OR "badzhal range" OR "backbone ranges" OR "baba budan hills" OR "austrian central alps" OR "austrian alps" OR "australian alps" OR "aurora range" OR "atlas range*" OR "atlas marocain" OR "atlas chain*" OR "atlas blidéen" OR "atlantic plateau" OR "atewa range" OR "atbashi range" OR "asturian massif" OR "assam himalaya" OR "aspen range" OR "arthur range" OR "arrowsmith range" OR "arrow canyon range" OR "armorican massif" OR "arjin shan" OR "argentinian andes" OR "arctic ocean islands" OR "aravalli range" OR "aravali range" OR "araucaria plateau" OR "arakan yoma" OR "arakan hills" OR "aragonese pyrenees" OR "aquarius plateau" OR "apuseni mts" OR "apuan alps" OR "aprada tepui" OR "appalachian* range" OR "appalachian ridges" OR "appalachian plateaus" OR "aorangi range" OR "anvil range" OR "antrim plateau" OR "anti taurus" OR "antelope range" OR "annapurna range" OR "annapurna himalaya" OR "ankaratra massif" OR "angolan escarpment" OR "andringitra massif" OR "andes peruanos" OR "andes fueguinos" OR "andean plateau" OR "anatolian plateau" OR "anaimalai hills" OR "anaconda range" OR "anabar plateau" OR "amne machin" OR "ammergauer alpen" OR "ammergau alps" OR "alxa plateau" OR "altyn tagh" OR "altun shan" OR "altun mts" OR "altos de jalisco" OR "altiplano cundiboyacense" OR "altay sayan" OR "altay range" OR "altai sayan" OR "altai range" OR "altai mts" OR "alpine-himalayan orogenic belt" OR "alpine-himalayan belt" OR "alpine lakes area" OR "alpide belt" OR "alpi orobie" OR "alpes maritimes" OR "almăj depression" OR "allies group" OR "allgäu alps" OR "allegheny plateau" OR "allegheny front" OR "alishan national scenic area" OR "algerian atlas" OR "alexander range" OR "alexander archipelago" OR "aleutian ridge" OR "aleutian range" OR "aleutian arc" OR "aleut* islands" OR "aldan plateau" OR "alborz mts" OR "albion range" OR "alaska range" OR "alashan plateau" OR "ajodhya hills" OR "ailao shan" OR "ailao mts" OR "aiguilles rouges" OR "aiguilles rouges massif" OR "aibga ridge" OR "ahaggar mts" OR "adrar des ifoghas" OR "adobe range" OR "adirondack mts" OR "adirondack high peaks" OR "adelbert range" OR "adamawa plateau" OR "adamawa massif" OR "absaroka range" OR "abruzzo apennine*" OR "abruzzi apennines" OR "aberdare range" OR "jebel tessala" OR rockies OR andes OR betics OR himalaya OR appalachian* OR andean OR caucasus OR urals OR carpathians OR himachal OR apennine* OR apennines OR pyrenees OR jura OR tianshan OR qinling OR adirondacks OR altiplano OR zagros OR ardennes OR vosges OR kunlun OR babor* OR kilimanjaro OR witwatersrand OR tatras OR alborz OR palisades OR vesuvius OR harz OR nilgiris OR karakoram OR guadarrama OR qiangtang OR pennines OR anti-atlas OR vercors OR beishan OR cévennes OR grampians OR sudetes OR morvan OR sikhote-alin OR dinarides OR drakensberg OR schwarzwald OR teide OR karpaty OR karakorum OR fiordland OR beskids OR nanshan OR ruapehu OR altyn-tagh OR pieniny OR velebit OR uludağ OR canyonlands OR cotswolds OR waterberg OR katahdin OR jinggangshan OR laoshan OR rondane OR hindukush OR vanoise OR emmental OR cairngorms OR taunus OR kohala OR medvednica OR odenwald OR pilat OR adamello OR tatry OR pollino OR ecrins OR pirin OR gorce OR hunsrück OR yinshan OR mecsek OR hellenides OR sabalan OR trans-himalaya OR djurdjura OR alay OR arves OR majella OR ruwenzori OR jirisan OR aubrac OR belledonne OR snowdonia OR breheimen OR jotunheimen OR bakony OR madonie OR burren OR daqingshan OR udzungwa OR sauerland OR soutpansberg OR spessart OR burda OR scandes OR chablais OR katla OR matese OR nebrodi OR papuk OR alishan OR hardangervidda OR pelion OR cassiars OR fenghuangshan OR monfragüe OR aspromonte OR westerwald OR kopaonik OR cederberg OR brandberg OR vogelsberg OR cangshan OR gorongosa OR nemrut OR nassa OR gaoligongshan OR sahand OR taillefer OR kalnik OR tienshan OR mengshan OR biokovo OR tjörnes OR nyainqentanglha OR dahra OR svartisen OR parnassos OR dayaoshan OR karadağ OR gallot OR vitosha OR pamir-alai OR mátra OR pilanesberg OR ségala OR pre-pyrenees OR kebnekaise OR blouberg OR hualalai OR edough OR rooiberg OR korab OR vourinos OR karadag OR sumava OR putorana OR elburz OR tafelberg OR okstindan OR durmitor OR valdres OR verno OR appennini OR donggan OR bozburun OR behistun OR učka OR xueshan OR yaoshan OR bibans OR khamar-daban OR karasjok OR winterberg OR alburni OR chornohora OR cézallier OR baviaanskloof OR planèze OR changthang OR tsinling OR dovrefjell OR giffre OR cedarberg OR jesenik OR kozjak OR warrumbungles OR jablanica OR gennargentu OR changtang OR zarkos OR kopetdag OR crestones OR magaliesberg OR pamir-alay OR langeberg OR lauzière OR daliangshan OR andringitra OR funiushan OR ukaguru OR transhimalaya OR gesäuse OR sidobre OR ozren OR presanella OR gariwerd OR svilaja OR macelj OR beaufortain OR prokletije OR fongen OR krndija OR finnmarksvidda OR dilj OR mariovo OR psunj OR swartruggens OR jastrebac OR ankaratra OR hardangerjøkulen OR karwendel OR anti-lebanon OR beni-chougrane OR branisko OR tribeč OR piketberg OR stolowe OR yamanlar OR kithairon OR parnitha OR poľana OR pratomagno OR slavyanka OR mosor OR mavrovouni OR titteri OR atakor OR galičica OR alburz OR brownsberg OR grammos OR hochschwab OR mainpat OR saltfjellet OR artense OR rtanj OR rätikon OR chataigneraie OR auyantepui OR rumija OR kasikasima OR wolkberg OR ograzhden OR drauzug OR mianshan OR majevica OR svydovets OR chitteri OR chřiby OR žiar OR anyemaqen OR tavan-bogd OR gantrisch OR lovćen OR bleia OR forollhogna OR tomaros OR gasherbrum OR lévézou OR storskarven OR haukelifjell OR aladaglar OR khibins OR trollheimen OR kvamshesten OR nordfjella OR daxueshan OR sinjajevina OR beljanica OR fangdoushan OR kožuf OR hualālai OR tsambagarav OR jinpingshan OR diamir OR jakupica OR sarisariñama OR qangtang OR tzoumerka OR vindelfjällen OR elborz OR golak OR escandorgue OR xerovouni OR ryfylkeheiane OR ljubišnja OR carpati OR aladagh OR malundwe OR orjen OR sunndalsfjella OR drakensberge OR waifangshan OR sausfjellet OR javorie OR jiulingshan OR ottadalen OR kukavica OR anysberg OR suurberg OR blåfjella OR kugitangtau OR hamar-daban OR norefjell OR vlahina OR gruvefjellet OR tsagan-daban OR filefjell OR romsdalsalpane OR stølsheimen OR sokoró OR maleshevo OR yengisogat OR babatag OR antichasia OR anti-apennines OR karawanks OR ammergebirge OR cserhat OR toven OR långfjället OR goljak OR blefjell OR bogdashan OR erimanthos OR skjækerfjella OR høgtuva OR ålfotbreen OR meygal OR borzsony OR ambohiby OR kharkhiraa OR charchiraa OR murovdag OR steenkampsberg OR tsinling-dabashan OR jurjura OR kjølhaugan OR orvilos OR valamara OR pentadactylos OR plješivica OR artfjället OR roggeveldberge OR tendurek OR skaget OR ilfjellet OR trysilfjellet OR pothigai OR slievenamon OR maumturks OR kamakou OR vtacnik OR jabllanicë OR jundushan)

OR

AB=(alpine OR subalpine OR montane OR submontane OR mountain OR mountainous OR upland OR highland OR "high* elevation" OR "high* altitude" OR summit OR yunga OR puna OR paramo OR glacial OR himalayan OR carpathian OR caucasian OR appalachian OR cordilleran OR pyrenean OR tibetan OR cantabrian OR altiplano OR afromontane OR afroalpine OR madrean OR paramo OR yunga OR puna OR upland OR "eastern arc" OR "mid* elevation" OR "mid* altitude" OR cantabrian OR pontic OR glacial OR summit OR volcanic OR crater OR paramo$ OR páramo$ OR subkryal OR "sub kryal" OR tarn OR "glacial pond$" OR "glacier fed aquatic" OR "glacier fed catchment$" OR "glacier fed channel$" OR "glacier fed pond$" OR "glacier fed spring$" OR "glacier fed water$" OR "thaw pond$" OR "mountain torrent$" OR "altitud* decline" OR "altitud* decrease" OR "altitud* distribution$" OR "altitud* diversit*" OR "altitud* gradient$" OR "altitud* increase" OR "altitud* limit$" OR "altitud* pattern$" OR "altitud* range$" OR "altitud* shift$" OR "altitud* transect$" OR "altitud* variabilit*" OR "altitud* variation$" OR "altitud* zonation$" OR "campo* rupestre$" OR "cryo oromediterranean" OR "elevation* decline" OR "elevation* decrease" OR "elevation* distribution$" OR "elevation* diversit*" OR "elevation* gradient$" OR "elevation* increase" OR "elevation* limit$" OR "elevation* pattern$" OR "elevation* range$" OR "elevation* shift$" OR "elevation* transect$" OR "elevation* variabilit*" OR "elevation* variation$" OR "elevation* zonation$" OR "glacial retreat$" OR "gradient$ of altitude" OR "gradient$ of elevation" OR "hautes terres" OR "high* altitude$" OR "high* elevation$" OR "rock glacier$" OR "sky island$" OR "tropical glacier$" OR afroalpine OR "afro alpine" OR afromontane OR "afro montane" OR alpine OR alpino$ OR altiplano$ OR banjaran OR cordilheira$ OR cordill?re$ OR cordillera$ OR daglar OR daglari OR fjell OR fjellkjede OR gebirge OR gunung OR highland$ OR khrebet OR montane OR mountain$ OR mountainous OR mountaintop$ OR nunatak$ OR pegunungan OR planina OR pohorie OR serra$ OR serralada$ OR sierra$ OR stratovolcano* OR "strato volcano*" OR subalpine OR submontane OR "sub montane" OR upland$ OR volcano* OR "glacier outflow$" OR glaciolacustrine OR "glacio lacustrine" OR submountainous OR "sub mountainous" OR "żywiec beskids" OR "žumberak mts" OR "zomba plateau" OR "zillertal alps" OR "zarafshan range" OR "zanskar range" OR "zane hills" OR "zagros mts" OR "yunnan-guizhou plateau" OR "yungui plateau" OR "yukon-tanana uplands" OR "yu shan" OR "yorkshire dales" OR "yi shan" OR "yenisei ridge" OR "yenisei range" OR "yellowstone plateau" OR "yangming shan" OR "xue shan" OR "xiong'er shan" OR "xiao shan" OR "xiao hinggan ling" OR "x hills" OR "wyoming range" OR "wudang shan" OR "wooley range" OR "wind river range" OR "willapa hills" OR "wild horse range" OR "wieliczka foothills" OR "wichita mts" OR "whitefish range" OR "white river plateau" OR "white range" OR "white pine range" OR "white mts" OR "white divide" OR "white carpathians" OR "whistler range" OR "western venezuelan andes" OR "western tian shan" OR "western taurus" OR "western tatras" OR "western tatra*" OR "western tannu ola" OR "western sudetes" OR "western sayan" OR "western sahara atlas" OR "western romanian carpathians" OR "western rhodopes" OR "western range" OR "western pyrenees" OR "western pamirs" OR "western pacific islands" OR "western middle atlas" OR "western main range" OR "western lesser himalaya*" OR "western kunlun" OR "western hindu kush" OR "western himalaya*" OR "western high atlas" OR "western hellenides" OR "western hajar" OR "western ghat" OR "western ghats" OR "western foothills" OR "western caucasus" OR "western carpathians" OR "western bieszczady" OR "western betics" OR "western beskids" OR "western balkan mts" OR "western anti-atlas" OR "western alps" OR "west* carpathian*" OR "west* african uplands" OR "west usambara" OR "west range" OR "west kunlun" OR "west coast ranges" OR "west coast range" OR "wenatchee ridge" OR "wellington range" OR "weepah hills" OR "webster range" OR "wassuk range" OR "washakie range" OR "wasatch range" OR "walker range" OR "walden ridge" OR "waitakere ranges" OR "waianae range" OR "vosges mts" OR "voras mts" OR "volga uplands" OR "volcanic hills" OR "volcanes de colima" OR "vitim plateau" OR "virginia range" OR "villány mts" OR "victorian alps" OR "victoria range" OR "vértes hills" OR "vermilion range" OR "verkhoyansk ridge" OR "verkhoyansk range" OR "vercors massif" OR "venezuelan coastal range" OR "venezuelan andes" OR "venetian prealps" OR "velka fatra" OR "velika kapela" OR "velence hills" OR "vanoise massif" OR "valais alps" OR "val di non" OR "usun apau plateau" OR "usambara mts" OR "ural mts" OR "upper atlas" OR "united states range" OR "umbrian apennines" OR "ulugh muztagh" OR "ulagan plateau" OR "ulaan taiga" OR "ukrainian carpathians" OR "uinkaret plateau" OR "udokan ridge" OR "udokan range" OR "tyan shan" OR "two thumb range" OR "twin range" OR "tuscan–emilian apennines" OR "turkestan range" OR "tunka ridge" OR "tunisian dorsale" OR "tunisian atlas" OR "tunguska plateau" OR "tukuringra ridge" OR "tukuringra range" OR "tug hill" OR "tsingy de bemaraha" OR "tsambagarav ridge" OR "truong son range" OR "truckee range" OR "tropical andes" OR "trinity range" OR "trinity alps" OR "trialeti range" OR "trara* mount*" OR "transylvanian plateau" OR "transylvanian plain" OR "transverse ranges" OR "trans-mexican volcanic belt" OR "trans-ili alatau" OR "tosco romagnolo apennines" OR "torlesse range" OR "toquima range" OR "tongbai shan" OR "tomkinson ranges" OR "tokaj mts" OR "toiyabe range" OR "tobin range" OR "tobacco range" OR "toano range" OR "timpahute range" OR "tigray plateau" OR "tien shan" OR "tibetan plateau" OR "tianmu shan" OR "tian shan" OR "thuringian forest" OR "thompson peaks" OR "thompson peak" OR "thomas range" OR "thirtymile range" OR "the sperrins" OR "the snowies" OR "the maniototo" OR "the hunter's hills" OR "the hokonuis" OR "the catlins" OR "the ancient wall" OR "teton range" OR "tethyan orogenic belt" OR "terskey ala-too" OR "termit massif" OR "terminal range" OR "tenmile range" OR "temblor range" OR "tell atlas" OR "telangana plateau" OR "te urewera" OR "tchabal mbabo" OR "tawau hills" OR "taurus range" OR "taurus mts" OR "tatras mts" OR "tassili n'ajjer" OR "taringatura hills" OR "tararua range" OR "tannu ola" OR "tanggula mts" OR "talkeetna mts" OR "talas alatau" OR "taita hills" OR "taillefer massif" OR "taihang shan" OR "tai shan" OR "tadrart acacus" OR "système ibérique" OR "synnyr massif" OR "swiss prealps" OR "swiss jura" OR "swiss alps" OR "świętokrzyskie mts" OR "sweetgrass hills" OR "swannell ranges" OR "swan range" OR "swabian jura" OR "suva planina" OR "surrey hills" OR "superior upland" OR "suntar-khayata ridge" OR "suntar-khayata range" OR "summit ridge" OR "sumava national park" OR "sulphur spring range" OR "suisse normande" OR "submeseta sur" OR "submeseta norte" OR "sublette range" OR "sublett range" OR "stubai alps" OR "stuart range" OR "strzelecki ranges" OR "strathbogie ranges" OR "storm ridge" OR "stolowe mts" OR "stirling range" OR "stillwater range" OR "stikine plateau" OR "stauning alps" OR "stara planina" OR "stanovoy range" OR "stanovik range" OR "stanford range" OR "sredna gora" OR "sredinny range" OR "spotted range" OR "spectrum range" OR "specter range" OR "spatsizi plateau" OR "spanish pyrenees" OR "spanish peaks" OR "southwestern pyrenees" OR "southwestern massif central" OR "southwestern alps" OR "southland hills" OR "southern zagros" OR "southern wind river range" OR "southern western ghats" OR "southern wasatch range" OR "southern urals" OR "southern ural*" OR "southern uplands" OR "southern schwarzwald" OR "southern scandes" OR "southern sawatch range" OR "southern rockies" OR "southern rhodopes" OR "southern range" OR "southern peaks" OR "southern patagonian andes" OR "southern oregon coast range" OR "southern oregon cascades" OR "southern new hampshire" OR "southern limestone alps" OR "southern japanese alps" OR "southern great escarpment" OR "southern cumberland plateau" OR "southern catskills" OR "southern carpathians" OR "southern california coast ranges" OR "southern calcareous alps" OR "southern black hills" OR "southern black forest" OR "southern bakony" OR "southern aravalli range" OR "southern appalachians" OR "southern appalachian ridges" OR "southern apennines" OR "southern andes" OR "southern alps" OR "southern african ranges" OR "southern adirondacks" OR "southeastern pyrenees" OR "southeastern massif central" OR "southeastern alps" OR "south western caucasus" OR "south western alps" OR "south pennines" OR "south pare" OR "south pahroc range" OR "south eastern caucasus" OR "south eastern alps" OR "south downs" OR "south altai" OR "soufrière hills" OR "sonoma range" OR "song shan" OR "somali plateau" OR "solution range" OR "solitude range" OR "snowy range" OR "snowcrest range" OR "snake river range" OR "snake range" OR "slovenian alps" OR "slovak paradise" OR "slovak karst" OR "slovak carpathians" OR "slate range" OR "slate mts" OR "skopska crna gora" OR "skippers range" OR "siwalik range" OR "sitheri hills" OR "sistema periférico" OR "sistema mediterráneo catalán" OR "sistema ibérico" OR "sistema de tandilia" OR "sistema central" OR "sistema bético" OR "sirumalai hills" OR "sirente velino" OR "singatse range" OR "simpson park range" OR "simi hills" OR "silvretta massif" OR "silver peaks range" OR "silver peak range" OR "silesian foothills" OR "silesian beskids" OR "sila piccola" OR "sila greca" OR "sila grande" OR "sikkim himalayas" OR "sifton ranges" OR "shoshone range" OR "shiwan dashan" OR "shivwits plateau" OR "shimba hills" OR "sheep range" OR "sheep creek range" OR "shan hills" OR "shaler range" OR "seymour range" OR "sevier plateau" OR "sette daban ridge" OR "sette daban range" OR "setesdal vesthei" OR "setesdal ryfylke" OR "servarayan hills" OR "serres de llevant" OR "serranías chiquitanas" OR "serranía del litoral" OR "serranía del interior" OR "serranía del aguaragüe" OR "serrania de ronda" OR "serranía de pirre" OR "serranía de macuira" OR "serranía de los yariguíes" OR "serranía de los paraguas" OR "serranía de los churumbelos" OR "serranía de las quinchas" OR "serranía de cuenca" OR "serranía de chiribiquete" OR "serra parima" OR "serra negra" OR "serra mariola" OR "serra jeci" OR "serra grande" OR "serra geral" OR "serra geral de goiás" OR "serra gaúcha" OR "serra dourada" OR "serra dos tapes" OR "serra dos pirineus" OR "serra dos órgãos" OR "serra dos cristais" OR "serra dos cocais" OR "serra dos cavalos" OR "serra dos carajás" OR "serra dos aimorés" OR "serra do xistral" OR "serra do tombador" OR "serra do tabuleiro" OR "serra do suído" OR "serra do sincorá" OR "serra do rio do rastro" OR "serra do ramalho" OR "serra do papagaio" OR "serra do moco" OR "serra do marão" OR "serra do mar" OR "serra do lajeado" OR "serra do gandarela" OR "serra do estrondo" OR "serra do espinhaço" OR "serra do espigão" OR "serra do divisor" OR "serra do courel" OR "serra do cipó" OR "serra do caparaó" OR "serra do caldeirão" OR "serra do cachimbo" OR "serra do cabral" OR "serra d'irta" OR "serra d'espadà" OR "serra del montsant" OR "serra del catllaràs" OR "serra del cadí" OR "serra de uruburetama" OR "serra de são miguel" OR "serra de são mamede" OR "serra de santana" OR "serra de sant gervàs" OR "serra de paranapiacaba" OR "serra de montemuro" OR "serra de montejunto" OR "serra de monchique" OR "serra de maracaju" OR "serra de les gavarres" OR "serra de joão do vale" OR "serra de gerês" OR "serra de botucatu" OR "serra de baturité" OR "serra das matas" OR "serra das confusões" OR "serra das araras" OR "serra da saudade" OR "serra da peneda" OR "serra da nogueira" OR "serra da neve" OR "serra da mocidade" OR "serra da meruoca" OR "serra da mantiqueira" OR "serra da malcata" OR "serra da leba" OR "serra da jacobina" OR "serra da graciosa" OR "serra da estrela" OR "serra da capivara" OR "serra da capelada" OR "serra da cantareira" OR "serra da canastra" OR "serra da borda" OR "serra da bodoquena" OR "serra da bocaina" OR "serra da aratanha" OR "serra calderona" OR "serra azul" OR "serbian carpathians" OR "sentinel range" OR "sentinel hills" OR "selwyn range" OR "selennyakh ridge" OR "selawik hills" OR "selamiut range" OR "seaward kaikoura range" OR "seaman range" OR "scotchman peak*" OR "schrader range" OR "schell creek range" OR "scenic rim" OR "sayan mts" OR "sawtooth ridge" OR "sawtooth range" OR "sawatch range" OR "saur ridge" OR "satpura range" OR "sarntal alps" OR "sarek mts" OR "santa lucia range" OR "sandwich range" OR "sandhills range" OR "sand springs range" OR "san rafael swell" OR "san juan mts" OR "san francisco peaks" OR "salt river range" OR "salt range" OR "salair ridge" OR "sacramento mts" OR "sabana de bogotá" OR "russian caucasus" OR "russian altai" OR "rumpi hills" OR "rudnyi altai" OR "ruby range" OR "ruahine range" OR "royston hills" OR "royal group" OR "rough ridge" OR "romanian carpathians" OR "rolwaling himal" OR "rocky range" OR "rocky mts" OR "rock and pillar range" OR "robinson ranges" OR "robertson range" OR "roan plateau" OR "roan cliffs" OR "river mts" OR "ritter range" OR "rimutaka range" OR "rila mts" OR "rila geopark" OR "richmond range" OR "richmond hills" OR "rhodope mts" OR "rhenish massif" OR "rewa plateau" OR "reveille range" OR "retezat mts" OR "resting spring range" OR "redcloud peak" OR "red sea hills" OR "red hills" OR "raukumara range" OR "rattlesnake hills" OR "raton mesas" OR "range of the shelter" OR "rampart range" OR "ram range" OR "rakwana hills" OR "rakhine yoma" OR "rakaposhi range" OR "rajmahal hills" OR "raja ampat islands" OR "rainbow range" OR "ragged range" OR "quinn canyon range" OR "queen elizabeth range" OR "quantock hills" OR "qinling dabashan" OR "qingzang gaoyuan" OR "qinghai nanshan" OR "qin ling" OR "qilian mts" OR "qian shan" OR "qaidam basin" OR "pyrenees range" OR "putu range" OR "putorana plateau" OR "purcell range" OR "puna de atacama" OR "puertos de beceite" OR "provence alps" OR "price range" OR "presidential range" OR "president range" OR "prealpi giulie" OR "prealpi bergamasche" OR "préalpes de nice" OR "požeška gora" OR "považský inovec" OR "pouakai range" OR "portneuf range" OR "porcupine hills" OR "polonyna borzhava" OR "polish carpathians" OR "polar urals" OR "polar ural*" OR "polar range" OR "pohronský inovec" OR "pocono plateau" OR "po hills" OR "pliny range" OR "plateau of tibet" OR "plateau of mozambique" OR "plateau de millevaches" OR "plateau de danyi" OR "planalto da borborema" OR "planalto brasileiro" OR "piute range" OR "piton des neiges" OR "piton de la fournaise" OR "pisa range" OR "pir panjal range" OR "pioneer range" OR "pintler range" OR "pine grove hills" OR "pine forest range" OR "pilot range" OR "pilot butte" OR "pikikiruna range" OR "picos de europa" OR "pico island" OR "pico da neblina" OR "petermann ranges" OR "peruvian andes" OR "persian plateau" OR "pennine alps" OR "peninsular ranges" OR "peñas de cervera" OR "peloritani range" OR "pekulney range" OR "pegu yoma" OR "pavant range" OR "patagonian andes" OR "park range" OR "paria plateau" OR "pare mts" OR "páramos de la lora" OR "páramo de sumapaz" OR "páramo de pisba" OR "páramo de cruz verde" OR "paradise range" OR "paparoa range" OR "pangong range" OR "pancake range" OR "panamint range" OR "pan ling" OR "pampa del tamarugal" OR "pamir mts" OR "palatinate forest" OR "palani hills" OR "palamalai hills" OR "pakistan himalaya" OR "pahute mesa" OR "pahranagat range" OR "pah rah range" OR "paeroa range" OR "pacific ranges" OR "pacific coast ranges" OR "pachaimalai hills" OR "pab range" OR "ozark plateau" OR "ozark foothills" OR "owen stanley range" OR "outer western carpathians" OR "outer eastern carpathians" OR "ougarta range" OR "ouarsenis range" OR "ouarsenis massif" OR "ötztal alps" OR "otway ranges" OR "otago hills" OR "orulgan ridge" OR "orulgan range" OR "ortler alps" OR "oregon coast range" OR "oregon cascades" OR "ore mts" OR "ordos plateau" OR "orange river valley" OR "old man range" OR "oku massif" OR "okanogan range" OR "obudu plateau" OR "oban hills" OR "nyika plateau" OR "nyenchen tanglha" OR "nyambene hills" OR "nyainqentanglha shan" OR "nuratau range" OR "nun kun" OR "nulato hills" OR "noyon uul" OR "northwestern pyrenees" OR "northwestern caucasus" OR "northwestern altai" OR "northwestern alps" OR "northwest adirondacks" OR "northern zagros" OR "northern wind river range" OR "northern western ghats" OR "northern wasatch range" OR "northern virginia blue ridge" OR "northern urals" OR "northern tyrolean limestone alps" OR "northern tablelands" OR "northern schwarzwald" OR "northern scandes" OR "northern sawatch range" OR "northern sangre de cristo range" OR "northern rockies" OR "northern ranges" OR "northern range" OR "northern pindus" OR "northern patagonian andes" OR "northern oregon coast range" OR "northern madison range" OR "northern karakorum" OR "northern greater khingan" OR "northern front ranges" OR "northern front range" OR "northern cumberland plateau" OR "northern california coast ranges" OR "northern blue ridge" OR "northern black hills" OR "northern black forest" OR "northern bitterroot range" OR "northern bakony" OR "northern appalachians" OR "northern apennines" OR "northern andes" OR "northern altai" OR "northern alps" OR "northern allegheny plateau" OR "northern adirondacks" OR "northern absaroka range" OR "northeastern pyrenees" OR "northeastern massif central" OR "northeastern dolomites" OR "northeastern altai" OR "northeastern alps" OR "north york moors" OR "north western alps" OR "north range" OR "north pennines" OR "north patagonian massif" OR "north pare" OR "north island volcanic plateau" OR "north eastern caucasus" OR "north eastern alps" OR "north central zagros" OR "north cascades" OR "north canterbury hills" OR "norra storfjället" OR "nopah range" OR "nizke tatry" OR "niut range" OR "nisling range" OR "nimba range" OR "nightcap range" OR "nicholas range" OR "ngong hills" OR "newton ridge" OR "newton range" OR "new zealand uplands" OR "new pass range" OR "new england uplands" OR "new england upland" OR "nepal himalaya" OR "néouvielle massif" OR "needle range" OR "nanling mts" OR "nanga parbat" OR "nandi hills" OR "nan shan" OR "nan ling" OR "namuli massif" OR "namanga hills" OR "nallamala hills" OR "nakhon si thammarat range" OR "nakai plateau" OR "naga hills" OR "myjava hills" OR "muzkol range" OR "musgrave ranges" OR "murchison range" OR "muránska planina" OR "muntanyes de prades" OR "mummy range" OR "mt trumbull" OR "mt thielsen" OR "mt shasta" OR "mt rogers" OR "mt robson" OR "mt rainier" OR "mt princeton" OR "mt olympus" OR "mt marcy" OR "mt kenya" OR "mt jefferson" OR "mt hood" OR "mt erciyes" OR "mt elgon" OR "mt daniel" OR "mt cameroon" OR "mt baldy" OR "mt ararat" OR "mt adams" OR "mount yale" OR "mount whitney" OR "mount washington" OR "mount trumbull" OR "mount tongariro" OR "mount tianmu" OR "mount thielsen" OR "mount tambora" OR "mount tai" OR "mount st* helens" OR "mount spokane" OR "mount shasta" OR "mount saint helens" OR "mount sabalan" OR "mount rundle" OR "mount ruapehu" OR "mount rogers" OR "mount robson" OR "mount rinjani" OR "mount rainier" OR "mount princeton" OR "mount pilchuck" OR "mount paiko" OR "mount ossa" OR "mount olympus" OR "mount nemrut" OR "mount namuli" OR "mount moosilauke" OR "mount meru" OR "mount meager" OR "mount massive" OR "mount marsabit" OR "mount marcy" OR "mount mansfield" OR "mount malindang" OR "mount lyell" OR "mount lofty ranges" OR "mount lebanon" OR "mount lebanon range" OR "mount lao" OR "mount kupe" OR "mount kulal" OR "mount kenya" OR "mount jefferson" OR "mount hood" OR "mount heng" OR "mount hasan" OR "mount harvard" OR "mount evans" OR "mount etna" OR "mount erciyes" OR "mount emei" OR "mount elgon" OR "mount elbert" OR "mount edith cavell" OR "mount drummond" OR "mount deception" OR "mount cook range" OR "mount chocorua" OR "mount cameroun" OR "mount cameroon" OR "mount cameron" OR "mount buffalo" OR "mount baldy" OR "mount athos" OR "mount assiniboine" OR "mount ararat" OR "mount aragats" OR "mount aix" OR "mount adams" OR "mosquito range" OR "moroccan atlas" OR "morgan range" OR "moravian-silesian beskids" OR "moorfoot hills" OR "monument peaks" OR "monts groulx" OR "monts faucilles" OR "monts du lyonnais" OR "monts du forez" OR "monts du cantal" OR "monts du beaujolais" OR "monts doré" OR "monts de saïda" OR "monts de lacaune" OR "monts de la madeleine" OR "monts de daïa" OR "monts d'arrée" OR "monti simbruini-ernici" OR "monti sicani" OR "monti sibillini" OR "monti sabini" OR "monti reatini" OR "monti prenestini" OR "monti picentini" OR "monti lepini" OR "monti lattari" OR "monti della laga" OR "monti della daunia" OR "monti carseolani" OR "montes vascos" OR "montes universales" OR "montes obarenes" OR "montes de vitoria" OR "montes de triano" OR "montes de toledo" OR "montes de maría" OR "montes de león" OR "montes de castejón" OR "monte ferru" OR "monte cristo range" OR "monte argentario" OR "monte arci" OR "monte albo" OR "montagnes noires" OR "montagnes françaises" OR "montagne pelée" OR "montagne noire" OR "montagne bourbonnaise" OR "mont péko" OR "mont blanc range" OR "mont blanc massif" OR "mono craters" OR "monitor range" OR "mongun taiga" OR "mongun taiga massif" OR "mongolian plateau" OR "mongolian altai" OR "monche tundra" OR "moldavian plateau" OR "mokra gora" OR "mogollon rim" OR "mitchell ranges" OR "mitchell range" OR "mission ridge" OR "mission range" OR "mishmi hills" OR "miri hills" OR "mineral mts" OR "mikir hills" OR "middle urals" OR "middle ural*" OR "middle atlas" OR "mid north coast" OR "michigan upper peninsula" OR "miao ling" OR "metaliferi mts" OR "meseta patagónica" OR "meseta de somuncurá" OR "meseta central" OR "mescal range" OR "mendip hills" OR "melville range" OR "meliau range" OR "mekongga mts" OR "medicine range" OR "mcpherson range" OR "mcilwraith range" OR "mccullough range" OR "mbooni hills" OR "mbeya range" OR "matobo hills" OR "mataketake range" OR "massive range" OR "massif du nord" OR "massif du carlit" OR "massif du canigou" OR "massif des bornes" OR "massif des arbailles" OR "massif des albères" OR "massif de l'arize" OR "massif de l'agly" OR "massif de la selle" OR "massif de la hotte" OR "massif central" OR "marojejy massif" OR "maritime alps" OR "marble range" OR "mantiqueira range" OR "maneau range" OR "mambilla plateau" OR "malvern hills" OR "malte brun range" OR "maligne range" OR "mala kapela" OR "makonde plateau" OR "major caucasus" OR "main ridge" OR "main range" OR "main caucasian range" OR "mahogany hills" OR "mahale mts" OR "mahadeo hills" OR "madison range" OR "madison plateau" OR "macizo nipe sagua baracoa" OR "macizo de teno" OR "macizo de somiedo" OR "macizo de posets" OR "macizo de montseny" OR "macizo de la maladeta" OR "macizo de cotiella" OR "macizo de anaga" OR "macizo colombiano" OR "maciço do itatiaia" OR "macgillycuddy's reeks" OR "macedon ranges" OR "macdonnell ranges" OR "lyngen alps" OR "lushai hills" OR "lu shan" OR "low tatras" OR "low tatra*" OR "low beskids" OR "low beskid*" OR "low beskid" OR "lovozero massif" OR "lost river range" OR "los montes de málaga" OR "los altos de chiapas" OR "lord range" OR "longwood range" OR "longshou shan" OR "longs peak" OR "longmen mts" OR "long shan" OR "loita hills" OR "lizard range" OR "livingstone range" OR "livingston range" OR "liverpool range" OR "little carpathians" OR "little beskids" OR "little atlas" OR "lisburne hills" OR "limestone ridge" OR "limestone massif" OR "ligurian prealps" OR "ligurian apennines" OR "ligurian alps" OR "lianhua shan" OR "leucite hills" OR "lesser khingan" OR "lesser himalaya" OR "lesser caucasus" OR "lepontine alps" OR "lenglong ling" OR "lena-angara plateau" OR "lena plateau" OR "lemhi range" OR "lely mts" OR "lechtal alps" OR "leaota mts" OR "lateral range" OR "last chance range" OR "lassen peak" OR "las vegas range" OR "large caucasus" OR "lao shan" OR "lammermuir hills" OR "lammermoor range" OR "lamb range" OR "lake range" OR "lake plateau" OR "laji shan" OR "ladakh range" OR "labwor hills" OR "la sila" OR "la serrota" OR "la serra d'en galceran" OR "kysucké beskydy" OR "kyrgyz range" OR "kyrgyz ala-too range" OR "kyrgyz ala too" OR "kwazulu natal midlands" OR "kuznetsk alatau" OR "kurai ridge" OR "kunlun range" OR "kunlun mts" OR "kungey alatau" OR "kun lun" OR "kumaun lesser himalayas" OR "kumaun himalaya" OR "kumaon himalaya*" OR "kular ridge" OR "kubor range" OR "krušné hory" OR "krupinská planina" OR "kroombit tops" OR "krivánska malá fatra" OR "kozie chrbty" OR "koryak range" OR "koppeh dagh" OR "kopet dagh" OR "kopet dag" OR "kongur shan" OR "kondoa irangi hills" OR "kondapalli hills" OR "kolyma upland" OR "kolvitsa massif" OR "kolli hills" OR "kokanee range" OR "kodar ridge" OR "kodar range" OR "kluane ranges" OR "kitchen range" OR "kirthar range" OR "kingston range" OR "khumbu himal" OR "khibiny massif" OR "khentei ridge" OR "khatipara ridge" OR "khasi hills" OR "kharaulakh range" OR "khangai mts" OR "keszthely mts" OR "kazakh uplands" OR "kawich range" OR "kaweka range" OR "kawdy plateau" OR "karpaty zachodnie" OR "karnataka plateau" OR "karlik range" OR "karen hills" OR "karatau ridge" OR "karatau range" OR "kangchenjunga himal" OR "kamnik–savinja alps" OR "kamnik alps" OR "kambui hills" OR "kamasia hills" OR "kalrayan hills" OR "kalba range" OR "kaimanawa range" OR "kaimai range" OR "kaikoura ranges" OR "kaibab plateau" OR "kachin hills" OR "jura mts" OR "juneau icefield" OR "julian prealps" OR "julian alps" OR "jos plateau" OR "jervois range" OR "jebel tuwaiq" OR "jebel sinjar" OR "jebel saghro" OR "jebel bishri" OR "jebel amour" OR "jebel akhdar" OR "jebal-e barez" OR "jebal barez" OR "jbel saghro" OR "jawadhu hills" OR "jawadhi hills" OR "javadi hills" OR "japanese alps" OR "james peak" OR "jaintia hills" OR "jabal sinjar" OR "jabal akhdar" OR "iveti hills" OR "italy range" OR "italian peaks" OR "italian group" OR "italian alps" OR "isalo massif" OR "iranian plateau" OR "inner western carpathians" OR "inland kaikoura range" OR "indian peaks" OR "indian peak range" OR "indian himalayan region" OR "idanre hills" OR "hunua ranges" OR "hume range" OR "humboldt range" OR "huila plateau" OR "house range" OR "hot springs range" OR "hot creek range" OR "horseranch range" OR "horse range" OR "horse heaven hills" OR "homathko icefield" OR "hokonui hills" OR "hohonu range" OR "hoggar massif" OR "hoback range" OR "hissar range" OR "hindu raj" OR "hindu kush" OR "hindu kush himalayan region" OR "hindu kush himalaya*" OR "hindu kush himalaya* region" OR "himachal himalayas" OR "hill country" OR "high tauern" OR "high tatras" OR "high tatra*" OR "high atlas" OR "high atlas range" OR "hidaka range" OR "herveys range" OR "hervey range" OR "herberton range" OR "henry mts" OR "hengduan shan" OR "heng shan" OR "heli shan" OR "helan shan" OR "helan range" OR "hayes range" OR "haut tell tunisien" OR "harz national park" OR "harts range" OR "hart ranges" OR "harrat al-sham" OR "harghita mts" OR "handies peak" OR "hamiguitan range" OR "hamersley range" OR "hall range" OR "haldon hills" OR "hakarimata range" OR "gurktal alps" OR "guayana shield" OR "gros ventre range" OR "grey range" OR "gregory rift" OR "greenwater range" OR "greenhorn range" OR "greater khingan" OR "greater caucasus" OR "great western tiers" OR "great rift valley" OR "great range" OR "great escarpment" OR "great dividing range" OR "great basin ranges" OR "grays range" OR "grays peak" OR "grays hills" OR "gray hills" OR "gravelly range" OR "grant range" OR "granite range" OR "grands causses" OR "grandes rousses" OR "grand mesa" OR "grand canyon" OR "gran sasso" OR "gran paradiso" OR "grampian range" OR "graian alps" OR "gore range" OR "gorce mts" OR "gold range" OR "goat rocks" OR "goat range" OR "glens of antrim" OR "glenlyon range" OR "glarus alps" OR "glacier peak" OR "gissar range" OR "gillis range" OR "gibraltar range" OR "german alps" OR "german alpine foreland" OR "gerecse mts" OR "georgian caucasus" OR "georgia blue ridge" OR "gelibolu yarimadasi" OR "gawler ranges" OR "gausdal vestfjell" OR "garo hills" OR "garnet range" OR "garhwal himalaya" OR "garfield peaks" OR "garfield hills" OR "gaoligong shan" OR "gaoligong mts" OR "gangdise range" OR "ganesh himal" OR "gammon ranges" OR "gallatin range" OR "gailtal alps" OR "gabilan range" OR "gabbs valley range" OR "fuuta jaloo" OR "futa jallon" OR "futa djalon" OR "fuentes carrionas" OR "fruška gora" OR "front range" OR "frenchman hills" OR "french pyrenees" OR "french jura" OR "french alps" OR "fraser plateau" OR "franconian jura" OR "fra cristobal range" OR "fox range" OR "fouta djallon" OR "flint hills" OR "flint creek range" OR "flinders range" OR "flathead range" OR "flat tops" OR "fish springs range" OR "fish lake plateau" OR "fish creek range" OR "first range" OR "finisterre range" OR "fila brunqueña" OR "fergana range" OR "fennoscandian shield" OR "fell lapland" OR "fawnie range" OR "farallones de cali" OR "falaise de banfora" OR "fairweather range" OR "făgăraș mts" OR "everard ranges" OR "european upland*" OR "european alps" OR "etna volcan*" OR "etna mount*" OR "epi island" OR "ely range" OR "elk ridge" OR "elk range" OR "elgeyo escarpment" OR "elbow range" OR "el garraf" OR "eje neovolcanico mexicano" OR "egan range" OR "edwards plateau" OR "edough massif" OR "ecuadorian andes" OR "eastern tian shan" OR "eastern tatras" OR "eastern tannu ola" OR "eastern sudetes" OR "eastern sayan" OR "eastern satpura range" OR "eastern rift" OR "eastern ridge" OR "eastern rhodopes" OR "eastern range" OR "eastern pyrenees" OR "eastern pre-pyrenees" OR "eastern pamirs" OR "eastern middle atlas" OR "eastern main range" OR "eastern kunlun" OR "eastern hindu kush" OR "eastern hills" OR "eastern high atlas" OR "eastern hellenides" OR "eastern hajar" OR "eastern ghat" OR "eastern ghats" OR "eastern escarpment" OR "eastern coastal range" OR "eastern caucasus" OR "eastern carpathians" OR "eastern brooks range" OR "eastern betics" OR "eastern beskids" OR "eastern arc mts" OR "eastern anti-atlas" OR "eastern alps" OR "eastern adirondacks" OR "east* carpathian*" OR "east usambara" OR "east range" OR "east kunlun" OR "east humboldt range" OR "east african plateau" OR "dzungarian alatau" OR "dzhugdzhur range" OR "dugway range" OR "duck creek range" OR "dry andes" OR "drummond range" OR "douglas ranges" OR "douglas range" OR "dorsale tunisienne" OR "dorsal gallega" OR "domeyko range" OR "dolomitic alps" OR "dolomiti lucane" OR "dogtooth range" OR "dobrogea plateau" OR "djebel sinjar" OR "djebel amour" OR "dinaric alps" OR "dinara massif" OR "dieng volcanic complex" OR "dieng plateau" OR "diamond range" OR "dial range" OR "diablo range" OR "dhaulagiri himalaya" OR "dhauladhar range" OR "dévoluy massif" OR "deserters range" OR "desert range" OR "deseado massif" OR "deosai plateau" OR "deli jovan" OR "defiance plateau" OR "deep creek range" OR "deccan plateau" OR "dean hill" OR "daxue shan" OR "dawson range" OR "darvaz range" OR "darling range" OR "daqing shan" OR "danghe nanshan" OR "danghe nan shan" OR "dandenong ranges" OR "dafla hills" OR "dachstein mts" OR "dabie mts" OR "daba shan" OR "da hinggan" OR "da hinggan range" OR "da hinggan ling" OR "da ba shan" OR "czech carpathians" OR "cypress hills" OR "cumbres calchaquíes" OR "cumberland plateau" OR "culebra range" OR "crocker range" OR "crimean mts" OR "crawford range" OR "craigieburn range" OR "cottian alps" OR "cordón del portillo" OR "cordón del plata" OR "conondale range" OR "confusion range" OR "columbia plateau" OR "columbia icefield" OR "columbia hills" OR "colorado rockies" OR "colorado plateau" OR "coconino plateau" OR "coast range" OR "cleopatra's needle" OR "clent hills" OR "clemenceau icefield" OR "clear range" OR "clear hills" OR "clayton ridge" OR "clark range" OR "cimarron range" OR "chyulu hills" OR "churia hills" OR "chulyshman plateau" OR "chukotka upland" OR "chugach range" OR "chota nagpur plateau" OR "chola shan" OR "choč mts" OR "chittagong hill tracts" OR "chiricahua peak" OR "chinese wall" OR "chinese loess plateau" OR "chin hills" OR "chimantá massif" OR "chilkat range" OR "chilcotin ranges" OR "chilcotin plateau" OR "chiemgau alps" OR "chichester ranges" OR "cheviot hills" OR "chersky ridge" OR "chersky range" OR "cherry creek range" OR "cherang'any hills" OR "chatkal range" OR "chartreuse massif" OR "chapada dos veadeiros" OR "chapada dos guimarães" OR "chapada do araripe" OR "chapada diamantina" OR "chapada das mesas" OR "chapada das mangabeiras" OR "changbai mts" OR "chaîne des puys" OR "chaîne des matheux" OR "chagai hills" OR "cerros orientales" OR "cerros del sira" OR "cerros de escazú" OR "cerros colorados" OR "cerro guaiquinima" OR "cerro duida" OR "centre hills" OR "central-eastern alps" OR "central wind river range" OR "central western ghats" OR "central wasatch range" OR "central volcanic zone" OR "central venezuelan andes" OR "central uplands" OR "central tian shan" OR "central tell atlas" OR "central taurus" OR "central tablelands" OR "central sudetes" OR "central southern pyrenees" OR "central siberian plateau" OR "central ranges" OR "central range" OR "central pyrenees" OR "central pre-pyrenees" OR "central plateau" OR "central pindus" OR "central oregon coast range" OR "central north caucasus" OR "central moravian carpathians" OR "central middle atlas" OR "central mexican plateau" OR "central massif" OR "central massif central" OR "central madison range" OR "central lesser himalaya*" OR "central kunlun" OR "central japanese alps" OR "central himalaya*" OR "central high plateau" OR "central high atlas" OR "central front ranges" OR "central front range" OR "central cumberland plateau" OR "central caucasus" OR "central betics" OR "central balkan mts" OR "central appalachians" OR "central apennines" OR "central anti-atlas" OR "central andes" OR "central america volcanic arc" OR "central alps" OR "central alborz mts" OR "centennial range" OR "ceahlău massif" OR "causses du quercy" OR "caucasus mts" OR "caucasus major" OR "cathedral range" OR "cathedral group" OR "catena costiera" OR "catalan coastal range" OR "cascade* range" OR "cascade range" OR "carter range" OR "carson range" OR "carso huasteco" OR "carpathian mts" OR "carnic prealps" OR "carnic alps" OR "cariboo plateau" OR "cardamom mts" OR "cardamom hills" OR "capitol reef" OR "capitol hills" OR "cape midlands" OR "canyon ranges" OR "cantabrian range" OR "cantabrian mts" OR "candelaria hills" OR "canadian shield" OR "canadian rockies" OR "canadian appalachians" OR "campbell range" OR "campbell hills" OR "campanian apennines" OR "cameroon line" OR "camelsfoot range" OR "călimani mts" OR "california coast ranges" OR "california cascades" OR "calar del río mundo" OR "calabrian apennines" OR "burster range" OR "bureya massif" OR "bungo range" OR "bullfrog hills" OR "bukit tigapuluh" OR "bukit barisan" OR "buffalo hills" OR "buda hills" OR "buckskin range" OR "bucegi mts" OR "browning range" OR "brooks range" OR "broken hills" OR "brindabella range" OR "bridger range" OR "brenta group" OR "brecon beacons" OR "brazilian plateau" OR "brazeau range" OR "boz dag*" OR "bowland fells" OR "bow range" OR "boundary ranges" OR "bosche range" OR "bornes massif" OR "border ranges" OR "borborema plateau" OR "book cliffs" OR "bong range" OR "bonar range" OR "bomi hills" OR "bolu massif" OR "bolsón de mapimí" OR "bolivian plateau" OR "bolivian andes" OR "bolaven volcanic field" OR "bolaven plateau" OR "bohemian massif" OR "bohemian forest" OR "bohea hills" OR "bogotá savanna" OR "bogong high plains" OR "bogda shan" OR "bogd uul" OR "blue range" OR "blue mts" OR "bloods range" OR "blidean atlas" OR "blackwater range" OR "blackall range" OR "black rock range" OR "black range" OR "black hills" OR "black forest" OR "black divide" OR "biu plateau" OR "bitterroot range" OR "bismarck range" OR "binalud range" OR "bihor mts" OR "bighorn range" OR "big salmon range" OR "big range" OR "big caucasus" OR "bhutan himalaya" OR "betic system" OR "betic chain" OR "baetic system" OR "baetic chain" OR "beskid sadecki" OR "bernina massif" OR "bernese alps" OR "berkshire hills" OR "bergamo alps" OR "berchtesgaden alps" OR "benton range" OR "bendor range" OR "ben ohau range" OR "belted range" OR "bellenden ker range" OR "belledonne massif" OR "belianske tatras" OR "bei shan" OR "bayuda volcanic field" OR "bayan har shan" OR "bavarian forest" OR "bavarian alps" OR "bauges massif" OR "bateke plateau" OR "barrington tops" OR "barrier range" OR "barnett hills" OR "barisan range" OR "barguzin ridge" OR "barguzin range" OR "barail range" OR "bannock range" OR "banjaran crocker" OR "bandiagara escarpment" OR "banded range" OR "bamenda grassfields" OR "baltic shield" OR "ball range" OR "balkan range*" OR "balkan mts" OR "baldy peak" OR "balaton uplands" OR "bakossi mts" OR "bailadila range" OR "baikal ridge" OR "baikal range*" OR "baetic range*" OR "betic range*" OR "badzhal range" OR "backbone ranges" OR "baba budan hills" OR "austrian central alps" OR "austrian alps" OR "australian alps" OR "aurora range" OR "atlas range*" OR "atlas marocain" OR "atlas chain*" OR "atlas blidéen" OR "atlantic plateau" OR "atewa range" OR "atbashi range" OR "asturian massif" OR "assam himalaya" OR "aspen range" OR "arthur range" OR "arrowsmith range" OR "arrow canyon range" OR "armorican massif" OR "arjin shan" OR "argentinian andes" OR "arctic ocean islands" OR "aravalli range" OR "aravali range" OR "araucaria plateau" OR "arakan yoma" OR "arakan hills" OR "aragonese pyrenees" OR "aquarius plateau" OR "apuseni mts" OR "apuan alps" OR "aprada tepui" OR "appalachian* range" OR "appalachian ridges" OR "appalachian plateaus" OR "aorangi range" OR "anvil range" OR "antrim plateau" OR "anti taurus" OR "antelope range" OR "annapurna range" OR "annapurna himalaya" OR "ankaratra massif" OR "angolan escarpment" OR "andringitra massif" OR "andes peruanos" OR "andes fueguinos" OR "andean plateau" OR "anatolian plateau" OR "anaimalai hills" OR "anaconda range" OR "anabar plateau" OR "amne machin" OR "ammergauer alpen" OR "ammergau alps" OR "alxa plateau" OR "altyn tagh" OR "altun shan" OR "altun mts" OR "altos de jalisco" OR "altiplano cundiboyacense" OR "altay sayan" OR "altay range" OR "altai sayan" OR "altai range" OR "altai mts" OR "alpine-himalayan orogenic belt" OR "alpine-himalayan belt" OR "alpine lakes area" OR "alpide belt" OR "alpi orobie" OR "alpes maritimes" OR "almăj depression" OR "allies group" OR "allgäu alps" OR "allegheny plateau" OR "allegheny front" OR "alishan national scenic area" OR "algerian atlas" OR "alexander range" OR "alexander archipelago" OR "aleutian ridge" OR "aleutian range" OR "aleutian arc" OR "aleut* islands" OR "aldan plateau" OR "alborz mts" OR "albion range" OR "alaska range" OR "alashan plateau" OR "ajodhya hills" OR "ailao shan" OR "ailao mts" OR "aiguilles rouges" OR "aiguilles rouges massif" OR "aibga ridge" OR "ahaggar mts" OR "adrar des ifoghas" OR "adobe range" OR "adirondack mts" OR "adirondack high peaks" OR "adelbert range" OR "adamawa plateau" OR "adamawa massif" OR "absaroka range" OR "abruzzo apennine*" OR "abruzzi apennines" OR "aberdare range" OR "jebel tessala" OR rockies OR andes OR betics OR himalaya OR appalachian* OR andean OR caucasus OR urals OR carpathians OR himachal OR apennine* OR apennines OR pyrenees OR jura OR tianshan OR qinling OR adirondacks OR altiplano OR zagros OR ardennes OR vosges OR kunlun OR babor* OR kilimanjaro OR witwatersrand OR tatras OR alborz OR palisades OR vesuvius OR harz OR nilgiris OR karakoram OR guadarrama OR qiangtang OR pennines OR anti-atlas OR vercors OR beishan OR cévennes OR grampians OR sudetes OR morvan OR sikhote-alin OR dinarides OR drakensberg OR schwarzwald OR teide OR karpaty OR karakorum OR fiordland OR beskids OR nanshan OR ruapehu OR altyn-tagh OR pieniny OR velebit OR uludağ OR canyonlands OR cotswolds OR waterberg OR katahdin OR jinggangshan OR laoshan OR rondane OR hindukush OR vanoise OR emmental OR cairngorms OR taunus OR kohala OR medvednica OR odenwald OR pilat OR adamello OR tatry OR pollino OR ecrins OR pirin OR gorce OR hunsrück OR yinshan OR mecsek OR hellenides OR sabalan OR trans-himalaya OR djurdjura OR alay OR arves OR majella OR ruwenzori OR jirisan OR aubrac OR belledonne OR snowdonia OR breheimen OR jotunheimen OR bakony OR madonie OR burren OR daqingshan OR udzungwa OR sauerland OR soutpansberg OR spessart OR burda OR scandes OR chablais OR katla OR matese OR nebrodi OR papuk OR alishan OR hardangervidda OR pelion OR cassiars OR fenghuangshan OR monfragüe OR aspromonte OR westerwald OR kopaonik OR cederberg OR brandberg OR vogelsberg OR cangshan OR gorongosa OR nemrut OR nassa OR gaoligongshan OR sahand OR taillefer OR kalnik OR tienshan OR mengshan OR biokovo OR tjörnes OR nyainqentanglha OR dahra OR svartisen OR parnassos OR dayaoshan OR karadağ OR gallot OR vitosha OR pamir-alai OR mátra OR pilanesberg OR ségala OR pre-pyrenees OR kebnekaise OR blouberg OR hualalai OR edough OR rooiberg OR korab OR vourinos OR karadag OR sumava OR putorana OR elburz OR tafelberg OR okstindan OR durmitor OR valdres OR verno OR appennini OR donggan OR bozburun OR behistun OR učka OR xueshan OR yaoshan OR bibans OR khamar-daban OR karasjok OR winterberg OR alburni OR chornohora OR cézallier OR baviaanskloof OR planèze OR changthang OR tsinling OR dovrefjell OR giffre OR cedarberg OR jesenik OR kozjak OR warrumbungles OR jablanica OR gennargentu OR changtang OR zarkos OR kopetdag OR crestones OR magaliesberg OR pamir-alay OR langeberg OR lauzière OR daliangshan OR andringitra OR funiushan OR ukaguru OR transhimalaya OR gesäuse OR sidobre OR ozren OR presanella OR gariwerd OR svilaja OR macelj OR beaufortain OR prokletije OR fongen OR krndija OR finnmarksvidda OR dilj OR mariovo OR psunj OR swartruggens OR jastrebac OR ankaratra OR hardangerjøkulen OR karwendel OR anti-lebanon OR beni-chougrane OR branisko OR tribeč OR piketberg OR stolowe OR yamanlar OR kithairon OR parnitha OR poľana OR pratomagno OR slavyanka OR mosor OR mavrovouni OR titteri OR atakor OR galičica OR alburz OR brownsberg OR grammos OR hochschwab OR mainpat OR saltfjellet OR artense OR rtanj OR rätikon OR chataigneraie OR auyantepui OR rumija OR kasikasima OR wolkberg OR ograzhden OR drauzug OR mianshan OR majevica OR svydovets OR chitteri OR chřiby OR žiar OR anyemaqen OR tavan-bogd OR gantrisch OR lovćen OR bleia OR forollhogna OR tomaros OR gasherbrum OR lévézou OR storskarven OR haukelifjell OR aladaglar OR khibins OR trollheimen OR kvamshesten OR nordfjella OR daxueshan OR sinjajevina OR beljanica OR fangdoushan OR kožuf OR hualālai OR tsambagarav OR jinpingshan OR diamir OR jakupica OR sarisariñama OR qangtang OR tzoumerka OR vindelfjällen OR elborz OR golak OR escandorgue OR xerovouni OR ryfylkeheiane OR ljubišnja OR carpati OR aladagh OR malundwe OR orjen OR sunndalsfjella OR drakensberge OR waifangshan OR sausfjellet OR javorie OR jiulingshan OR ottadalen OR kukavica OR anysberg OR suurberg OR blåfjella OR kugitangtau OR hamar-daban OR norefjell OR vlahina OR gruvefjellet OR tsagan-daban OR filefjell OR romsdalsalpane OR stølsheimen OR sokoró OR maleshevo OR yengisogat OR babatag OR antichasia OR anti-apennines OR karawanks OR ammergebirge OR cserhat OR toven OR långfjället OR goljak OR blefjell OR bogdashan OR erimanthos OR skjækerfjella OR høgtuva OR ålfotbreen OR meygal OR borzsony OR ambohiby OR kharkhiraa OR charchiraa OR murovdag OR steenkampsberg OR tsinling-dabashan OR jurjura OR kjølhaugan OR orvilos OR valamara OR pentadactylos OR plješivica OR artfjället OR roggeveldberge OR tendurek OR skaget OR ilfjellet OR trysilfjellet OR pothigai OR slievenamon OR maumturks OR kamakou OR vtacnik OR jabllanicë OR jundushan)

OR

AK=(alpine OR subalpine OR montane OR submontane OR mountain OR mountainous OR upland OR highland OR "high* elevation" OR "high* altitude" OR summit OR yunga OR puna OR paramo OR glacial OR himalayan OR carpathian OR caucasian OR appalachian OR cordilleran OR pyrenean OR tibetan OR cantabrian OR altiplano OR afromontane OR afroalpine OR madrean OR paramo OR yunga OR puna OR upland OR "eastern arc" OR "mid* elevation" OR "mid* altitude" OR cantabrian OR pontic OR glacial OR summit OR volcanic OR crater OR paramo$ OR páramo$ OR subkryal OR "sub kryal" OR tarn OR "glacial pond$" OR "glacier fed aquatic" OR "glacier fed catchment$" OR "glacier fed channel$" OR "glacier fed pond$" OR "glacier fed spring$" OR "glacier fed water$" OR "thaw pond$" OR "mountain torrent$" OR "altitud* decline" OR "altitud* decrease" OR "altitud* distribution$" OR "altitud* diversit*" OR "altitud* gradient$" OR "altitud* increase" OR "altitud* limit$" OR "altitud* pattern$" OR "altitud* range$" OR "altitud* shift$" OR "altitud* transect$" OR "altitud* variabilit*" OR "altitud* variation$" OR "altitud* zonation$" OR "campo* rupestre$" OR "cryo oromediterranean" OR "elevation* decline" OR "elevation* decrease" OR "elevation* distribution$" OR "elevation* diversit*" OR "elevation* gradient$" OR "elevation* increase" OR "elevation* limit$" OR "elevation* pattern$" OR "elevation* range$" OR "elevation* shift$" OR "elevation* transect$" OR "elevation* variabilit*" OR "elevation* variation$" OR "elevation* zonation$" OR "glacial retreat$" OR "gradient$ of altitude" OR "gradient$ of elevation" OR "hautes terres" OR "high* altitude$" OR "high* elevation$" OR "rock glacier$" OR "sky island$" OR "tropical glacier$" OR afroalpine OR "afro alpine" OR afromontane OR "afro montane" OR alpine OR alpino$ OR altiplano$ OR banjaran OR cordilheira$ OR cordill?re$ OR cordillera$ OR daglar OR daglari OR fjell OR fjellkjede OR gebirge OR gunung OR highland$ OR khrebet OR montane OR mountain$ OR mountainous OR mountaintop$ OR nunatak$ OR pegunungan OR planina OR pohorie OR serra$ OR serralada$ OR sierra$ OR stratovolcano* OR "strato volcano*" OR subalpine OR submontane OR "sub montane" OR upland$ OR volcano* OR "glacier outflow$" OR glaciolacustrine OR "glacio lacustrine" OR submountainous OR "sub mountainous" OR "żywiec beskids" OR "žumberak mts" OR "zomba plateau" OR "zillertal alps" OR "zarafshan range" OR "zanskar range" OR "zane hills" OR "zagros mts" OR "yunnan-guizhou plateau" OR "yungui plateau" OR "yukon-tanana uplands" OR "yu shan" OR "yorkshire dales" OR "yi shan" OR "yenisei ridge" OR "yenisei range" OR "yellowstone plateau" OR "yangming shan" OR "xue shan" OR "xiong'er shan" OR "xiao shan" OR "xiao hinggan ling" OR "x hills" OR "wyoming range" OR "wudang shan" OR "wooley range" OR "wind river range" OR "willapa hills" OR "wild horse range" OR "wieliczka foothills" OR "wichita mts" OR "whitefish range" OR "white river plateau" OR "white range" OR "white pine range" OR "white mts" OR "white divide" OR "white carpathians" OR "whistler range" OR "western venezuelan andes" OR "western tian shan" OR "western taurus" OR "western tatras" OR "western tatra*" OR "western tannu ola" OR "western sudetes" OR "western sayan" OR "western sahara atlas" OR "western romanian carpathians" OR "western rhodopes" OR "western range" OR "western pyrenees" OR "western pamirs" OR "western pacific islands" OR "western middle atlas" OR "western main range" OR "western lesser himalaya*" OR "western kunlun" OR "western hindu kush" OR "western himalaya*" OR "western high atlas" OR "western hellenides" OR "western hajar" OR "western ghat" OR "western ghats" OR "western foothills" OR "western caucasus" OR "western carpathians" OR "western bieszczady" OR "western betics" OR "western beskids" OR "western balkan mts" OR "western anti-atlas" OR "western alps" OR "west* carpathian*" OR "west* african uplands" OR "west usambara" OR "west range" OR "west kunlun" OR "west coast ranges" OR "west coast range" OR "wenatchee ridge" OR "wellington range" OR "weepah hills" OR "webster range" OR "wassuk range" OR "washakie range" OR "wasatch range" OR "walker range" OR "walden ridge" OR "waitakere ranges" OR "waianae range" OR "vosges mts" OR "voras mts" OR "volga uplands" OR "volcanic hills" OR "volcanes de colima" OR "vitim plateau" OR "virginia range" OR "villány mts" OR "victorian alps" OR "victoria range" OR "vértes hills" OR "vermilion range" OR "verkhoyansk ridge" OR "verkhoyansk range" OR "vercors massif" OR "venezuelan coastal range" OR "venezuelan andes" OR "venetian prealps" OR "velka fatra" OR "velika kapela" OR "velence hills" OR "vanoise massif" OR "valais alps" OR "val di non" OR "usun apau plateau" OR "usambara mts" OR "ural mts" OR "upper atlas" OR "united states range" OR "umbrian apennines" OR "ulugh muztagh" OR "ulagan plateau" OR "ulaan taiga" OR "ukrainian carpathians" OR "uinkaret plateau" OR "udokan ridge" OR "udokan range" OR "tyan shan" OR "two thumb range" OR "twin range" OR "tuscan–emilian apennines" OR "turkestan range" OR "tunka ridge" OR "tunisian dorsale" OR "tunisian atlas" OR "tunguska plateau" OR "tukuringra ridge" OR "tukuringra range" OR "tug hill" OR "tsingy de bemaraha" OR "tsambagarav ridge" OR "truong son range" OR "truckee range" OR "tropical andes" OR "trinity range" OR "trinity alps" OR "trialeti range" OR "trara* mount*" OR "transylvanian plateau" OR "transylvanian plain" OR "transverse ranges" OR "trans-mexican volcanic belt" OR "trans-ili alatau" OR "tosco romagnolo apennines" OR "torlesse range" OR "toquima range" OR "tongbai shan" OR "tomkinson ranges" OR "tokaj mts" OR "toiyabe range" OR "tobin range" OR "tobacco range" OR "toano range" OR "timpahute range" OR "tigray plateau" OR "tien shan" OR "tibetan plateau" OR "tianmu shan" OR "tian shan" OR "thuringian forest" OR "thompson peaks" OR "thompson peak" OR "thomas range" OR "thirtymile range" OR "the sperrins" OR "the snowies" OR "the maniototo" OR "the hunter's hills" OR "the hokonuis" OR "the catlins" OR "the ancient wall" OR "teton range" OR "tethyan orogenic belt" OR "terskey ala-too" OR "termit massif" OR "terminal range" OR "tenmile range" OR "temblor range" OR "tell atlas" OR "telangana plateau" OR "te urewera" OR "tchabal mbabo" OR "tawau hills" OR "taurus range" OR "taurus mts" OR "tatras mts" OR "tassili n'ajjer" OR "taringatura hills" OR "tararua range" OR "tannu ola" OR "tanggula mts" OR "talkeetna mts" OR "talas alatau" OR "taita hills" OR "taillefer massif" OR "taihang shan" OR "tai shan" OR "tadrart acacus" OR "système ibérique" OR "synnyr massif" OR "swiss prealps" OR "swiss jura" OR "swiss alps" OR "świętokrzyskie mts" OR "sweetgrass hills" OR "swannell ranges" OR "swan range" OR "swabian jura" OR "suva planina" OR "surrey hills" OR "superior upland" OR "suntar-khayata ridge" OR "suntar-khayata range" OR "summit ridge" OR "sumava national park" OR "sulphur spring range" OR "suisse normande" OR "submeseta sur" OR "submeseta norte" OR "sublette range" OR "sublett range" OR "stubai alps" OR "stuart range" OR "strzelecki ranges" OR "strathbogie ranges" OR "storm ridge" OR "stolowe mts" OR "stirling range" OR "stillwater range" OR "stikine plateau" OR "stauning alps" OR "stara planina" OR "stanovoy range" OR "stanovik range" OR "stanford range" OR "sredna gora" OR "sredinny range" OR "spotted range" OR "spectrum range" OR "specter range" OR "spatsizi plateau" OR "spanish pyrenees" OR "spanish peaks" OR "southwestern pyrenees" OR "southwestern massif central" OR "southwestern alps" OR "southland hills" OR "southern zagros" OR "southern wind river range" OR "southern western ghats" OR "southern wasatch range" OR "southern urals" OR "southern ural*" OR "southern uplands" OR "southern schwarzwald" OR "southern scandes" OR "southern sawatch range" OR "southern rockies" OR "southern rhodopes" OR "southern range" OR "southern peaks" OR "southern patagonian andes" OR "southern oregon coast range" OR "southern oregon cascades" OR "southern new hampshire" OR "southern limestone alps" OR "southern japanese alps" OR "southern great escarpment" OR "southern cumberland plateau" OR "southern catskills" OR "southern carpathians" OR "southern california coast ranges" OR "southern calcareous alps" OR "southern black hills" OR "southern black forest" OR "southern bakony" OR "southern aravalli range" OR "southern appalachians" OR "southern appalachian ridges" OR "southern apennines" OR "southern andes" OR "southern alps" OR "southern african ranges" OR "southern adirondacks" OR "southeastern pyrenees" OR "southeastern massif central" OR "southeastern alps" OR "south western caucasus" OR "south western alps" OR "south pennines" OR "south pare" OR "south pahroc range" OR "south eastern caucasus" OR "south eastern alps" OR "south downs" OR "south altai" OR "soufrière hills" OR "sonoma range" OR "song shan" OR "somali plateau" OR "solution range" OR "solitude range" OR "snowy range" OR "snowcrest range" OR "snake river range" OR "snake range" OR "slovenian alps" OR "slovak paradise" OR "slovak karst" OR "slovak carpathians" OR "slate range" OR "slate mts" OR "skopska crna gora" OR "skippers range" OR "siwalik range" OR "sitheri hills" OR "sistema periférico" OR "sistema mediterráneo catalán" OR "sistema ibérico" OR "sistema de tandilia" OR "sistema central" OR "sistema bético" OR "sirumalai hills" OR "sirente velino" OR "singatse range" OR "simpson park range" OR "simi hills" OR "silvretta massif" OR "silver peaks range" OR "silver peak range" OR "silesian foothills" OR "silesian beskids" OR "sila piccola" OR "sila greca" OR "sila grande" OR "sikkim himalayas" OR "sifton ranges" OR "shoshone range" OR "shiwan dashan" OR "shivwits plateau" OR "shimba hills" OR "sheep range" OR "sheep creek range" OR "shan hills" OR "shaler range" OR "seymour range" OR "sevier plateau" OR "sette daban ridge" OR "sette daban range" OR "setesdal vesthei" OR "setesdal ryfylke" OR "servarayan hills" OR "serres de llevant" OR "serranías chiquitanas" OR "serranía del litoral" OR "serranía del interior" OR "serranía del aguaragüe" OR "serrania de ronda" OR "serranía de pirre" OR "serranía de macuira" OR "serranía de los yariguíes" OR "serranía de los paraguas" OR "serranía de los churumbelos" OR "serranía de las quinchas" OR "serranía de cuenca" OR "serranía de chiribiquete" OR "serra parima" OR "serra negra" OR "serra mariola" OR "serra jeci" OR "serra grande" OR "serra geral" OR "serra geral de goiás" OR "serra gaúcha" OR "serra dourada" OR "serra dos tapes" OR "serra dos pirineus" OR "serra dos órgãos" OR "serra dos cristais" OR "serra dos cocais" OR "serra dos cavalos" OR "serra dos carajás" OR "serra dos aimorés" OR "serra do xistral" OR "serra do tombador" OR "serra do tabuleiro" OR "serra do suído" OR "serra do sincorá" OR "serra do rio do rastro" OR "serra do ramalho" OR "serra do papagaio" OR "serra do moco" OR "serra do marão" OR "serra do mar" OR "serra do lajeado" OR "serra do gandarela" OR "serra do estrondo" OR "serra do espinhaço" OR "serra do espigão" OR "serra do divisor" OR "serra do courel" OR "serra do cipó" OR "serra do caparaó" OR "serra do caldeirão" OR "serra do cachimbo" OR "serra do cabral" OR "serra d'irta" OR "serra d'espadà" OR "serra del montsant" OR "serra del catllaràs" OR "serra del cadí" OR "serra de uruburetama" OR "serra de são miguel" OR "serra de são mamede" OR "serra de santana" OR "serra de sant gervàs" OR "serra de paranapiacaba" OR "serra de montemuro" OR "serra de montejunto" OR "serra de monchique" OR "serra de maracaju" OR "serra de les gavarres" OR "serra de joão do vale" OR "serra de gerês" OR "serra de botucatu" OR "serra de baturité" OR "serra das matas" OR "serra das confusões" OR "serra das araras" OR "serra da saudade" OR "serra da peneda" OR "serra da nogueira" OR "serra da neve" OR "serra da mocidade" OR "serra da meruoca" OR "serra da mantiqueira" OR "serra da malcata" OR "serra da leba" OR "serra da jacobina" OR "serra da graciosa" OR "serra da estrela" OR "serra da capivara" OR "serra da capelada" OR "serra da cantareira" OR "serra da canastra" OR "serra da borda" OR "serra da bodoquena" OR "serra da bocaina" OR "serra da aratanha" OR "serra calderona" OR "serra azul" OR "serbian carpathians" OR "sentinel range" OR "sentinel hills" OR "selwyn range" OR "selennyakh ridge" OR "selawik hills" OR "selamiut range" OR "seaward kaikoura range" OR "seaman range" OR "scotchman peak*" OR "schrader range" OR "schell creek range" OR "scenic rim" OR "sayan mts" OR "sawtooth ridge" OR "sawtooth range" OR "sawatch range" OR "saur ridge" OR "satpura range" OR "sarntal alps" OR "sarek mts" OR "santa lucia range" OR "sandwich range" OR "sandhills range" OR "sand springs range" OR "san rafael swell" OR "san juan mts" OR "san francisco peaks" OR "salt river range" OR "salt range" OR "salair ridge" OR "sacramento mts" OR "sabana de bogotá" OR "russian caucasus" OR "russian altai" OR "rumpi hills" OR "rudnyi altai" OR "ruby range" OR "ruahine range" OR "royston hills" OR "royal group" OR "rough ridge" OR "romanian carpathians" OR "rolwaling himal" OR "rocky range" OR "rocky mts" OR "rock and pillar range" OR "robinson ranges" OR "robertson range" OR "roan plateau" OR "roan cliffs" OR "river mts" OR "ritter range" OR "rimutaka range" OR "rila mts" OR "rila geopark" OR "richmond range" OR "richmond hills" OR "rhodope mts" OR "rhenish massif" OR "rewa plateau" OR "reveille range" OR "retezat mts" OR "resting spring range" OR "redcloud peak" OR "red sea hills" OR "red hills" OR "raukumara range" OR "rattlesnake hills" OR "raton mesas" OR "range of the shelter" OR "rampart range" OR "ram range" OR "rakwana hills" OR "rakhine yoma" OR "rakaposhi range" OR "rajmahal hills" OR "raja ampat islands" OR "rainbow range" OR "ragged range" OR "quinn canyon range" OR "queen elizabeth range" OR "quantock hills" OR "qinling dabashan" OR "qingzang gaoyuan" OR "qinghai nanshan" OR "qin ling" OR "qilian mts" OR "qian shan" OR "qaidam basin" OR "pyrenees range" OR "putu range" OR "putorana plateau" OR "purcell range" OR "puna de atacama" OR "puertos de beceite" OR "provence alps" OR "price range" OR "presidential range" OR "president range" OR "prealpi giulie" OR "prealpi bergamasche" OR "préalpes de nice" OR "požeška gora" OR "považský inovec" OR "pouakai range" OR "portneuf range" OR "porcupine hills" OR "polonyna borzhava" OR "polish carpathians" OR "polar urals" OR "polar ural*" OR "polar range" OR "pohronský inovec" OR "pocono plateau" OR "po hills" OR "pliny range" OR "plateau of tibet" OR "plateau of mozambique" OR "plateau de millevaches" OR "plateau de danyi" OR "planalto da borborema" OR "planalto brasileiro" OR "piute range" OR "piton des neiges" OR "piton de la fournaise" OR "pisa range" OR "pir panjal range" OR "pioneer range" OR "pintler range" OR "pine grove hills" OR "pine forest range" OR "pilot range" OR "pilot butte" OR "pikikiruna range" OR "picos de europa" OR "pico island" OR "pico da neblina" OR "petermann ranges" OR "peruvian andes" OR "persian plateau" OR "pennine alps" OR "peninsular ranges" OR "peñas de cervera" OR "peloritani range" OR "pekulney range" OR "pegu yoma" OR "pavant range" OR "patagonian andes" OR "park range" OR "paria plateau" OR "pare mts" OR "páramos de la lora" OR "páramo de sumapaz" OR "páramo de pisba" OR "páramo de cruz verde" OR "paradise range" OR "paparoa range" OR "pangong range" OR "pancake range" OR "panamint range" OR "pan ling" OR "pampa del tamarugal" OR "pamir mts" OR "palatinate forest" OR "palani hills" OR "palamalai hills" OR "pakistan himalaya" OR "pahute mesa" OR "pahranagat range" OR "pah rah range" OR "paeroa range" OR "pacific ranges" OR "pacific coast ranges" OR "pachaimalai hills" OR "pab range" OR "ozark plateau" OR "ozark foothills" OR "owen stanley range" OR "outer western carpathians" OR "outer eastern carpathians" OR "ougarta range" OR "ouarsenis range" OR "ouarsenis massif" OR "ötztal alps" OR "otway ranges" OR "otago hills" OR "orulgan ridge" OR "orulgan range" OR "ortler alps" OR "oregon coast range" OR "oregon cascades" OR "ore mts" OR "ordos plateau" OR "orange river valley" OR "old man range" OR "oku massif" OR "okanogan range" OR "obudu plateau" OR "oban hills" OR "nyika plateau" OR "nyenchen tanglha" OR "nyambene hills" OR "nyainqentanglha shan" OR "nuratau range" OR "nun kun" OR "nulato hills" OR "noyon uul" OR "northwestern pyrenees" OR "northwestern caucasus" OR "northwestern altai" OR "northwestern alps" OR "northwest adirondacks" OR "northern zagros" OR "northern wind river range" OR "northern western ghats" OR "northern wasatch range" OR "northern virginia blue ridge" OR "northern urals" OR "northern tyrolean limestone alps" OR "northern tablelands" OR "northern schwarzwald" OR "northern scandes" OR "northern sawatch range" OR "northern sangre de cristo range" OR "northern rockies" OR "northern ranges" OR "northern range" OR "northern pindus" OR "northern patagonian andes" OR "northern oregon coast range" OR "northern madison range" OR "northern karakorum" OR "northern greater khingan" OR "northern front ranges" OR "northern front range" OR "northern cumberland plateau" OR "northern california coast ranges" OR "northern blue ridge" OR "northern black hills" OR "northern black forest" OR "northern bitterroot range" OR "northern bakony" OR "northern appalachians" OR "northern apennines" OR "northern andes" OR "northern altai" OR "northern alps" OR "northern allegheny plateau" OR "northern adirondacks" OR "northern absaroka range" OR "northeastern pyrenees" OR "northeastern massif central" OR "northeastern dolomites" OR "northeastern altai" OR "northeastern alps" OR "north york moors" OR "north western alps" OR "north range" OR "north pennines" OR "north patagonian massif" OR "north pare" OR "north island volcanic plateau" OR "north eastern caucasus" OR "north eastern alps" OR "north central zagros" OR "north cascades" OR "north canterbury hills" OR "norra storfjället" OR "nopah range" OR "nizke tatry" OR "niut range" OR "nisling range" OR "nimba range" OR "nightcap range" OR "nicholas range" OR "ngong hills" OR "newton ridge" OR "newton range" OR "new zealand uplands" OR "new pass range" OR "new england uplands" OR "new england upland" OR "nepal himalaya" OR "néouvielle massif" OR "needle range" OR "nanling mts" OR "nanga parbat" OR "nandi hills" OR "nan shan" OR "nan ling" OR "namuli massif" OR "namanga hills" OR "nallamala hills" OR "nakhon si thammarat range" OR "nakai plateau" OR "naga hills" OR "myjava hills" OR "muzkol range" OR "musgrave ranges" OR "murchison range" OR "muránska planina" OR "muntanyes de prades" OR "mummy range" OR "mt trumbull" OR "mt thielsen" OR "mt shasta" OR "mt rogers" OR "mt robson" OR "mt rainier" OR "mt princeton" OR "mt olympus" OR "mt marcy" OR "mt kenya" OR "mt jefferson" OR "mt hood" OR "mt erciyes" OR "mt elgon" OR "mt daniel" OR "mt cameroon" OR "mt baldy" OR "mt ararat" OR "mt adams" OR "mount yale" OR "mount whitney" OR "mount washington" OR "mount trumbull" OR "mount tongariro" OR "mount tianmu" OR "mount thielsen" OR "mount tambora" OR "mount tai" OR "mount st* helens" OR "mount spokane" OR "mount shasta" OR "mount saint helens" OR "mount sabalan" OR "mount rundle" OR "mount ruapehu" OR "mount rogers" OR "mount robson" OR "mount rinjani" OR "mount rainier" OR "mount princeton" OR "mount pilchuck" OR "mount paiko" OR "mount ossa" OR "mount olympus" OR "mount nemrut" OR "mount namuli" OR "mount moosilauke" OR "mount meru" OR "mount meager" OR "mount massive" OR "mount marsabit" OR "mount marcy" OR "mount mansfield" OR "mount malindang" OR "mount lyell" OR "mount lofty ranges" OR "mount lebanon" OR "mount lebanon range" OR "mount lao" OR "mount kupe" OR "mount kulal" OR "mount kenya" OR "mount jefferson" OR "mount hood" OR "mount heng" OR "mount hasan" OR "mount harvard" OR "mount evans" OR "mount etna" OR "mount erciyes" OR "mount emei" OR "mount elgon" OR "mount elbert" OR "mount edith cavell" OR "mount drummond" OR "mount deception" OR "mount cook range" OR "mount chocorua" OR "mount cameroun" OR "mount cameroon" OR "mount cameron" OR "mount buffalo" OR "mount baldy" OR "mount athos" OR "mount assiniboine" OR "mount ararat" OR "mount aragats" OR "mount aix" OR "mount adams" OR "mosquito range" OR "moroccan atlas" OR "morgan range" OR "moravian-silesian beskids" OR "moorfoot hills" OR "monument peaks" OR "monts groulx" OR "monts faucilles" OR "monts du lyonnais" OR "monts du forez" OR "monts du cantal" OR "monts du beaujolais" OR "monts doré" OR "monts de saïda" OR "monts de lacaune" OR "monts de la madeleine" OR "monts de daïa" OR "monts d'arrée" OR "monti simbruini-ernici" OR "monti sicani" OR "monti sibillini" OR "monti sabini" OR "monti reatini" OR "monti prenestini" OR "monti picentini" OR "monti lepini" OR "monti lattari" OR "monti della laga" OR "monti della daunia" OR "monti carseolani" OR "montes vascos" OR "montes universales" OR "montes obarenes" OR "montes de vitoria" OR "montes de triano" OR "montes de toledo" OR "montes de maría" OR "montes de león" OR "montes de castejón" OR "monte ferru" OR "monte cristo range" OR "monte argentario" OR "monte arci" OR "monte albo" OR "montagnes noires" OR "montagnes françaises" OR "montagne pelée" OR "montagne noire" OR "montagne bourbonnaise" OR "mont péko" OR "mont blanc range" OR "mont blanc massif" OR "mono craters" OR "monitor range" OR "mongun taiga" OR "mongun taiga massif" OR "mongolian plateau" OR "mongolian altai" OR "monche tundra" OR "moldavian plateau" OR "mokra gora" OR "mogollon rim" OR "mitchell ranges" OR "mitchell range" OR "mission ridge" OR "mission range" OR "mishmi hills" OR "miri hills" OR "mineral mts" OR "mikir hills" OR "middle urals" OR "middle ural*" OR "middle atlas" OR "mid north coast" OR "michigan upper peninsula" OR "miao ling" OR "metaliferi mts" OR "meseta patagónica" OR "meseta de somuncurá" OR "meseta central" OR "mescal range" OR "mendip hills" OR "melville range" OR "meliau range" OR "mekongga mts" OR "medicine range" OR "mcpherson range" OR "mcilwraith range" OR "mccullough range" OR "mbooni hills" OR "mbeya range" OR "matobo hills" OR "mataketake range" OR "massive range" OR "massif du nord" OR "massif du carlit" OR "massif du canigou" OR "massif des bornes" OR "massif des arbailles" OR "massif des albères" OR "massif de l'arize" OR "massif de l'agly" OR "massif de la selle" OR "massif de la hotte" OR "massif central" OR "marojejy massif" OR "maritime alps" OR "marble range" OR "mantiqueira range" OR "maneau range" OR "mambilla plateau" OR "malvern hills" OR "malte brun range" OR "maligne range" OR "mala kapela" OR "makonde plateau" OR "major caucasus" OR "main ridge" OR "main range" OR "main caucasian range" OR "mahogany hills" OR "mahale mts" OR "mahadeo hills" OR "madison range" OR "madison plateau" OR "macizo nipe sagua baracoa" OR "macizo de teno" OR "macizo de somiedo" OR "macizo de posets" OR "macizo de montseny" OR "macizo de la maladeta" OR "macizo de cotiella" OR "macizo de anaga" OR "macizo colombiano" OR "maciço do itatiaia" OR "macgillycuddy's reeks" OR "macedon ranges" OR "macdonnell ranges" OR "lyngen alps" OR "lushai hills" OR "lu shan" OR "low tatras" OR "low tatra*" OR "low beskids" OR "low beskid*" OR "low beskid" OR "lovozero massif" OR "lost river range" OR "los montes de málaga" OR "los altos de chiapas" OR "lord range" OR "longwood range" OR "longshou shan" OR "longs peak" OR "longmen mts" OR "long shan" OR "loita hills" OR "lizard range" OR "livingstone range" OR "livingston range" OR "liverpool range" OR "little carpathians" OR "little beskids" OR "little atlas" OR "lisburne hills" OR "limestone ridge" OR "limestone massif" OR "ligurian prealps" OR "ligurian apennines" OR "ligurian alps" OR "lianhua shan" OR "leucite hills" OR "lesser khingan" OR "lesser himalaya" OR "lesser caucasus" OR "lepontine alps" OR "lenglong ling" OR "lena-angara plateau" OR "lena plateau" OR "lemhi range" OR "lely mts" OR "lechtal alps" OR "leaota mts" OR "lateral range" OR "last chance range" OR "lassen peak" OR "las vegas range" OR "large caucasus" OR "lao shan" OR "lammermuir hills" OR "lammermoor range" OR "lamb range" OR "lake range" OR "lake plateau" OR "laji shan" OR "ladakh range" OR "labwor hills" OR "la sila" OR "la serrota" OR "la serra d'en galceran" OR "kysucké beskydy" OR "kyrgyz range" OR "kyrgyz ala-too range" OR "kyrgyz ala too" OR "kwazulu natal midlands" OR "kuznetsk alatau" OR "kurai ridge" OR "kunlun range" OR "kunlun mts" OR "kungey alatau" OR "kun lun" OR "kumaun lesser himalayas" OR "kumaun himalaya" OR "kumaon himalaya*" OR "kular ridge" OR "kubor range" OR "krušné hory" OR "krupinská planina" OR "kroombit tops" OR "krivánska malá fatra" OR "kozie chrbty" OR "koryak range" OR "koppeh dagh" OR "kopet dagh" OR "kopet dag" OR "kongur shan" OR "kondoa irangi hills" OR "kondapalli hills" OR "kolyma upland" OR "kolvitsa massif" OR "kolli hills" OR "kokanee range" OR "kodar ridge" OR "kodar range" OR "kluane ranges" OR "kitchen range" OR "kirthar range" OR "kingston range" OR "khumbu himal" OR "khibiny massif" OR "khentei ridge" OR "khatipara ridge" OR "khasi hills" OR "kharaulakh range" OR "khangai mts" OR "keszthely mts" OR "kazakh uplands" OR "kawich range" OR "kaweka range" OR "kawdy plateau" OR "karpaty zachodnie" OR "karnataka plateau" OR "karlik range" OR "karen hills" OR "karatau ridge" OR "karatau range" OR "kangchenjunga himal" OR "kamnik–savinja alps" OR "kamnik alps" OR "kambui hills" OR "kamasia hills" OR "kalrayan hills" OR "kalba range" OR "kaimanawa range" OR "kaimai range" OR "kaikoura ranges" OR "kaibab plateau" OR "kachin hills" OR "jura mts" OR "juneau icefield" OR "julian prealps" OR "julian alps" OR "jos plateau" OR "jervois range" OR "jebel tuwaiq" OR "jebel sinjar" OR "jebel saghro" OR "jebel bishri" OR "jebel amour" OR "jebel akhdar" OR "jebal-e barez" OR "jebal barez" OR "jbel saghro" OR "jawadhu hills" OR "jawadhi hills" OR "javadi hills" OR "japanese alps" OR "james peak" OR "jaintia hills" OR "jabal sinjar" OR "jabal akhdar" OR "iveti hills" OR "italy range" OR "italian peaks" OR "italian group" OR "italian alps" OR "isalo massif" OR "iranian plateau" OR "inner western carpathians" OR "inland kaikoura range" OR "indian peaks" OR "indian peak range" OR "indian himalayan region" OR "idanre hills" OR "hunua ranges" OR "hume range" OR "humboldt range" OR "huila plateau" OR "house range" OR "hot springs range" OR "hot creek range" OR "horseranch range" OR "horse range" OR "horse heaven hills" OR "homathko icefield" OR "hokonui hills" OR "hohonu range" OR "hoggar massif" OR "hoback range" OR "hissar range" OR "hindu raj" OR "hindu kush" OR "hindu kush himalayan region" OR "hindu kush himalaya*" OR "hindu kush himalaya* region" OR "himachal himalayas" OR "hill country" OR "high tauern" OR "high tatras" OR "high tatra*" OR "high atlas" OR "high atlas range" OR "hidaka range" OR "herveys range" OR "hervey range" OR "herberton range" OR "henry mts" OR "hengduan shan" OR "heng shan" OR "heli shan" OR "helan shan" OR "helan range" OR "hayes range" OR "haut tell tunisien" OR "harz national park" OR "harts range" OR "hart ranges" OR "harrat al-sham" OR "harghita mts" OR "handies peak" OR "hamiguitan range" OR "hamersley range" OR "hall range" OR "haldon hills" OR "hakarimata range" OR "gurktal alps" OR "guayana shield" OR "gros ventre range" OR "grey range" OR "gregory rift" OR "greenwater range" OR "greenhorn range" OR "greater khingan" OR "greater caucasus" OR "great western tiers" OR "great rift valley" OR "great range" OR "great escarpment" OR "great dividing range" OR "great basin ranges" OR "grays range" OR "grays peak" OR "grays hills" OR "gray hills" OR "gravelly range" OR "grant range" OR "granite range" OR "grands causses" OR "grandes rousses" OR "grand mesa" OR "grand canyon" OR "gran sasso" OR "gran paradiso" OR "grampian range" OR "graian alps" OR "gore range" OR "gorce mts" OR "gold range" OR "goat rocks" OR "goat range" OR "glens of antrim" OR "glenlyon range" OR "glarus alps" OR "glacier peak" OR "gissar range" OR "gillis range" OR "gibraltar range" OR "german alps" OR "german alpine foreland" OR "gerecse mts" OR "georgian caucasus" OR "georgia blue ridge" OR "gelibolu yarimadasi" OR "gawler ranges" OR "gausdal vestfjell" OR "garo hills" OR "garnet range" OR "garhwal himalaya" OR "garfield peaks" OR "garfield hills" OR "gaoligong shan" OR "gaoligong mts" OR "gangdise range" OR "ganesh himal" OR "gammon ranges" OR "gallatin range" OR "gailtal alps" OR "gabilan range" OR "gabbs valley range" OR "fuuta jaloo" OR "futa jallon" OR "futa djalon" OR "fuentes carrionas" OR "fruška gora" OR "front range" OR "frenchman hills" OR "french pyrenees" OR "french jura" OR "french alps" OR "fraser plateau" OR "franconian jura" OR "fra cristobal range" OR "fox range" OR "fouta djallon" OR "flint hills" OR "flint creek range" OR "flinders range" OR "flathead range" OR "flat tops" OR "fish springs range" OR "fish lake plateau" OR "fish creek range" OR "first range" OR "finisterre range" OR "fila brunqueña" OR "fergana range" OR "fennoscandian shield" OR "fell lapland" OR "fawnie range" OR "farallones de cali" OR "falaise de banfora" OR "fairweather range" OR "făgăraș mts" OR "everard ranges" OR "european upland*" OR "european alps" OR "etna volcan*" OR "etna mount*" OR "epi island" OR "ely range" OR "elk ridge" OR "elk range" OR "elgeyo escarpment" OR "elbow range" OR "el garraf" OR "eje neovolcanico mexicano" OR "egan range" OR "edwards plateau" OR "edough massif" OR "ecuadorian andes" OR "eastern tian shan" OR "eastern tatras" OR "eastern tannu ola" OR "eastern sudetes" OR "eastern sayan" OR "eastern satpura range" OR "eastern rift" OR "eastern ridge" OR "eastern rhodopes" OR "eastern range" OR "eastern pyrenees" OR "eastern pre-pyrenees" OR "eastern pamirs" OR "eastern middle atlas" OR "eastern main range" OR "eastern kunlun" OR "eastern hindu kush" OR "eastern hills" OR "eastern high atlas" OR "eastern hellenides" OR "eastern hajar" OR "eastern ghat" OR "eastern ghats" OR "eastern escarpment" OR "eastern coastal range" OR "eastern caucasus" OR "eastern carpathians" OR "eastern brooks range" OR "eastern betics" OR "eastern beskids" OR "eastern arc mts" OR "eastern anti-atlas" OR "eastern alps" OR "eastern adirondacks" OR "east* carpathian*" OR "east usambara" OR "east range" OR "east kunlun" OR "east humboldt range" OR "east african plateau" OR "dzungarian alatau" OR "dzhugdzhur range" OR "dugway range" OR "duck creek range" OR "dry andes" OR "drummond range" OR "douglas ranges" OR "douglas range" OR "dorsale tunisienne" OR "dorsal gallega" OR "domeyko range" OR "dolomitic alps" OR "dolomiti lucane" OR "dogtooth range" OR "dobrogea plateau" OR "djebel sinjar" OR "djebel amour" OR "dinaric alps" OR "dinara massif" OR "dieng volcanic complex" OR "dieng plateau" OR "diamond range" OR "dial range" OR "diablo range" OR "dhaulagiri himalaya" OR "dhauladhar range" OR "dévoluy massif" OR "deserters range" OR "desert range" OR "deseado massif" OR "deosai plateau" OR "deli jovan" OR "defiance plateau" OR "deep creek range" OR "deccan plateau" OR "dean hill" OR "daxue shan" OR "dawson range" OR "darvaz range" OR "darling range" OR "daqing shan" OR "danghe nanshan" OR "danghe nan shan" OR "dandenong ranges" OR "dafla hills" OR "dachstein mts" OR "dabie mts" OR "daba shan" OR "da hinggan" OR "da hinggan range" OR "da hinggan ling" OR "da ba shan" OR "czech carpathians" OR "cypress hills" OR "cumbres calchaquíes" OR "cumberland plateau" OR "culebra range" OR "crocker range" OR "crimean mts" OR "crawford range" OR "craigieburn range" OR "cottian alps" OR "cordón del portillo" OR "cordón del plata" OR "conondale range" OR "confusion range" OR "columbia plateau" OR "columbia icefield" OR "columbia hills" OR "colorado rockies" OR "colorado plateau" OR "coconino plateau" OR "coast range" OR "cleopatra's needle" OR "clent hills" OR "clemenceau icefield" OR "clear range" OR "clear hills" OR "clayton ridge" OR "clark range" OR "cimarron range" OR "chyulu hills" OR "churia hills" OR "chulyshman plateau" OR "chukotka upland" OR "chugach range" OR "chota nagpur plateau" OR "chola shan" OR "choč mts" OR "chittagong hill tracts" OR "chiricahua peak" OR "chinese wall" OR "chinese loess plateau" OR "chin hills" OR "chimantá massif" OR "chilkat range" OR "chilcotin ranges" OR "chilcotin plateau" OR "chiemgau alps" OR "chichester ranges" OR "cheviot hills" OR "chersky ridge" OR "chersky range" OR "cherry creek range" OR "cherang'any hills" OR "chatkal range" OR "chartreuse massif" OR "chapada dos veadeiros" OR "chapada dos guimarães" OR "chapada do araripe" OR "chapada diamantina" OR "chapada das mesas" OR "chapada das mangabeiras" OR "changbai mts" OR "chaîne des puys" OR "chaîne des matheux" OR "chagai hills" OR "cerros orientales" OR "cerros del sira" OR "cerros de escazú" OR "cerros colorados" OR "cerro guaiquinima" OR "cerro duida" OR "centre hills" OR "central-eastern alps" OR "central wind river range" OR "central western ghats" OR "central wasatch range" OR "central volcanic zone" OR "central venezuelan andes" OR "central uplands" OR "central tian shan" OR "central tell atlas" OR "central taurus" OR "central tablelands" OR "central sudetes" OR "central southern pyrenees" OR "central siberian plateau" OR "central ranges" OR "central range" OR "central pyrenees" OR "central pre-pyrenees" OR "central plateau" OR "central pindus" OR "central oregon coast range" OR "central north caucasus" OR "central moravian carpathians" OR "central middle atlas" OR "central mexican plateau" OR "central massif" OR "central massif central" OR "central madison range" OR "central lesser himalaya*" OR "central kunlun" OR "central japanese alps" OR "central himalaya*" OR "central high plateau" OR "central high atlas" OR "central front ranges" OR "central front range" OR "central cumberland plateau" OR "central caucasus" OR "central betics" OR "central balkan mts" OR "central appalachians" OR "central apennines" OR "central anti-atlas" OR "central andes" OR "central america volcanic arc" OR "central alps" OR "central alborz mts" OR "centennial range" OR "ceahlău massif" OR "causses du quercy" OR "caucasus mts" OR "caucasus major" OR "cathedral range" OR "cathedral group" OR "catena costiera" OR "catalan coastal range" OR "cascade* range" OR "cascade range" OR "carter range" OR "carson range" OR "carso huasteco" OR "carpathian mts" OR "carnic prealps" OR "carnic alps" OR "cariboo plateau" OR "cardamom mts" OR "cardamom hills" OR "capitol reef" OR "capitol hills" OR "cape midlands" OR "canyon ranges" OR "cantabrian range" OR "cantabrian mts" OR "candelaria hills" OR "canadian shield" OR "canadian rockies" OR "canadian appalachians" OR "campbell range" OR "campbell hills" OR "campanian apennines" OR "cameroon line" OR "camelsfoot range" OR "călimani mts" OR "california coast ranges" OR "california cascades" OR "calar del río mundo" OR "calabrian apennines" OR "burster range" OR "bureya massif" OR "bungo range" OR "bullfrog hills" OR "bukit tigapuluh" OR "bukit barisan" OR "buffalo hills" OR "buda hills" OR "buckskin range" OR "bucegi mts" OR "browning range" OR "brooks range" OR "broken hills" OR "brindabella range" OR "bridger range" OR "brenta group" OR "brecon beacons" OR "brazilian plateau" OR "brazeau range" OR "boz dag*" OR "bowland fells" OR "bow range" OR "boundary ranges" OR "bosche range" OR "bornes massif" OR "border ranges" OR "borborema plateau" OR "book cliffs" OR "bong range" OR "bonar range" OR "bomi hills" OR "bolu massif" OR "bolsón de mapimí" OR "bolivian plateau" OR "bolivian andes" OR "bolaven volcanic field" OR "bolaven plateau" OR "bohemian massif" OR "bohemian forest" OR "bohea hills" OR "bogotá savanna" OR "bogong high plains" OR "bogda shan" OR "bogd uul" OR "blue range" OR "blue mts" OR "bloods range" OR "blidean atlas" OR "blackwater range" OR "blackall range" OR "black rock range" OR "black range" OR "black hills" OR "black forest" OR "black divide" OR "biu plateau" OR "bitterroot range" OR "bismarck range" OR "binalud range" OR "bihor mts" OR "bighorn range" OR "big salmon range" OR "big range" OR "big caucasus" OR "bhutan himalaya" OR "betic system" OR "betic chain" OR "baetic system" OR "baetic chain" OR "beskid sadecki" OR "bernina massif" OR "bernese alps" OR "berkshire hills" OR "bergamo alps" OR "berchtesgaden alps" OR "benton range" OR "bendor range" OR "ben ohau range" OR "belted range" OR "bellenden ker range" OR "belledonne massif" OR "belianske tatras" OR "bei shan" OR "bayuda volcanic field" OR "bayan har shan" OR "bavarian forest" OR "bavarian alps" OR "bauges massif" OR "bateke plateau" OR "barrington tops" OR "barrier range" OR "barnett hills" OR "barisan range" OR "barguzin ridge" OR "barguzin range" OR "barail range" OR "bannock range" OR "banjaran crocker" OR "bandiagara escarpment" OR "banded range" OR "bamenda grassfields" OR "baltic shield" OR "ball range" OR "balkan range*" OR "balkan mts" OR "baldy peak" OR "balaton uplands" OR "bakossi mts" OR "bailadila range" OR "baikal ridge" OR "baikal range*" OR "baetic range*" OR "betic range*" OR "badzhal range" OR "backbone ranges" OR "baba budan hills" OR "austrian central alps" OR "austrian alps" OR "australian alps" OR "aurora range" OR "atlas range*" OR "atlas marocain" OR "atlas chain*" OR "atlas blidéen" OR "atlantic plateau" OR "atewa range" OR "atbashi range" OR "asturian massif" OR "assam himalaya" OR "aspen range" OR "arthur range" OR "arrowsmith range" OR "arrow canyon range" OR "armorican massif" OR "arjin shan" OR "argentinian andes" OR "arctic ocean islands" OR "aravalli range" OR "aravali range" OR "araucaria plateau" OR "arakan yoma" OR "arakan hills" OR "aragonese pyrenees" OR "aquarius plateau" OR "apuseni mts" OR "apuan alps" OR "aprada tepui" OR "appalachian* range" OR "appalachian ridges" OR "appalachian plateaus" OR "aorangi range" OR "anvil range" OR "antrim plateau" OR "anti taurus" OR "antelope range" OR "annapurna range" OR "annapurna himalaya" OR "ankaratra massif" OR "angolan escarpment" OR "andringitra massif" OR "andes peruanos" OR "andes fueguinos" OR "andean plateau" OR "anatolian plateau" OR "anaimalai hills" OR "anaconda range" OR "anabar plateau" OR "amne machin" OR "ammergauer alpen" OR "ammergau alps" OR "alxa plateau" OR "altyn tagh" OR "altun shan" OR "altun mts" OR "altos de jalisco" OR "altiplano cundiboyacense" OR "altay sayan" OR "altay range" OR "altai sayan" OR "altai range" OR "altai mts" OR "alpine-himalayan orogenic belt" OR "alpine-himalayan belt" OR "alpine lakes area" OR "alpide belt" OR "alpi orobie" OR "alpes maritimes" OR "almăj depression" OR "allies group" OR "allgäu alps" OR "allegheny plateau" OR "allegheny front" OR "alishan national scenic area" OR "algerian atlas" OR "alexander range" OR "alexander archipelago" OR "aleutian ridge" OR "aleutian range" OR "aleutian arc" OR "aleut* islands" OR "aldan plateau" OR "alborz mts" OR "albion range" OR "alaska range" OR "alashan plateau" OR "ajodhya hills" OR "ailao shan" OR "ailao mts" OR "aiguilles rouges" OR "aiguilles rouges massif" OR "aibga ridge" OR "ahaggar mts" OR "adrar des ifoghas" OR "adobe range" OR "adirondack mts" OR "adirondack high peaks" OR "adelbert range" OR "adamawa plateau" OR "adamawa massif" OR "absaroka range" OR "abruzzo apennine*" OR "abruzzi apennines" OR "aberdare range" OR "jebel tessala" OR rockies OR andes OR betics OR himalaya OR appalachian* OR andean OR caucasus OR urals OR carpathians OR himachal OR apennine* OR apennines OR pyrenees OR jura OR tianshan OR qinling OR adirondacks OR altiplano OR zagros OR ardennes OR vosges OR kunlun OR babor* OR kilimanjaro OR witwatersrand OR tatras OR alborz OR palisades OR vesuvius OR harz OR nilgiris OR karakoram OR guadarrama OR qiangtang OR pennines OR anti-atlas OR vercors OR beishan OR cévennes OR grampians OR sudetes OR morvan OR sikhote-alin OR dinarides OR drakensberg OR schwarzwald OR teide OR karpaty OR karakorum OR fiordland OR beskids OR nanshan OR ruapehu OR altyn-tagh OR pieniny OR velebit OR uludağ OR canyonlands OR cotswolds OR waterberg OR katahdin OR jinggangshan OR laoshan OR rondane OR hindukush OR vanoise OR emmental OR cairngorms OR taunus OR kohala OR medvednica OR odenwald OR pilat OR adamello OR tatry OR pollino OR ecrins OR pirin OR gorce OR hunsrück OR yinshan OR mecsek OR hellenides OR sabalan OR trans-himalaya OR djurdjura OR alay OR arves OR majella OR ruwenzori OR jirisan OR aubrac OR belledonne OR snowdonia OR breheimen OR jotunheimen OR bakony OR madonie OR burren OR daqingshan OR udzungwa OR sauerland OR soutpansberg OR spessart OR burda OR scandes OR chablais OR katla OR matese OR nebrodi OR papuk OR alishan OR hardangervidda OR pelion OR cassiars OR fenghuangshan OR monfragüe OR aspromonte OR westerwald OR kopaonik OR cederberg OR brandberg OR vogelsberg OR cangshan OR gorongosa OR nemrut OR nassa OR gaoligongshan OR sahand OR taillefer OR kalnik OR tienshan OR mengshan OR biokovo OR tjörnes OR nyainqentanglha OR dahra OR svartisen OR parnassos OR dayaoshan OR karadağ OR gallot OR vitosha OR pamir-alai OR mátra OR pilanesberg OR ségala OR pre-pyrenees OR kebnekaise OR blouberg OR hualalai OR edough OR rooiberg OR korab OR vourinos OR karadag OR sumava OR putorana OR elburz OR tafelberg OR okstindan OR durmitor OR valdres OR verno OR appennini OR donggan OR bozburun OR behistun OR učka OR xueshan OR yaoshan OR bibans OR khamar-daban OR karasjok OR winterberg OR alburni OR chornohora OR cézallier OR baviaanskloof OR planèze OR changthang OR tsinling OR dovrefjell OR giffre OR cedarberg OR jesenik OR kozjak OR warrumbungles OR jablanica OR gennargentu OR changtang OR zarkos OR kopetdag OR crestones OR magaliesberg OR pamir-alay OR langeberg OR lauzière OR daliangshan OR andringitra OR funiushan OR ukaguru OR transhimalaya OR gesäuse OR sidobre OR ozren OR presanella OR gariwerd OR svilaja OR macelj OR beaufortain OR prokletije OR fongen OR krndija OR finnmarksvidda OR dilj OR mariovo OR psunj OR swartruggens OR jastrebac OR ankaratra OR hardangerjøkulen OR karwendel OR anti-lebanon OR beni-chougrane OR branisko OR tribeč OR piketberg OR stolowe OR yamanlar OR kithairon OR parnitha OR poľana OR pratomagno OR slavyanka OR mosor OR mavrovouni OR titteri OR atakor OR galičica OR alburz OR brownsberg OR grammos OR hochschwab OR mainpat OR saltfjellet OR artense OR rtanj OR rätikon OR chataigneraie OR auyantepui OR rumija OR kasikasima OR wolkberg OR ograzhden OR drauzug OR mianshan OR majevica OR svydovets OR chitteri OR chřiby OR žiar OR anyemaqen OR tavan-bogd OR gantrisch OR lovćen OR bleia OR forollhogna OR tomaros OR gasherbrum OR lévézou OR storskarven OR haukelifjell OR aladaglar OR khibins OR trollheimen OR kvamshesten OR nordfjella OR daxueshan OR sinjajevina OR beljanica OR fangdoushan OR kožuf OR hualālai OR tsambagarav OR jinpingshan OR diamir OR jakupica OR sarisariñama OR qangtang OR tzoumerka OR vindelfjällen OR elborz OR golak OR escandorgue OR xerovouni OR ryfylkeheiane OR ljubišnja OR carpati OR aladagh OR malundwe OR orjen OR sunndalsfjella OR drakensberge OR waifangshan OR sausfjellet OR javorie OR jiulingshan OR ottadalen OR kukavica OR anysberg OR suurberg OR blåfjella OR kugitangtau OR hamar-daban OR norefjell OR vlahina OR gruvefjellet OR tsagan-daban OR filefjell OR romsdalsalpane OR stølsheimen OR sokoró OR maleshevo OR yengisogat OR babatag OR antichasia OR anti-apennines OR karawanks OR ammergebirge OR cserhat OR toven OR långfjället OR goljak OR blefjell OR bogdashan OR erimanthos OR skjækerfjella OR høgtuva OR ålfotbreen OR meygal OR borzsony OR ambohiby OR kharkhiraa OR charchiraa OR murovdag OR steenkampsberg OR tsinling-dabashan OR jurjura OR kjølhaugan OR orvilos OR valamara OR pentadactylos OR plješivica OR artfjället OR roggeveldberge OR tendurek OR skaget OR ilfjellet OR trysilfjellet OR pothigai OR slievenamon OR maumturks OR kamakou OR vtacnik OR jabllanicë OR jundushan))
